# Supplementary material for: Computational engineering of the polyester hydrolase PHL7 for efficient poly(ethylene terephthalate) degradation in biocatalytic recycling processes
Source: Nat Commun. 2026 May 15;17:4370. doi: 10.1038/s41467-026-70868-4 (PMC13179365; doi:10.1038/s41467-026-70868-4)
Supplement: Supplementary file 1 — Supplementary Information File [file 41467_2026_70868_MOESM1_ESM.pdf]

Supplementary Information for

Computational engineering of the polyester hydrolase PHL7 for efficient poly(ethylene terephthalate) degradation in biocatalytic recycling processes

Paula Blazquez-Sanchez[1,#], Jonas Gunkel[1,#], Abibe Useini[1,2,#], Alexander Zlobin[1], Jonathan D Zakary[1], Andrea Schöler[1], Norbert Graefe[1,3], Felipe Engelberger[1], Filipa Cantanhede[1], Ronny Frank[4], Ziyue Zhao[5], Afsaneh Zarei[5], Erik Butenschön[5], Jörg Matysik[5], Wolfgang Zimmermann[5], Norbert Sträter[2], Christian Sonnendecker[5,*], Georg Künze[1,3,6,*]

[1] Institute for Drug Discovery, Leipzig University, 04103 Leipzig, Germany

[2] Institute of Bioanalytical Chemistry, Centre for Biotechnology and Biomedicine, Leipzig University, 04103 Leipzig, Germany

[3] Interdisciplinary Center for Bioinformatics, Leipzig University, 04107 Leipzig, Germany

[4] Centre for Biotechnology and Biomedicine, Biochemical Cell Technology, Leipzig University, 04103 Leipzig, Germany

[5] Institute of Analytical Chemistry, Leipzig University, 04103 Leipzig, Germany

[6] Center for Scalable Data Analytics and Artificial Intelligence, Leipzig University, 04105 Leipzig, Germany

# These authors contributed equally

*Correspondence: christian.sonnendecker@uni-leipzig.de, georg.kuenze@uni-leipzig.de

Table of Contacts

Supplementary Methods.......... 3

Supplementary MD simulation methods.......... 3

Supplementary description on the design of R1 and R2 variants 4

Supplementary Tables

Supplementary Table 1. 6
Supplementary Table 2. 7
Supplementary Table 3. 18
Supplementary Table 4. 19
Supplementary Table 5. 22

Supplementary Figures

Supplementary Figure 1. 23
Supplementary Figure 2. 23
Supplementary Figure 3. 24
Supplementary Figure 4. 24
Supplementary Figure 5. 25
Supplementary Figure 6. 26
Supplementary Figure 7. 27
Supplementary Figure 8. 27
Supplementary Figure 9. 28
Supplementary Figure 10. 29
Supplementary Figure 11. 30
Supplementary Figure 12. 31
Supplementary Figure 13. 32
Supplementary Figure 14. 33
Supplementary Figure 15. 34
Supplementary Figure 16. 35
Supplementary Figure 17. 35
Supplementary Figure 18. 36
Supplementary Figure 19. 37
Supplementary Figure 20. 38
Supplementary Figure 21. 39
Supplementary Figure 22. 40

Supplementary References 40

Supplementary Methods

Supplementary MD simulation methods

Adjustments for substrate force field parameters: Oxyanion holes relax van der Waals repulsion between the attacking nucleophile and the attacked carbonyl carbon atom in the noncovalent complex (1), and such behavior is not covered by force fields. To estimate the effect of it on our capabilities to correctly model PHL7-substrate complexes, we first used QM/MM simulations and compared them to unmodified MM treatment. We used the Gromacs-DFTBplus interface (2). The QM subsystem consisted of MHET, side chains of His209 and Asp177, full residues Ser131 and Gly133, backbone of Met132. All backbone segments were extended to have QM/MM cuts only before CA atoms, for which Amber charge correction scheme was applied (3). QM atoms were modeled with GFN2-xTB (4) known to provide good geometries. To facilitate the description of the bound geometry, we used well-tempered metadynamics simulations with 8 walkers running for 200 ps with 1 fs time step. The potential was applied to the distance between OG(Ser131) and the carbonyl of MHET with the height 0.5 kJ/mol/nm^2 and the width of 0.005 nm each 200 steps. Bias factor was set to 10. We used Tiwary reweighting on the last 100 ps to construct 2D free energy surfaces in the space of the driving variable as the first axis and the distance between N(Met132) and O(MHET) as the second axis. Similar profiles were built for pure MM treatment with and without corrections for comparison. Corrections were purposefully made simple by introducing a Lennard-Jones exclusion between OG(Ser131) and C(MHET) following the reference (1), and then upscaling their Coulomb attraction by a factor of 1.21. These corrections significantly improved the description of the interaction geometry (Supplementary figure 8a)

Hamiltonian replica exchange (HREX) simulations: We performed HREX as a combination of two experiments for each system to improve the ability of sampling more states and draw quantitative insights. The first simulation concentrated on the transition between a loosely bound state and a "near active site" state. For it, the "hot" region comprised the whole 4x MHET molecule, and force field adjustments were turned off. The second simulation concentrated on the transition between a "near active site" state and an oxyanion hole-bound state. For it, the force field adjustments were kept, and the "hot" region consisted of 4xMHET omitting a portion of the attacked unit (Supplementary figure 9b). In both setups, we used 8 lambda windows corresponding to temperature range from 300 to 600 K ran for 500 ns each, and achieved exchange probabilities of 0.5 - 0.6 on average. Quantitative analysis was performed on the basis of a 200 – 500 ns portion of the unperturbed window. We define a "near active site" state as having OG(Ser131)-C(MHET)

distance of <5.5 Å and N(Met132)-O(MHET) distance between 5 Å and 7.5 Å. An "in-oxyanion" pre-reactive state has N(Met132)-O(MHET) distance of <5 Å.

Clustering was performed in spaces of torsion angles (Supplementary figure 9c) for "in-oxyanion" states to reduce noise and maximize interpretability and mechanistic relevance. L1 and R2 clustering was done independently since the orientations of these rings were found to be independent of each other (Supplementary figure 9d). Torsions were made non-periodic by unwrapping at the least dense region. Clustering was performed with Gaussian Mixture Models (5) owing to the multimodal normal distribution of torsions in simulations. The optimal number of clusters was determined using Bayesian information criterion (BIC).

<u>On-the-fly Probability Enhanced Sampling (OPES) simulations:</u> All OPES simulations were run in "explore" mode for 100 ns per replica in 3 replicas per system. We performed two OPES experiments independently.

Binding site conformations. We used two collective variables. The first one was a distance between Ca atoms of residues 63 and 209. The second was a distance between N(Thr64) and O(Arg92). Barrier parameter was set to 20 kJ/mol, pace to 500 steps, and sigma was allowed to go as low as 0.02 Å.

M132 sidechain conformations. We used two collective variables, chi-2 and chi-3 angles of M132 sidechain. Barrier parameter was set to 20 kJ/mol, pace to 500 steps, and sigma was allowed to go as low as 0.01 radian.

Supplementary description on the design of R1 and R2 variants

We designed nine PHL7 variants using the PROSS2 webserver (https://pross.weizmann.ac.il/). These first-round ("R1") variants contained between 8 and 40 mutations (Supplementary Table 1) and were generated in a cumulative design scheme, where each successive variant incorporated all mutations from the preceding one along with additional substitutions. Based on computational evaluation, two second-round ("R2") variants, R2M1 and R2M2, were constructed by manually combining mutations from selected R1 variants that exhibited improved Rosetta energy scores relative to both the wild-type (WT) enzyme and their respective predecessors in the design trajectory (Supplementary Figure 3). Importantly, in addition to total Rosetta energy, individual mutations were also evaluated for their structural and energetic compatibility with previously introduced substitutions.

Specifically, R2M1 combined mutations from R1M1, R1M2, R1M4, and R1M7, because these variants showed an improved or equal score relative to their predecessors in the design trajectory.

From R1M7, the mutations N113D and D196S were retained based on their predicted local stabilizing effects and compatibility with mutations introduced earlier in the design trajectory. In particular, N113D was expected to form a favorable hydrogen-bond interaction with the neighboring H109Y mutation (introduced via R1M4), while D196S was selected due to its contribution to a more neutral surface charge distribution.

R2M2 incorporated the mutations of R2M1, excluding L176N and T219I (from R1M2), together with mutations from R1M6 and R1M9, which also showed favorable energy changes. Visual inspection of the structural models for R2M1 and R2M2 was performed to identify and exclude incompatible mutations. For instance, D233N from R1M7 was omitted to preserve a putative salt bridge between residues E148K and D233, and N213T from R1M9 was excluded to maintain a stabilizing hydrogen bond with S69. L176N and T219I were only tested in the R2M1 template but not in R2M2, because these mutations occur very close to the active site (e.g. L176 is next to the catalytic D177) and we expected losses in activity from these mutations.

Supplementary Tables

Supplementary Table 1. Sequences of PROSS-designed PHL7 mutants.

Name	Sequence	Mutations compared to PHL7 WT	Number Mutations (new Mutations)	Total Rosetta Energy Change compared to PHL7 WT
R1M1	MANPYERGPDPTESSIEAVRGPFAVAQTTVSRLQADGFGGGTIY<br>YPTDTSQGTFGAVAISPGFTAGGQESIAWLGPRIASQGFVVITIDTIT<br>RLDQPDSRGRQLLAALDHLTTNSVVRNRIDPNRMAVMGHSMGG<br>GGALSAAAANNPSLKAAIPLQPWHTRKNWSSVRVPTLVVGAQLDT<br>IAPVSSHSEAFYNSLPSDLDKAYMELRGASHLVSNTPDTTTAKYS<br>IAWLKRFVDDDTRYEQFLCPAPDDPAISEYRSTCPF	**Q104L R111T T145P E148K G155P T167V L235T F248P**	8 (8)	-8.2 kcal/mol
R1M2	MANPYERGPDPTESSIEAVRGPFAVAQTTVSRLQADGFGGGTIY<br>YPTDTSQGTFGAVAISPGFTAGGQESIAWLGPRIASQGFVVITIDTIT<br>RLDQPDSRGRQLLAALDHLTTNSVVRNRIDPNRMAVMGHSMGG<br>GGALSAAAANNPSLKAAIPLQPWHTRKNWSSVRVPTLVVGAQND<br>TIAPVSSHSEAFYNSLPSDLDKAYMELRGASHLVSNTPDTTIAKY<br>SIAWLKRFVDDDTRYEQFLCPAPDDPAISEYRSTCPF	Q104L R111T T145P E148K G155P T167V **L176N T219I** L235T F248P	10 (2)	-9.6 kcal/mol
R1M3	MANPYERGPDPTESSIEAVRGPFAVAQTTVSRLQADGFGGGTIY<br>YPTDTSQGTFGAVAISPGFTATQESIAWLGPRIASQGFVVITIDTIT<br>RLDQPDSRGRQLLAALDHLTTNSVVRNRIDPNRMAVMGHSMGG<br>GGALSAAAANNPSLKAAIPLQPWHTRKNWSSVRVPTLVVGAQND<br>TIAPVSSHSEAFYNSLPSDLDKAYMELRGASHLVTNTPDTTIAKYS<br>IAWLKRFVDDDTRYEQFLCPAPDDPAISEYRSTCPF	**G66T** Q104L R111T T145P E148K G155P T167V L176N **S212T** T219I L235T F248P	12 (2)	-2.7 kcal/mol
R1M4	MANPYERGPDPTESSIEAVRGPFAVAQTTVSRLQADGFGGGTIY<br>YPTDTSQGTFGAVAISPGFTATQSSIAWLGPRIASQGFVVITIDTIT<br>RLDQPDSRGRQLLAALDYLTTNSVVRNRIDPNRMAVMGHSMGG<br>GGALSAAAANNPSLKAAIPLQPWHTRKNWSSVRVPTLVVGAQND<br>TIAPVSSHAEAFYNSLPSDLDKAYMELRGASHLVTNTPDTTIAKYS<br>IAWLKRFVDDDTRYEQFLCPAPDDPAISEYRSTCPF	G66T **E68S** Q104L **H109Y** R111T T145P E148K G155P T167V L176N **S186A** S212T T219I L235T F248P	15 (3)	-7.8 kcal/mol
R1M5	MANPYERGPDPTESSIEAARGPFAVAQTTVSRLQADGFGGGTIY<br>YPTDTSQGTFGAVAISPGFTATQSSIAWLGPRIASQGFVVITIDTN<br>TRLDQPDSRGRQLLAALDYLTTNSVVRNRIDPNRMAVMGHSMG<br>GGGALSAAAANNPSLKAAIPLQPWHTRKNWSSVRVPTLVIGAQND<br>TIAPVSSHAEAFYNSLPSDLDKAYMELRGASHLVTNTPDTTIAKYS<br>IAWLKRFVDDDTRYEQFLCPAPDDPAISEYRSTCPF	**V19A** G66T E68S **I90N** Q104L H109Y R111T T145P E148K G155P T167V **V172I** L176N S186A S212T T219I L235T F248P	18 (3)	-4.0 kcal/mol
R1M6	MANPYERGPDPTESSIEAARGPFAVAQTTVSRLQASGFGGGTIY<br>YPTDTSQGTFGAVAISPGFTATQSSIAWLGPRIASQGFVVITIDTN<br>TRLDQPDSRGRQLLAALDYLTTNSTVRDRIDPNRMAVMGHSMG<br>GGGALSAAAANNPSLKAAIPLQPWHTRKNWSSVRVPTLIIGAQND<br>TIAPVSSHAEAFYNSLPSDLDKAYMELRGASHLVTNTPNTTIAKYS<br>IAWLKRFVDDDTRYEQFLCPAPDDPAISEYRSTCPF	V19A **D36S** G66T E68S I90N Q104L H109Y R111T **V115T N118D** T145P E148K G155P T167V **V171I** V172I L176N S186A S212T **D216N** T219I L235T F248P	23 (5)	-2.0 kcal/mol
R1M7	MANPYERGPDPTESSIEAARGPFAVAQTTVSRLQASGFGGGTIY<br>YPTDTSQGTFGAVAISPGFTATQSSIAWLGPRIASQGFVVITIDTN<br>TRLDQPDSRGRQLLAALDYLTTDSTVRDRIDPNRMAVMGHSMG<br>GGGALSAAAANNPSLKAAIPLQPWHTRKNWSSVRVPTLIIGAQND<br>TIAPVSSHAEAFYNSLPSSLDKAYMELRGASHLVTNTPNTTIAKYS<br>IAWLKRFVDNDTRYEQFLCPAPDDPAISEYRSTCPF	V19A D36S G66T E68S I90N Q104L H109Y R111T **N113D** V115T N118D T145P E148K G155P T167V V171I V172I L176N S186A **D196S** S212T D216N T219I **D233N** L235T F248P	26 (3)	-6.3 kcal/mol
R1M8	MANPYERGPDPTEASIEAPRGPFAVAQTTVSRLQARGFGGGTIY<br>YPTDTSQGTFGAVAISPGFTATQSSIAWLGPRLASQGFVVITIDTN<br>TRYDQPDSRGRQLLAALDYLTTDSTVRDRIDPNRMAVMGHSMG<br>GGGALAAAANNPSLKAAIPLQPWHTRKNWSSVRVPTLIIGAQND<br>TIAPVSQHAEAFYNSLPSSLDKAYMELRGASHLVTNTPNTTIAKY<br>AIAWLKRFVDDDTRYEQFLCPAPDDPAISEYRSTCPF	**S14A V19P D36R** G66T E68S **I77L** I90N **L93Y** Q104L H109Y R111T N113D V115T N118D **S139A** T145P E148K G155P T167V V171I V172I L176N **S184Q** S186A D196S S212T D216N T219I **S223A** L235T F248P	31 (8)	-13.8 kcal/mol
R1M9	MANPYQRGPDPTEASIEAPRGPFAVAQTTVSRLDARGFGGGTIY<br>YPTDTSQGTFGAVAISPGYLATQSSIAWLGPRLASQGFVVITIDTN<br>SRYDQPDSRGRQLLAALDYLTTDSTVRDRIDPNRMAVMGHSMG<br>GGGALAAAANNPSLKAAIPLQPWHTRKDWSSVRVPTLIIGAQND<br>TIAPVSQHAEAFYNSLPSSLPKAYMELRGADHLVATTPNTTIAKYV<br>IAWLKRFVDDDTRYEQFLCPAPDDPAISEYRSTCPF	**E6Q** S14A V19P **Q34D** D36R **F63Y T64L** G66T E68S I77L I90N **T91S** L93Y Q104L H109Y R111T N113D V115T N118D S139A T145P E148K G155P **N161D** T167V V171I V172I L176N S184Q S186A D196S **D198P S208D** S212A **N213T** D216N T219I S223V L235T F248P	40 (9)	-32.9 kcal/mol
R2M1	MANPYERGPDPTESSIEAVRGPFAVAQTTVSRLQADGFGGGTIY<br>YPTDTSQGTFGAVAISPGFTAGQSSIAWLGPRIASQGFVVITIDTIT<br>RLDQPDSRGRQLLAALDYLTTDSVVRNRIDPNRMAVMGHSMGG<br>GGALSAAAANNPSLKAAIPLQPWHTRKNWSSVRVPTLVVGAQND<br>TIAPVSSHAEAFYNSLPSSLDKAYMELRGASHLVSNTPDTTIAKYS<br>IAWLKRFVDDDTRYEQFLCPAPDDPAISEYRSTCPF	E68S Q104L H109Y R111T N113D T145P E148K G155P T167V L176N S186A D196S T219I L235T F248P	15	-20.6 kcal/mol
R2M2	MANPYQRGPDPTESSIEAVRGPFAVAQTTVSRLDASGFGGGTIY<br>YPTDTSQGTFGAVAISPGFTAGQSSIAWLGPRIASQGFVVITIDTIS<br>RLDQPDSRGRQLLAALDYLTTDSTVRDRIDPNRMAVMGHSMGG<br>GGALSAAAANNPSLKAAIPLQPWHTRKDWSSVRVPTLIVGAQLDTI	E6Q Q34D D36S E68S T91S Q104L H109Y R111T N113D V115T N118D T145P E148K G155P N161D T167V V171I	24	-27.2 kcal/mol

	APVSSHAEAFYNSLPSSLPKAYMELRGADHLVSNTPNTTTAKYSI<br>AWLKRFVDDDTRYEQFLCPAPDDPAISEYRSTCPF	S186A D196S D198P S208D<br>D216N L235T F248P		

Supplementary Table 2. DNA and amino acid sequences of studied enzymes.

Name	DNA Sequence	Amino Acid Sequence
**PHL7**	ATGGCGAACCCGTACGAGCGCGGGCCCGATCCCACCGAGTCGAGCATCGAGGCCGTCCGCGGGCCG<br>TTCGCCGTGGCCCAGACGACGGTGTCGAGGCTCCAGGCCGACGGCTTCGGCGGCGGGACCATCTACT<br>ACCCGACCGACACGAGCCAGGGCACCTTCGGTGCGGTGGCGATCTCGCCGGGGTTCACGGCGGGCC<br>AGGAGAGCATCGCCTGGCTCGGCCCCCGCATCGCGTCGCAGGGCTTCGTGGTGATCACGATCGACAC<br>GATCACGCGCCTCGACCAGCCCGACAGCCGGGGTCGCCAGCTGCAGGCCGCGCTCGACCACCTGCG<br>CACCAACAGCGTCGTGCGCAACCGGATCGACCCGAACCGGATGGCGGTCATGGGCCACTCGATGGGC<br>GGCGGCGGGGCGCTGTCCGCCGCGGCGAACAACACGAGCCTCGAGGCCGCCATCCCGCTGCAGGGC<br>TGGCACACCCGGAAGAACTGGTCGAGCGTGCGGACGCCGACCCTGGTGGTCGGGGCCCAGCTCGAC<br>ACCATCGCGCCGGTGAGCTCGCACTCGGAGGCCTTCTACAACAGCCTGCCGAGCGACCTCGACAAGG<br>CGTACATGGAGCTCCGCGGGGCCAGCCACCTCGTGTCGAACACGCCCGACACGACGACCGCGAAGTA<br>CAGCATCGCCTGGCTCAAGCGGTTCGTCGACGACGACCTCCGCTACGAGCAGTTCCTGTGCCCGGCG<br>CCGGACGACTTCGCGATCTCCGAGTACCGCTCCACCTGCCCGTTCCTCGAGCACCACCACCACCACCA<br>CTGA	MANPYERGPDPTESSIEAVRGP<br>FAVAQTTVSRLQADGFGGGTIY<br>YPTDTSQGTFGAVAISPGFTAG<br>QESIAWLGPRIASQGFVVITIDTI<br>TRLDQPDSRGRQLQAALDHLR<br>TNSVVRNRIDPNRMAVMGHSM<br>GGGGALSAAANNTSLEAAIPLQ<br>GWHTRKNWSSVRTPTLVVGAQ<br>LDTIAPVSSHSEAFYNSLPSDLD<br>KAYMELRGASHLVSNTPDTTTA<br>KYSIAWLKRFVDDDLRYEQFLC<br>PAPDDFAISEYRSTCPF
**PHL7-L93F**	ATGGCGAACCCGTACGAGCGCGGGCCCGATCCCACCGAGTCGAGCATCGAGGCCGTCCGCGGGCCG<br>TTCGCCGTGGCCCAGACGACGGTGTCGAGGCTCCAGGCCGACGGCTTCGGCGGCGGGACCATCTACT<br>ACCCGACCGACACGAGCCAGGGCACCTTCGGTGCGGTGGCGATCTCGCCGGGGTTCACGGCGGGCC<br>AGGAGAGCATCGCCTGGCTCGGCCCCCGCATCGCGTCGCAGGGCTTCGTGGTGATCACGATCGACAC<br>GATCACGCGCTTTGACCAGCCCGACAGCCGGGGTCGCCAGCTGCAGGCCGCGCTCGACCACCTGCGC<br>ACCAACAGCGTCGTGCGCAACCGGATCGACCCGAACCGGATGGCGGTCATGGGCCACTCGATGGGCG<br>GCGGCGGGGCGCTGTCCGCCGCGGCGAACAACACGAGCCTCGAGGCCGCCATCCCGCTGCAGGGCT<br>GGCACACCCGGAAGAACTGGTCGAGCGTGCGGACGCCGACCCTGGTGGTCGGGGCCCAGCTCGACA<br>CCATCGCGCCGGTGAGCTCGCACTCGGAGGCCTTCTACAACAGCCTGCCGAGCGACCTCGACAAGGC<br>GTACATGGAGCTCCGCGGGGCCAGCCACCTCGTGTCGAACACGCCCGACACGACGACCGCGAAGTAC<br>AGCATCGCCTGGCTCAAGCGGTTCGTCGACGACGACCTCCGCTACGAGCAGTTCCTGTGCCCGGCGC<br>CGGACGACTTCGCGATCTCCGAGTACCGCTCCACCTGCCCGTTCCTCGAGCACCACCACCACCACCAC<br>TGA	MANPYERGPDPTESSIEAVRGP<br>FAVAQTTVSRLQADGFGGGTIY<br>YPTDTSQGTFGAVAISPGFTAG<br>QESIAWLGPRIASQGFVVITIDTI<br>TRFDQPDSRGRQLQAALDHLR<br>TNSVVRNRIDPNRMAVMGHSM<br>GGGGALSAAANNTSLEAAIPLQ<br>GWHTRKNWSSVRTPTLVVGAQ<br>LDTIAPVSSHSEAFYNSLPSDLD<br>KAYMELRGASHLVSNTPDTTTA<br>KYSIAWLKRFVDDDLRYEQFLC<br>PAPDDFAISEYRSTCPF
**PHL7-Q95G**	ATGGCGAACCCGTACGAGCGCGGGCCCGATCCCACCGAGTCGAGCATCGAGGCCGTCCGCGGGCCG<br>TTCGCCGTGGCCCAGACGACGGTGTCGAGGCTCCAGGCCGACGGCTTCGGCGGCGGGACCATCTACT<br>ACCCGACCGACACGAGCCAGGGCACCTTCGGTGCGGTGGCGATCTCGCCGGGGTTCACGGCGGGCC<br>AGGAGAGCATCGCCTGGCTCGGCCCCCGCATCGCGTCGCAGGGCTTCGTGGTGATCACGATCGACAC<br>GATCACGCGCCTCGACGGCCCCGACAGCCGGGGTCGCCAGCTGCAGGCCGCGCTCGACCACCTGCG<br>CACCAACAGCGTCGTGCGCAACCGGATCGACCCGAACCGGATGGCGGTCATGGGCCACTCGATGGGC<br>GGCGGCGGGGCGCTGTCCGCCGCGGCGAACAACACGAGCCTCGAGGCCGCCATCCCGCTGCAGGGC<br>TGGCACACCCGGAAGAACTGGTCGAGCGTGCGGACGCCGACCCTGGTGGTCGGGGCCCAGCTCGAC<br>ACCATCGCGCCGGTGAGCTCGCACTCGGAGGCCTTCTACAACAGCCTGCCGAGCGACCTCGACAAGG<br>CGTACATGGAGCTCCGCGGGGCCAGCCACCTCGTGTCGAACACGCCCGACACGACGACCGCGAAGTA<br>CAGCATCGCCTGGCTCAAGCGGTTCGTCGACGACGACCTCCGCTACGAGCAGTTCCTGTGCCCGGCG<br>CCGGACGACTTCGCGATCTCCGAGTACCGCTCCACCTGCCCGTTCCTCGAGCACCACCACCACCACCA<br>CTGA	MANPYERGPDPTESSIEAVRGP<br>FAVAQTTVSRLQADGFGGGTIY<br>YPTDTSQGTFGAVAISPGFTAG<br>QESIAWLGPRIASQGFVVITIDTI<br>TRLDGPDSRGRQLQAALDHLR<br>TNSVVRNRIDPNRMAVMGHSM<br>GGGGALSAAANNTSLEAAIPLQ<br>GWHTRKNWSSVRTPTLVVGAQ<br>LDTIAPVSSHSEAFYNSLPSDLD<br>KAYMELRGASHLVSNTPDTTTA<br>KYSIAWLKRFVDDDLRYEQFLC<br>PAPDDFAISEYRSTCPF
**PHL7-L210T**	ATGGCGAACCCGTACGAGCGCGGGCCCGATCCCACCGAGTCGAGCATCGAGGCCGTCCGCGGGCCG<br>TTCGCCGTGGCCCAGACGACGGTGTCGAGGCTCCAGGCCGACGGCTTCGGCGGCGGGACCATCTACT<br>ACCCGACCGACACGAGCCAGGGCACCTTCGGTGCGGTGGCGATCTCGCCGGGGTTCACGGCGGGCC<br>AGGAGAGCATCGCCTGGCTCGGCCCCCGCATCGCGTCGCAGGGCTTCGTGGTGATCACGATCGACAC<br>GATCACGCGCCTCGACCAGCCCGACAGCCGGGGTCGCCAGCTGCAGGCCGCGCTCGACCACCTGCG<br>CACCAACAGCGTCGTGCGCAACCGGATCGACCCGAACCGGATGGCGGTCATGGGCCACTCGATGGGC<br>GGCGGCGGGGCGCTGTCCGCCGCGGCGAACAACACGAGCCTCGAGGCCGCCATCCCGCTGCAGGGC<br>TGGCACACCCGGAAGAACTGGTCGAGCGTGCGGACGCCGACCCTGGTGGTCGGGGCCCAGCTCGAC<br>ACCATCGCGCCGGTGAGCTCGCACTCGGAGGCCTTCTACAACAGCCTGCCGAGCGACCTCGACAAGG<br>CGTACATGGAGCTCCGCGGGGCCAGCCACACCGTGTCGAACACGCCCGACACGACGACCGCGAAGTA<br>CAGCATCGCCTGGCTCAAGCGGTTCGTCGACGACGACCTCCGCTACGAGCAGTTCCTGTGCCCGGCG<br>CCGGACGACTTCGCGATCTCCGAGTACCGCTCCACCTGCCCGTTCCTCGAGCACCACCACCACCACCA<br>CTGA	MANPYERGPDPTESSIEAVRGP<br>FAVAQTTVSRLQADGFGGGTIY<br>YPTDTSQGTFGAVAISPGFTAG<br>QESIAWLGPRIASQGFVVITIDTI<br>TRLDQPDSRGRQLQAALDHLR<br>TNSVVRNRIDPNRMAVMGHSM<br>GGGGALSAAANNTSLEAAIPLQ<br>GWHTRKNWSSVRTPTLVVGAQ<br>LDTIAPVSSHSEAFYNSLPSDLD<br>KAYMELRGASHTVSNTPDTTTA<br>KYSIAWLKRFVDDDLRYEQFLC<br>PAPDDFAISEYRSTCPF
**PHL7-Q175E**	ATGGCGAACCCGTACGAGCGCGGGCCCGATCCCACCGAGTCGAGCATCGAGGCCGTCCGCGGGCCG<br>TTCGCCGTGGCCCAGACGACGGTGTCGAGGCTCCAGGCCGACGGCTTCGGCGGCGGGACCATCTACT<br>ACCCGACCGACACGAGCCAGGGCACCTTCGGTGCGGTGGCGATCTCGCCGGGGTTCACGGCGGGCC<br>AGGAGAGCATCGCCTGGCTCGGCCCCCGCATCGCGTCGCAGGGCTTCGTGGTGATCACGATCGACAC<br>GATCACGCGCCTCGACCAGCCCGACAGCCGGGGTCGCCAGCTGCAGGCCGCGCTCGACCACCTGCG<br>CACCAACAGCGTCGTGCGCAACCGGATCGACCCGAACCGGATGGCGGTCATGGGCCACTCGATGGGC<br>GGCGGCGGGGCGCTGTCCGCCGCGGCGAACAACACGAGCCTCGAGGCCGCCATCCCGCTGCAGGGC<br>TGGCACACCCGGAAGAACTGGTCGAGCGTGCGGACGCCGACCCTGGTGGTCGGGGCCGAACTCGACA<br>CCATCGCGCCGGTGAGCTCGCACTCGGAGGCCTTCTACAACAGCCTGCCGAGCGACCTCGACAAGGC<br>GTACATGGAGCTCCGCGGGGCCAGCCACCTCGTGTCGAACACGCCCGACACGACGACCGCGAAGTAC<br>AGCATCGCCTGGCTCAAGCGGTTCGTCGACGACGACCTCCGCTACGAGCAGTTCCTGTGCCCGGCGC<br>CGGACGACTTCGCGATCTCCGAGTACCGCTCCACCTGCCCGTTCCTCGAGCACCACCACCACCACCAC<br>TGA	MANPYERGPDPTESSIEAVRGP<br>FAVAQTTVSRLQADGFGGGTIY<br>YPTDTSQGTFGAVAISPGFTAG<br>QESIAWLGPRIASQGFVVITIDTI<br>TRLDQPDSRGRQLQAALDHLR<br>TNSVVRNRIDPNRMAVMGHSM<br>GGGGALSAAANNTSLEAAIPLQ<br>GWHTRKNWSSVRTPTLVVGAE<br>LDTIAPVSSHSEAFYNSLPSDLD<br>KAYMELRGASHLVSNTPDTTTA<br>KYSIAWLKRFVDDDLRYEQFLC<br>PAPDDFAISEYRSTCPF
**PHL7-D233K**	ATGGCGAACCCGTACGAGCGCGGGCCCGATCCCACCGAGTCGAGCATCGAGGCCGTCCGCGGGCCG<br>TTCGCCGTGGCCCAGACGACGGTGTCGAGGCTCCAGGCCGACGGCTTCGGCGGCGGGACCATCTACT	MANPYERGPDPTESSIEAVRGP<br>FAVAQTTVSRLQADGFGGGTIY

	ACCCGACCGACACGAGCCAGGGCACCTTCGGTGCGGTGGCGATCTCGCCGGGGTTCACGGCGGGCC<br>AGGAGAGCATCGCCTGGCTCGGCCCCCGCATCGCGTCGCAGGGCTTCGTGGTGATCACGATCGACAC<br>GATCACGCGCCTCGACCAGCCCGACAGCCGGGGTCGCCAGCTGCAGGCCGCGCTCGACCACCTGCG<br>CACCAACAGCGTCGTGCGCAACCGGATCGACCCGAACCGGATGGCGGTCATGGGCCACTCGATGGGC<br>GGCGGCGGGGCGCTGTCCGCCGCGGCGAACAACACGAGCCTCGAGGCCGCCATCCCGCTGCAGGGC<br>TGGCACACCCGGAAGAACTGGTCGAGCGTGCGGACGCCGACCCTGGTGGTCGGGGCCCAGCTCGAC<br>ACCATCGCGCCGGTGAGCTCGCACTCGGAGGCCTTCTACAACAGCCTGCCGAGCGACCTCGACAAGG<br>CGTACATGGAGCTCCGCGGGGCCAGCCACCTCGTGTCGAACACGCCCGACACGACGACCGCGAAGTA<br>CAGCATCGCCTGGCTCAAGCGGTTCGTCGACAAAGACCTCCGCTACGAGCAGTTCCTGTGCCCGGCGC<br>CGGACGACTTCGCGATCTCCGAGTACCGCTCCACCTGCCCGTTCCTCGAGCACCACCACCACCACCAC<br>TGA	YPTDTSQGTFGAVAISPGFTAG<br>QESIAWLGPRIASQGFVVITIDTI<br>TRLDQPDSRGRQLQAALDHLR<br>TNSVVRNRIDPNRMAVMGHSM<br>GGGGALSAAANNTSLEAAIPLQ<br>GWHTRKNWSSVRTPTLVVGAQ<br>LDTIAPVSSHSEAFYNSLPSDLD<br>KAYMELRGASHLVSNTPDTTTA<br>KYSIAWLKRFVDKDLRYEQFLC<br>PAPDDFAISEYRSTCPF
**PHL7-<br>Q95G/L210<br>T**	ATGGCGAACCCGTACGAGCGCGGGCCCGATCCCACCGAGTCGAGCATCGAGGCCGTCCGCGGGCCG<br>TTCGCCGTGGCCCAGACGACGGTGTCGAGGCTCCAGGCCGACGGCTTCGGCGGCGGGACCATCTACT<br>ACCCGACCGACACGAGCCAGGGCACCTTCGGTGCGGTGGCGATCTCGCCGGGGTTCACGGCGGGCC<br>AGGAGAGCATCGCCTGGCTCGGCCCCCGCATCGCGTCGCAGGGCTTCGTGGTGATCACGATCGACAC<br>GATCACGCGCCTCGACGGCCCCGACAGCCGGGGTCGCCAGCTGCAGGCCGCGCTCGACCACCTGCG<br>CACCAACAGCGTCGTGCGCAACCGGATCGACCCGAACCGGATGGCGGTCATGGGCCACTCGATGGGC<br>GGCGGCGGGGCGCTGTCCGCCGCGGCGAACAACACGAGCCTCGAGGCCGCCATCCCGCTGCAGGGC<br>TGGCACACCCGGAAGAACTGGTCGAGCGTGCGGACGCCGACCCTGGTGGTCGGGGCCCAGCTCGAC<br>ACCATCGCGCCGGTGAGCTCGCACTCGGAGGCCTTCTACAACAGCCTGCCGAGCGACCTCGACAAGG<br>CGTACATGGAGCTCCGCGGGGCCAGCCACACCGTGTCGAACACGCCCGACACGACGACCGCGAAGTA<br>CAGCATCGCCTGGCTCAAGCGGTTCGTCGACGACGACCTCCGCTACGAGCAGTTCCTGTGCCCGGCG<br>CCGGACGACTTCGCGATCTCCGAGTACCGCTCCACCTGCCCGTTCCTCGAGCACCACCACCACCACCA<br>CTGA	MANPYERGPDPTESSIEAVRGP<br>FAVAQTTVSRLQADGFGGGTIY<br>YPTDTSQGTFGAVAISPGFTAG<br>QESIAWLGPRIASQGFVVITIDTI<br>TRLDGPDSRGRQLQAALDHLR<br>TNSVVRNRIDPNRMAVMGHSM<br>GGGGALSAAANNTSLEAAIPLQ<br>GWHTRKNWSSVRTPTLVVGAQ<br>LDTIAPVSSHSEAFYNSLPSDLD<br>KAYMELRGASHTVSNTPDTTTA<br>KYSIAWLKRFVDDDLRYEQFLC<br>PAPDDFAISEYRSTCPF
**PHL7-<br>L93F+Q95Y**	ATGGCGAACCCGTACGAGCGCGGGCCCGATCCCACCGAGTCGAGCATCGAGGCCGTCCGCGGGCCG<br>TTCGCCGTGGCCCAGACGACGGTGTCGAGGCTCCAGGCCGACGGCTTCGGCGGCGGGACCATCTACT<br>ACCCGACCGACACGAGCCAGGGCACCTTCGGTGCGGTGGCGATCTCGCCGGGGTTCACGGCGGGCC<br>AGGAGAGCATCGCCTGGCTCGGCCCCCGCATCGCGTCGCAGGGCTTCGTGGTGATCACGATCGACAC<br>GATCACGCGCTTTGACTATCCCGACAGCCGGGGTCGCCAGCTGCAGGCCGCGCTCGACCACCTGCGC<br>ACCAACAGCGTCGTGCGCAACCGGATCGACCCGAACCGGATGGCGGTCATGGGCCACTCGATGGGCG<br>GCGGCGGGGCGCTGTCCGCCGCGGCGAACAACACGAGCCTCGAGGCCGCCATCCCGCTGCAGGGCT<br>GGCACACCCGGAAGAACTGGTCGAGCGTGCGGACGCCGACCCTGGTGGTCGGGGCCCAGCTCGACA<br>CCATCGCGCCGGTGAGCTCGCACTCGGAGGCCTTCTACAACAGCCTGCCGAGCGACCTCGACAAGGC<br>GTACATGGAGCTCCGCGGGGCCAGCCACACCGTGTCGAACACGCCCGACACGACGACCGCGAAGTAC<br>AGCATCGCCTGGCTCAAGCGGTTCGTCGACGACGACCTCCGCTACGAGCAGTTCCTGTGCCCGGCGC<br>CGGACGACTTCGCGATCTCCGAGTACCGCTCCACCTGCCCGTTCCTCGAGCACCACCACCACCACCAC<br>TGA	MANPYERGPDPTESSIEAVRGP<br>FAVAQTTVSRLQADGFGGGTIY<br>YPTDTSQGTFGAVAISPGFTAG<br>QESIAWLGPRIASQGFVVITIDTI<br>TRFDYPDSRGRQLQAALDHLRT<br>NSVVRNRIDPNRMAVMGHSMG<br>GGGALSAAANNTSLEAAIPLQG<br>WHTRKNWSSVRTPTLVVGAQL<br>DTIAPVSSHSEAFYNSLPSDLDK<br>AYMELRGASHLVSNTPDTTTAK<br>YSIAWLKRFVDDDLRYEQFLCP<br>APDDFAISEYRSTCPF
**PHL7-<br>L210T+Q17<br>5E**	ATGGCGAACCCGTACGAGCGCGGGCCCGATCCCACCGAGTCGAGCATCGAGGCCGTCCGCGGGCCG<br>TTCGCCGTGGCCCAGACGACGGTGTCGAGGCTCCAGGCCGACGGCTTCGGCGGCGGGACCATCTACT<br>ACCCGACCGACACGAGCCAGGGCACCTTCGGTGCGGTGGCGATCTCGCCGGGGTTCACGGCGGGCC<br>AGGAGAGCATCGCCTGGCTCGGCCCCCGCATCGCGTCGCAGGGCTTCGTGGTGATCACGATCGACAC<br>GATCACGCGCCTCGACCAGCCCGACAGCCGGGGTCGCCAGCTGCAGGCCGCGCTCGACCACCTGCG<br>CACCAACAGCGTCGTGCGCAACCGGATCGACCCGAACCGGATGGCGGTCATGGGCCACTCGATGGGC<br>GGCGGCGGGGCGCTGTCCGCCGCGGCGAACAACACGAGCCTCGAGGCCGCCATCCCGCTGCAGGGC<br>TGGCACACCCGGAAGAACTGGTCGAGCGTGCGGACGCCGACCCTGGTGGTCGGGGCCGAACTCGACA<br>CCATCGCGCCGGTGAGCTCGCACTCGGAGGCCTTCTACAACAGCCTGCCGAGCGACCTCGACAAGGC<br>GTACATGGAGCTCCGCGGGGCCAGCCACACCGTGTCGAACACGCCCGACACGACGACCGCGAAGTAC<br>AGCATCGCCTGGCTCAAGCGGTTCGTCGACGACGACCTCCGCTACGAGCAGTTCCTGTGCCCGGCGC<br>CGGACGACTTCGCGATCTCCGAGTACCGCTCCACCTGCCCGTTCCTCGAGCACCACCACCACCACCAC<br>TGA	MANPYERGPDPTESSIEAVRGP<br>FAVAQTTVSRLQADGFGGGTIY<br>YPTDTSQGTFGAVAISPGFTAG<br>QESIAWLGPRIASQGFVVITIDTI<br>TRLDQPDSRGRQLQAALDHLR<br>TNSVVRNRIDPNRMAVMGHSM<br>GGGGALSAAANNTSLEAAIPLQ<br>GWHTRKNWSSVRTPTLVVGAE<br>LDTIAPVSSHSEAFYNSLPSDLD<br>KAYMELRGASHTVSNTPDTTTA<br>KYSIAWLKRFVDDDLRYEQFLC<br>PAPDDFAISEYRSTCPF
**PHL7-<br>L210T+Q17<br>5E+D233K**	ATGGCGAACCCGTACGAGCGCGGGCCCGATCCCACCGAGTCGAGCATCGAGGCCGTCCGCGGGCCG<br>TTCGCCGTGGCCCAGACGACGGTGTCGAGGCTCCAGGCCGACGGCTTCGGCGGCGGGACCATCTACT<br>ACCCGACCGACACGAGCCAGGGCACCTTCGGTGCGGTGGCGATCTCGCCGGGGTTCACGGCGGGCC<br>AGGAGAGCATCGCCTGGCTCGGCCCCCGCATCGCGTCGCAGGGCTTCGTGGTGATCACGATCGACAC<br>GATCACGCGCCTCGACCAGCCCGACAGCCGGGGTCGCCAGCTGCAGGCCGCGCTCGACCACCTGCG<br>CACCAACAGCGTCGTGCGCAACCGGATCGACCCGAACCGGATGGCGGTCATGGGCCACTCGATGGGC<br>GGCGGCGGGGCGCTGTCCGCCGCGGCGAACAACACGAGCCTCGAGGCCGCCATCCCGCTGCAGGGC<br>TGGCACACCCGGAAGAACTGGTCGAGCGTGCGGACGCCGACCCTGGTGGTCGGGGCCGAACTCGACA<br>CCATCGCGCCGGTGAGCTCGCACTCGGAGGCCTTCTACAACAGCCTGCCGAGCGACCTCGACAAGGC<br>GTACATGGAGCTCCGCGGGGCCAGCCACACCGTGTCGAACACGCCCGACACGACGACCGCGAAGTAC<br>AGCATCGCCTGGCTCAAGCGGTTCGTCGACGACGACCTCCGCTACGAGCAGTTCCTGTGCCCGGCGC<br>CGGACGACTTCGCGATCTCCGAGTACCGCTCCACCTGCCCGTTCCTCGAGCACCACCACCACCACCAC<br>TGA	MANPYERGPDPTESSIEAVRGP<br>FAVAQTTVSRLQADGFGGGTIY<br>YPTDTSQGTFGAVAISPGFTAG<br>QESIAWLGPRIASQGFVVITIDTI<br>TRLDQPDSRGRQLQAALDHLR<br>TNSVVRNRIDPNRMAVMGHSM<br>GGGGALSAAANNTSLEAAIPLQ<br>GWHTRKNWSSVRTPTLVVGAE<br>LDTIAPVSSHSEAFYNSLPSDLD<br>KAYMELRGASHTVSNTPDTTTA<br>KYSIAWLKRFVDKDLRYEQFLC<br>PAPDDFAISEYRSTCPF
**PHL7-<br>L93F+Q95G<br>+L210T**	ATGGCGAACCCGTACGAGCGCGGGCCCGATCCCACCGAGTCGAGCATCGAGGCCGTCCGCGGGCCG<br>TTCGCCGTGGCCCAGACGACGGTGTCGAGGCTCCAGGCCGACGGCTTCGGCGGCGGGACCATCTACT<br>ACCCGACCGACACGAGCCAGGGCACCTTCGGTGCGGTGGCGATCTCGCCGGGGTTCACGGCGGGCC<br>AGGAGAGCATCGCCTGGCTCGGCCCCCGCATCGCGTCGCAGGGCTTCGTGGTGATCACGATCGACAC<br>GATCACGCGCTTTGACGGCCCCGACAGCCGGGGTCGCCAGCTGCAGGCCGCGCTCGACCACCTGCG<br>CACCAACAGCGTCGTGCGCAACCGGATCGACCCGAACCGGATGGCGGTCATGGGCCACTCGATGGGC<br>GGCGGCGGGGCGCTGTCCGCCGCGGCGAACAACACGAGCCTCGAGGCCGCCATCCCGCTGCAGGGC<br>TGGCACACCCGGAAGAACTGGTCGAGCGTGCGGACGCCGACCCTGGTGGTCGGGGCCCAGCTCGAC<br>ACCATCGCGCCGGTGAGCTCGCACTCGGAGGCCTTCTACAACAGCCTGCCGAGCGACCTCGACAAGG<br>CGTACATGGAGCTCCGCGGGGCCAGCCACACCGTGTCGAACACGCCCGACACGACGACCGCGAAGTA<br>CAGCATCGCCTGGCTCAAGCGGTTCGTCGACGACGACCTCCGCTACGAGCAGTTCCTGTGCCCGGCG<br>CCGGACGACTTCGCGATCTCCGAGTACCGCTCCACCTGCCCGTTCCTCGAGCACCACCACCACCACCA<br>CTGA	MANPYERGPDPTESSIEAVRGP<br>FAVAQTTVSRLQADGFGGGTIY<br>YPTDTSQGTFGAVAISPGFTAG<br>QESIAWLGPRIASQGFVVITIDTI<br>TRFDGPDSRGRQLQAALDHLR<br>TNSVVRNRIDPNRMAVMGHSM<br>GGGGALSAAANNTSLEAAIPLQ<br>GWHTRKNWSSVRTPTLVVGAQ<br>LDTIAPVSSHSEAFYNSLPSDLD<br>KAYMELRGASHTVSNTPDTTTA<br>KYSIAWLKRFVDDDLRYEQFLC<br>PAPDDFAISEYRSTCPF
**PHL7-<br>Q95G+L210<br>T+D233K**	ATGGCGAACCCGTACGAGCGCGGGCCCGATCCCACCGAGTCGAGCATCGAGGCCGTCCGCGGGCCG<br>TTCGCCGTGGCCCAGACGACGGTGTCGAGGCTCCAGGCCGACGGCTTCGGCGGCGGGACCATCTACT<br>ACCCGACCGACACGAGCCAGGGCACCTTCGGTGCGGTGGCGATCTCGCCGGGGTTCACGGCGGGCC	MANPYERGPDPTESSIEAVRGP<br>FAVAQTTVSRLQADGFGGGTIY<br>YPTDTSQGTFGAVAISPGFTAG

	AGGAGAGCATCGCCTGGCTCGGCCCCCGCATCGCGTCGCAGGGCTTCGTGGTGATCACGATCGACAC<br>GATCACGCGCCTCGACGGCCCCGACAGCCGGGGTCGCCAGCTGCAGGCCGCGCTCGACCACCTGCG<br>CACCAACAGCGTCGTGCGCAACCGGATCGACCCGAACCGGATGGCGGTCATGGGCCACTCGATGGGC<br>GGCGGCGGGGCGCTGTCCGCCGCGGCGAACAACACGAGCCTCGAGGCCGCCATCCCGCTGCAGGGC<br>TGGCACACCCGGAAGAACTGGTCGAGCGTGCGGACGCCGACCCTGGTGGTCGGGGCCCAGCTCGAC<br>ACCATCGCGCCGGTGAGCTCGCACTCGGAGGCCTTCTACAACAGCCTGCCGAGCGACCTCGACAAGG<br>CGTACATGGAGCTCCGCGGGGCCAGCCACACCGTGTCGAACACGCCCGACACGACGACCGCGAAGTA<br>CAGCATCGCCTGGCTCAAGCGGTTCGTCGACAAAGACCTCCGCTACGAGCAGTTCCTGTGCCCGGCGC<br>CGGACGACTTCGCGATCTCCGAGTACCGCTCCACCTGCCCGTTCCTCGAGCACCACCACCACCACCAC<br>TGA	QESIAWLGPRIASQGFVVITIDTI<br>TRLDGPDSRGRQLQAALDHLR<br>TNSVVRNRIDPNRMAVMGHSM<br>GGGGALSAAANNTSLEAAIPLQ<br>GWHTRKNWSSVRTPTLVVGAQ<br>LDTIAPVSSHSEAFYNSLPSDLD<br>KAYMELRGASHTVSNTPDTTTA<br>KYSIAWLKRFVDKDLRYEQFLC<br>PAPDDFAISEYRSTCPF
**PHL7-L93F+Q95G+L210T+D233K**	ATGGCGAACCCGTACGAGCGCGGGCCCGATCCCACCGAGTCGAGCATCGAGGCCGTCCGCGGGCCG<br>TTCGCCGTGGCCCAGACGACGGTGTCGAGGCTCCAGGCCGACGGCTTCGGCGGCGGGACCATCTACT<br>ACCCGACCGACACGAGCCAGGGCACCTTCGGTGCGGTGGCGATCTCGCCGGGGTTCACGGCGGGCC<br>AGGAGAGCATCGCCTGGCTCGGCCCCCGCATCGCGTCGCAGGGCTTCGTGGTGATCACGATCGACAC<br>GATCACGCGCTTTGACGGCCCCGACAGCCGGGGTCGCCAGCTGCAGGCCGCGCTCGACCACCTGCG<br>CACCAACAGCGTCGTGCGCAACCGGATCGACCCGAACCGGATGGCGGTCATGGGCCACTCGATGGGC<br>GGCGGCGGGGCGCTGTCCGCCGCGGCGAACAACACGAGCCTCGAGGCCGCCATCCCGCTGCAGGGC<br>TGGCACACCCGGAAGAACTGGTCGAGCGTGCGGACGCCGACCCTGGTGGTCGGGGCCCAGCTCGAC<br>ACCATCGCGCCGGTGAGCTCGCACTCGGAGGCCTTCTACAACAGCCTGCCGAGCGACCTCGACAAGG<br>CGTACATGGAGCTCCGCGGGGCCAGCCACACCGTGTCGAACACGCCCGACACGACGACCGCGAAGTA<br>CAGCATCGCCTGGCTCAAGCGGTTCGTCGACAAAGACCTCCGCTACGAGCAGTTCCTGTGCCCGGCGC<br>CGGACGACTTCGCGATCTCCGAGTACCGCTCCACCTGCCCGTTCCTCGAGCACCACCACCACCACCAC<br>TGA	MANPYERGPDPTESSIEAVRGP<br>FAVAQTTVSRLQADGFGGGTIY<br>YPTDTSQGTFGAVAISPGFTAG<br>QESIAWLGPRIASQGFVVITIDTI<br>TRFDGPDSRGRQLQAALDHLR<br>TNSVVRNRIDPNRMAVMGHSM<br>GGGGALSAAANNTSLEAAIPLQ<br>GWHTRKNWSSVRTPTLVVGAQ<br>LDTIAPVSSHSEAFYNSLPSDLD<br>KAYMELRGASHTVSNTPDTTTA<br>KYSIAWLKRFVDKDLRYEQFLC<br>PAPDDFAISEYRSTCPF
**PHL7-L93F+Q95Y+Q175E+D233K**	ATGGCGAACCCGTACGAGCGCGGGCCCGATCCCACCGAGTCGAGCATCGAGGCCGTCCGCGGGCCG<br>TTCGCCGTGGCCCAGACGACGGTGTCGAGGCTCCAGGCCGACGGCTTCGGCGGCGGGACCATCTACT<br>ACCCGACCGACACGAGCCAGGGCACCTTCGGTGCGGTGGCGATCTCGCCGGGGTTCACGGCGGGCC<br>AGGAGAGCATCGCCTGGCTCGGCCCCCGCATCGCGTCGCAGGGCTTCGTGGTGATCACGATCGACAC<br>GATCACGCGCTTTGACTATCCCGACAGCCGGGGTCGCCAGCTGCAGGCCGCGCTCGACCACCTGCGC<br>ACCAACAGCGTCGTGCGCAACCGGATCGACCCGAACCGGATGGCGGTCATGGGCCACTCGATGGGCG<br>GCGGCGGGGCGCTGTCCGCCGCGGCGAACAACACGAGCCTCGAGGCCGCCATCCCGCTGCAGGGCT<br>GGCACACCCGGAAGAACTGGTCGAGCGTGCGGACGCCGACCCTGGTGGTCGGGGCCGAACTCGACA<br>CCATCGCGCCGGTGAGCTCGCACTCGGAGGCCTTCTACAACAGCCTGCCGAGCGACCTCGACAAGGC<br>GTACATGGAGCTCCGCGGGGCCAGCCACCTCGTGTCGAACACGCCCGACACGACGACCGCGAAGTAC<br>AGCATCGCCTGGCTCAAGCGGTTCGTCGACAAAGACCTCCGCTACGAGCAGTTCCTGTGCCCGGCGCC<br>GGACGACTTCGCGATCTCCGAGTACCGCTCCACCTGCCCGTTCCTCGAGCACCACCACCACCACCACT<br>GA	MANPYERGPDPTESSIEAVRGP<br>FAVAQTTVSRLQADGFGGGTIY<br>YPTDTSQGTFGAVAISPGFTAG<br>QESIAWLGPRIASQGFVVITIDTI<br>TRFDYPDSRGRQLQAALDHLRT<br>NSVVRNRIDPNRMAVMGHSMG<br>GGGALSAAANNTSLEAAIPLQG<br>WHTRKNWSSVRTPTLVVGAEL<br>DTIAPVSSHSEAFYNSLPSDLDK<br>AYMELRGASHLVSNTPDTTTAK<br>YSIAWLKRFVDKDLRYEQFLCP<br>APDDFAISEYRSTCPF
**PHL7-L210T+L93F**	ATGGCGAACCCGTACGAGCGCGGGCCCGATCCCACCGAGTCGAGCATCGAGGCCGTCCGCGGGCCG<br>TTCGCCGTGGCCCAGACGACGGTGTCGAGGCTCCAGGCCGACGGCTTCGGCGGCGGGACCATCTACT<br>ACCCGACCGACACGAGCCAGGGCACCTTCGGTGCGGTGGCGATCTCGCCGGGGTTCACGGCGGGCC<br>AGGAGAGCATCGCCTGGCTCGGCCCCCGCATCGCGTCGCAGGGCTTCGTGGTGATCACGATCGACAC<br>GATCACGCGCTTTGACCAGCCCGACAGCCGGGGTCGCCAGCTGCAGGCCGCGCTCGACCACCTGCGC<br>ACCAACAGCGTCGTGCGCAACCGGATCGACCCGAACCGGATGGCGGTCATGGGCCACTCGATGGGCG<br>GCGGCGGGGCGCTGTCCGCCGCGGCGAACAACACGAGCCTCGAGGCCGCCATCCCGCTGCAGGGCT<br>GGCACACCCGGAAGAACTGGTCGAGCGTGCGGACGCCGACCCTGGTGGTCGGGGCCCAGCTCGACA<br>CCATCGCGCCGGTGAGCTCGCACTCGGAGGCCTTCTACAACAGCCTGCCGAGCGACCTCGACAAGGC<br>GTACATGGAGCTCCGCGGGGCCAGCCACACCGTGTCGAACACGCCCGACACGACGACCGCGAAGTAC<br>AGCATCGCCTGGCTCAAGCGGTTCGTCGACGACGACCTCCGCTACGAGCAGTTCCTGTGCCCGGCGC<br>CGGACGACTTCGCGATCTCCGAGTACCGCTCCACCTGCCCGTTCCTCGAGCACCACCACCACCACCAC<br>TGA	MANPYERGPDPTESSIEAVRGP<br>FAVAQTTVSRLQADGFGGGTIY<br>YPTDTSQGTFGAVAISPGFTAG<br>QESIAWLGPRIASQGFVVITIDTI<br>TRFDQPDSRGRQLQAALDHLR<br>TNSVVRNRIDPNRMAVMGHSM<br>GGGGALSAAANNTSLEAAIPLQ<br>GWHTRKNWSSVRTPTLVVGAQ<br>LDTIAPVSSHSEAFYNSLPSDLD<br>KAYMELRGASHTVSNTPDTTTA<br>KYSIAWLKRFVDDDLRYEQFLC<br>PAPDDFAISEYRSTCPF
**PHL7-L210T+Q95Y**	ATGGCGAACCCGTACGAGCGCGGGCCCGATCCCACCGAGTCGAGCATCGAGGCCGTCCGCGGGCCG<br>TTCGCCGTGGCCCAGACGACGGTGTCGAGGCTCCAGGCCGACGGCTTCGGCGGCGGGACCATCTACT<br>ACCCGACCGACACGAGCCAGGGCACCTTCGGTGCGGTGGCGATCTCGCCGGGGTTCACGGCGGGCC<br>AGGAGAGCATCGCCTGGCTCGGCCCCCGCATCGCGTCGCAGGGCTTCGTGGTGATCACGATCGACAC<br>GATCACGCGCCTCGACTATCCCGACAGCCGGGGTCGCCAGCTGCAGGCCGCGCTCGACCACCTGCGC<br>ACCAACAGCGTCGTGCGCAACCGGATCGACCCGAACCGGATGGCGGTCATGGGCCACTCGATGGGCG<br>GCGGCGGGGCGCTGTCCGCCGCGGCGAACAACACGAGCCTCGAGGCCGCCATCCCGCTGCAGGGCT<br>GGCACACCCGGAAGAACTGGTCGAGCGTGCGGACGCCGACCCTGGTGGTCGGGGCCCAGCTCGACA<br>CCATCGCGCCGGTGAGCTCGCACTCGGAGGCCTTCTACAACAGCCTGCCGAGCGACCTCGACAAGGC<br>GTACATGGAGCTCCGCGGGGCCAGCCACACCGTGTCGAACACGCCCGACACGACGACCGCGAAGTAC<br>AGCATCGCCTGGCTCAAGCGGTTCGTCGACGACGACCTCCGCTACGAGCAGTTCCTGTGCCCGGCGC<br>CGGACGACTTCGCGATCTCCGAGTACCGCTCCACCTGCCCGTTCCTCGAGCACCACCACCACCACCAC<br>TGA	MANPYERGPDPTESSIEAVRGP<br>FAVAQTTVSRLQADGFGGGTIY<br>YPTDTSQGTFGAVAISPGFTAG<br>QESIAWLGPRIASQGFVVITIDTI<br>TRLDYPDSRGRQLQAALDHLRT<br>NSVVRNRIDPNRMAVMGHSMG<br>GGGALSAAANNTSLEAAIPLQG<br>WHTRKNWSSVRTPTLVVGAQL<br>DTIAPVSSHSEAFYNSLPSDLDK<br>AYMELRGASHTVSNTPDTTTAK<br>YSIAWLKRFVDDDLRYEQFLCP<br>APDDFAISEYRSTCPF
**PHL7-L210T+L93F+Q95Y**	ATGGCGAACCCGTACGAGCGCGGGCCCGATCCCACCGAGTCGAGCATCGAGGCCGTCCGCGGGCCG<br>TTCGCCGTGGCCCAGACGACGGTGTCGAGGCTCCAGGCCGACGGCTTCGGCGGCGGGACCATCTACT<br>ACCCGACCGACACGAGCCAGGGCACCTTCGGTGCGGTGGCGATCTCGCCGGGGTTCACGGCGGGCC<br>AGGAGAGCATCGCCTGGCTCGGCCCCCGCATCGCGTCGCAGGGCTTCGTGGTGATCACGATCGACAC<br>GATCACGCGCTTTGACTATCCCGACAGCCGGGGTCGCCAGCTGCAGGCCGCGCTCGACCACCTGCGC<br>ACCAACAGCGTCGTGCGCAACCGGATCGACCCGAACCGGATGGCGGTCATGGGCCACTCGATGGGCG<br>GCGGCGGGGCGCTGTCCGCCGCGGCGAACAACACGAGCCTCGAGGCCGCCATCCCGCTGCAGGGCT<br>GGCACACCCGGAAGAACTGGTCGAGCGTGCGGACGCCGACCCTGGTGGTCGGGGCCCAGCTCGACA<br>CCATCGCGCCGGTGAGCTCGCACTCGGAGGCCTTCTACAACAGCCTGCCGAGCGACCTCGACAAGGC<br>GTACATGGAGCTCCGCGGGGCCAGCCACACCGTGTCGAACACGCCCGACACGACGACCGCGAAGTAC<br>AGCATCGCCTGGCTCAAGCGGTTCGTCGACGACGACCTCCGCTACGAGCAGTTCCTGTGCCCGGCGC<br>CGGACGACTTCGCGATCTCCGAGTACCGCTCCACCTGCCCGTTCCTCGAGCACCACCACCACCACCAC<br>TGA	MANPYERGPDPTESSIEAVRGP<br>FAVAQTTVSRLQADGFGGGTIY<br>YPTDTSQGTFGAVAISPGFTAG<br>QESIAWLGPRIASQGFVVITIDTI<br>TRFDYPDSRGRQLQAALDHLRT<br>NSVVRNRIDPNRMAVMGHSMG<br>GGGALSAAANNTSLEAAIPLQG<br>WHTRKNWSSVRTPTLVVGAQL<br>DTIAPVSSHSEAFYNSLPSDLDK<br>AYMELRGASHTVSNTPDTTTAK<br>YSIAWLKRFVDDDLRYEQFLCP<br>APDDFAISEYRSTCPF
**PHL7-L93F+Q95G**	ATGGCGAACCCGTACGAGCGCGGGCCCGATCCCACCGAGTCGAGCATCGAGGCCGTCCGCGGGCCG<br>TTCGCCGTGGCCCAGACGACGGTGTCGAGGCTCCAGGCCGACGGCTTCGGCGGCGGGACCATCTACT<br>ACCCGACCGACACGAGCCAGGGCACCTTCGGTGCGGTGGCGATCTCGCCGGGGTTCACGGCGGGCC<br>AGGAGAGCATCGCCTGGCTCGGCCCCCGCATCGCGTCGCAGGGCTTCGTGGTGATCACGATCGACAC	MANPYERGPDPTESSIEAVRGP<br>FAVAQTTVSRLQADGFGGGTIY<br>YPTDTSQGTFGAVAISPGFTAG<br>QESIAWLGPRIASQGFVVITIDTI

	GATCACGCGCTTTGACGGCCCCGACAGCCGGGGTCGCCAGCTGCAGGCCGCGCTCGACCACCTGCG<br>CACCAACAGCGTCGTGCGCAACCGGATCGACCCGAACCGGATGGCGGTCATGGGCCACTCGATGGGC<br>GGCGGCGGGGCGCTGTCCGCCGCGGCGAACAACACGAGCCTCGAGGCCGCCATCCCGCTGCAGGGC<br>TGGCACACCCGGAAGAACTGGTCGAGCGTGCGGACGCCGACCCTGGTGGTCGGGGCCCAGCTCGAC<br>ACCATCGCGCCGGTGAGCTCGCACTCGGAGGCCTTCTACAACAGCCTGCCGAGCGACCTCGACAAGG<br>CGTACATGGAGCTCCGCGGGGCCAGCCACCTCGTGTCGAACACGCCCGACACGACGACCGCGAAGTA<br>CAGCATCGCCTGGCTCAAGCGGTTCGTCGACGACGACCTCCGCTACGAGCAGTTCCTGTGCCCGGCG<br>CCGGACGACTTCGCGATCTCCGAGTACCGCTCCACCTGCCCGTTCCTCGAGCACCACCACCACCACCA<br>CTGA	TRFDGPDSRGRQLQAALDHLR<br>TNSVVRNRIDPNRMAVMGHSM<br>GGGGALSAAANNTSLEAAIPLQ<br>GWHTRKNWSSVRTPTLVVGAQ<br>LDTIAPVSSHSEAFYNSLPSDLD<br>KAYMELRGASHLVSNTPDTTTA<br>KYSIAWLKRFVDDDLRYEQFLC<br>PAPDDFAISEYRSTCPF
**PHL7-L93F+Q95G+L210T+Q175E+D233K**	ATGGCGAACCCGTACGAGCGCGGGCCCGATCCCACCGAGTCGAGCATCGAGGCCGTCCGCGGGCCG<br>TTCGCCGTGGCCCAGACGACGGTGTCGAGGCTCCAGGCCGACGGCTTCGGCGGCGGGACCATCTACT<br>ACCCGACCGACACGAGCCAGGGCACCTTCGGTGCGGTGGCGATCTCGCCGGGGTTCACGGCGGGCC<br>AGGAGAGCATCGCCTGGCTCGGCCCCCGCATCGCGTCGCAGGGCTTCGTGGTGATCACGATCGACAC<br>GATCACGCGCTTTGACGGCCCCGACAGCCGGGGTCGCCAGCTGCAGGCCGCGCTCGACCACCTGCG<br>CACCAACAGCGTCGTGCGCAACCGGATCGACCCGAACCGGATGGCGGTCATGGGCCACTCGATGGGC<br>GGCGGCGGGGCGCTGTCCGCCGCGGCGAACAACACGAGCCTCGAGGCCGCCATCCCGCTGCAGGGC<br>TGGCACACCCGGAAGAACTGGTCGAGCGTGCGGACGCCGACCCTGGTGGTCGGGGCCGAACTCGACA<br>CCATCGCGCCGGTGAGCTCGCACTCGGAGGCCTTCTACAACAGCCTGCCGAGCGACCTCGACAAGGC<br>GTACATGGAGCTCCGCGGGGCCAGCCACACCGTGTCGAACACGCCCGACACGACGACCGCGAAGTAC<br>AGCATCGCCTGGCTCAAGCGGTTCGTCGACAAAGACCTCCGCTACGAGCAGTTCCTGTGCCCGGCGCC<br>GGACGACTTCGCGATCTCCGAGTACCGCTCCACCTGCCCGTTCCTCGAGCACCACCACCACCACCACT<br>GA	MANPYERGPDPTESSIEAVRGP<br>FAVAQTTVSRLQADGFGGGTIY<br>YPTDTSQGTFGAVAISPGFTAG<br>QESIAWLGPRIASQGFVVITIDTI<br>TRFDGPDSRGRQLQAALDHLR<br>TNSVVRNRIDPNRMAVMGHSM<br>GGGGALSAAANNTSLEAAIPLQ<br>GWHTRKNWSSVRTPTLVVGAE<br>LDTIAPVSSHSEAFYNSLPSDLD<br>KAYMELRGASHTVSNTPDTTTA<br>KYSIAWLKRFVDKDLRYEQFLC<br>PAPDDFAISEYRSTCPF
**PHL7-G155P**	ATGGCGAACCCGTACGAGCGCGGGCCCGATCCCACCGAGTCGAGCATCGAGGCCGTCCGCGGGCCG<br>TTCGCCGTGGCCCAGACGACGGTGTCGAGGCTCCAGGCCGACGGCTTCGGCGGCGGGACCATCTACT<br>ACCCGACCGACACGAGCCAGGGCACCTTCGGTGCGGTGGCGATCTCGCCGGGGTTCACGGCGGGCC<br>AGGAGAGCATCGCCTGGCTCGGCCCCCGCATCGCGTCGCAGGGCTTCGTGGTGATCACGATCGACAC<br>GATCACGCGCCTCGACCAGCCCGACAGCCGGGGTCGCCAGCTGCAGGCCGCGCTCGACCACCTGCG<br>CACCAACAGCGTCGTGCGCAACCGGATCGACCCGAACCGGATGGCGGTCATGGGCCACTCGATGGGC<br>GGCGGCGGGGCGCTGTCCGCCGCGGCGAACAACACGAGCCTCGAGGCCGCCATCCCGCTGCAGCCC<br>TGGCACACCCGGAAGAACTGGTCGAGCGTGCGGACGCCGACCCTGGTGGTCGGGGCCCAGCTCGAC<br>ACCATCGCGCCGGTGAGCTCGCACTCGGAGGCCTTCTACAACAGCCTGCCGAGCGACCTCGACAAGG<br>CGTACATGGAGCTCCGCGGGGCCAGCCACCTCGTGTCGAACACGCCCGACACGACGACCGCGAAGTA<br>CAGCATCGCCTGGCTCAAGCGGTTCGTCGACGACGACCTCCGCTACGAGCAGTTCCTGTGCCCGGCG<br>CCGGACGACTTCGCGATCTCCGAGTACCGCTCCACCTGCCCGTTCCTCGAGCACCACCACCACCACCA<br>CTGA	MANPYERGPDPTESSIEAVRGP<br>FAVAQTTVSRLQADGFGGGTIY<br>YPTDTSQGTFGAVAISPGFTAG<br>QESIAWLGPRIASQGFVVITIDTI<br>TRLDQPDSRGRQLQAALDHLR<br>TNSVVRNRIDPNRMAVMGHSM<br>GGGGALSAAANNTSLEAAIPLQ<br>PWHTRKNWSSVRTPTLVVGAQ<br>LDTIAPVSSHSEAFYNSLPSDLD<br>KAYMELRGASHLVSNTPDTTTA<br>KYSIAWLKRFVDDDLRYEQFLC<br>PAPDDFAISEYRSTCPFLEHHH<br>HHH
**PHL7-G155A**	ATGGCGAACCCGTACGAGCGCGGGCCCGATCCCACCGAGTCGAGCATCGAGGCCGTCCGCGGGCCG<br>TTCGCCGTGGCCCAGACGACGGTGTCGAGGCTCCAGGCCGACGGCTTCGGCGGCGGGACCATCTACT<br>ACCCGACCGACACGAGCCAGGGCACCTTCGGTGCGGTGGCGATCTCGCCGGGGTTCACGGCGGGCC<br>AGGAGAGCATCGCCTGGCTCGGCCCCCGCATCGCGTCGCAGGGCTTCGTGGTGATCACGATCGACAC<br>GATCACGCGCCTCGACCAGCCCGACAGCCGGGGTCGCCAGCTGCAGGCCGCGCTCGACCACCTGCG<br>CACCAACAGCGTCGTGCGCAACCGGATCGACCCGAACCGGATGGCGGTCATGGGCCACTCGATGGGC<br>GGCGGCGGGGCGCTGTCCGCCGCGGCGAACAACACGAGCCTCGAGGCCGCCATCCCGCTGCAGGCC<br>TGGCACACCCGGAAGAACTGGTCGAGCGTGCGGACGCCGACCCTGGTGGTCGGGGCCCAGCTCGAC<br>ACCATCGCGCCGGTGAGCTCGCACTCGGAGGCCTTCTACAACAGCCTGCCGAGCGACCTCGACAAGG<br>CGTACATGGAGCTCCGCGGGGCCAGCCACCTCGTGTCGAACACGCCCGACACGACGACCGCGAAGTA<br>CAGCATCGCCTGGCTCAAGCGGTTCGTCGACGACGACCTCCGCTACGAGCAGTTCCTGTGCCCGGCG<br>CCGGACGACTTCGCGATCTCCGAGTACCGCTCCACCTGCCCGTTCCTCGAGCACCACCACCACCACCA<br>CTGA	MANPYERGPDPTESSIEAVRGP<br>FAVAQTTVSRLQADGFGGGTIY<br>YPTDTSQGTFGAVAISPGFTAG<br>QESIAWLGPRIASQGFVVITIDTI<br>TRLDQPDSRGRQLQAALDHLR<br>TNSVVRNRIDPNRMAVMGHSM<br>GGGGALSAAANNTSLEAAIPLQ<br>AWHTRKNWSSVRTPTLVVGAQ<br>LDTIAPVSSHSEAFYNSLPSDLD<br>KAYMELRGASHLVSNTPDTTTA<br>KYSIAWLKRFVDDDLRYEQFLC<br>PAPDDFAISEYRSTCPFLEHHH<br>HHH
**PHL7-G155S**	ATGGCGAACCCGTACGAGCGCGGGCCCGATCCCACCGAGTCGAGCATCGAGGCCGTCCGCGGGCCG<br>TTCGCCGTGGCCCAGACGACGGTGTCGAGGCTCCAGGCCGACGGCTTCGGCGGCGGGACCATCTACT<br>ACCCGACCGACACGAGCCAGGGCACCTTCGGTGCGGTGGCGATCTCGCCGGGGTTCACGGCGGGCC<br>AGGAGAGCATCGCCTGGCTCGGCCCCCGCATCGCGTCGCAGGGCTTCGTGGTGATCACGATCGACAC<br>GATCACGCGCCTCGACCAGCCCGACAGCCGGGGTCGCCAGCTGCAGGCCGCGCTCGACCACCTGCG<br>CACCAACAGCGTCGTGCGCAACCGGATCGACCCGAACCGGATGGCGGTCATGGGCCACTCGATGGGC<br>GGCGGCGGGGCGCTGTCCGCCGCGGCGAACAACACGAGCCTCGAGGCCGCCATCCCGCTGCAGAGC<br>TGGCACACCCGGAAGAACTGGTCGAGCGTGCGGACGCCGACCCTGGTGGTCGGGGCCCAGCTCGAC<br>ACCATCGCGCCGGTGAGCTCGCACTCGGAGGCCTTCTACAACAGCCTGCCGAGCGACCTCGACAAGG<br>CGTACATGGAGCTCCGCGGGGCCAGCCACCTCGTGTCGAACACGCCCGACACGACGACCGCGAAGTA<br>CAGCATCGCCTGGCTCAAGCGGTTCGTCGACGACGACCTCCGCTACGAGCAGTTCCTGTGCCCGGCG<br>CCGGACGACTTCGCGATCTCCGAGTACCGCTCCACCTGCCCGTTCCTCGAGCACCACCACCACCACCA<br>CTGA	MANPYERGPDPTESSIEAVRGP<br>FAVAQTTVSRLQADGFGGGTIY<br>YPTDTSQGTFGAVAISPGFTAG<br>QESIAWLGPRIASQGFVVITIDTI<br>TRLDQPDSRGRQLQAALDHLR<br>TNSVVRNRIDPNRMAVMGHSM<br>GGGGALSAAANNTSLEAAIPLQ<br>SWHTRKNWSSVRTPTLVVGAQ<br>LDTIAPVSSHSEAFYNSLPSDLD<br>KAYMELRGASHLVSNTPDTTTA<br>KYSIAWLKRFVDDDLRYEQFLC<br>PAPDDFAISEYRSTCPFLEHHH<br>HHH
**PHL7-G155T**	ATGGCGAACCCGTACGAGCGCGGGCCCGATCCCACCGAGTCGAGCATCGAGGCCGTCCGCGGGCCG<br>TTCGCCGTGGCCCAGACGACGGTGTCGAGGCTCCAGGCCGACGGCTTCGGCGGCGGGACCATCTACT<br>ACCCGACCGACACGAGCCAGGGCACCTTCGGTGCGGTGGCGATCTCGCCGGGGTTCACGGCGGGCC<br>AGGAGAGCATCGCCTGGCTCGGCCCCCGCATCGCGTCGCAGGGCTTCGTGGTGATCACGATCGACAC<br>GATCACGCGCCTCGACCAGCCCGACAGCCGGGGTCGCCAGCTGCAGGCCGCGCTCGACCACCTGCG<br>CACCAACAGCGTCGTGCGCAACCGGATCGACCCGAACCGGATGGCGGTCATGGGCCACTCGATGGGC<br>GGCGGCGGGGCGCTGTCCGCCGCGGCGAACAACACGAGCCTCGAGGCCGCCATCCCGCTGCAGACC<br>TGGCACACCCGGAAGAACTGGTCGAGCGTGCGGACGCCGACCCTGGTGGTCGGGGCCCAGCTCGAC<br>ACCATCGCGCCGGTGAGCTCGCACTCGGAGGCCTTCTACAACAGCCTGCCGAGCGACCTCGACAAGG<br>CGTACATGGAGCTCCGCGGGGCCAGCCACCTCGTGTCGAACACGCCCGACACGACGACCGCGAAGTA<br>CAGCATCGCCTGGCTCAAGCGGTTCGTCGACGACGACCTCCGCTACGAGCAGTTCCTGTGCCCGGCG<br>CCGGACGACTTCGCGATCTCCGAGTACCGCTCCACCTGCCCGTTCCTCGAGCACCACCACCACCACCA<br>CTGA	MANPYERGPDPTESSIEAVRGP<br>FAVAQTTVSRLQADGFGGGTIY<br>YPTDTSQGTFGAVAISPGFTAG<br>QESIAWLGPRIASQGFVVITIDTI<br>TRLDQPDSRGRQLQAALDHLR<br>TNSVVRNRIDPNRMAVMGHSM<br>GGGGALSAAANNTSLEAAIPLQ<br>TWHTRKNWSSVRTPTLVVGAQ<br>LDTIAPVSSHSEAFYNSLPSDLD<br>KAYMELRGASHLVSNTPDTTTA<br>KYSIAWLKRFVDDDLRYEQFLC<br>PAPDDFAISEYRSTCPFLEHHH<br>HHH
**R1M1**	ATGGCGAACCCGTACGAGCGTGGTCCGGACCCGACCGAGAGCAGCATCGAAGCGGTTCGTGGTCCGT<br>TTGCGGTGGCGCAGACCACCGTTAGCCGTCTGCAAGCGGATGGCTTTGGTGGCGGTACCATCTACTAT<br>CCGACCGACACCAGCCAGGGTACCTTTGGTGCGGTTGCGATTAGCCCGGGCTTTACCGCGGGTCAGG<br>AAAGCATTGCGTGGCTGGGTCCGCGTATTGCGAGCCAAGGTTTCGTGGTTATCACCATTGACACCATCA<br>CCCGTCTGGATCAGCCGGACAGCCGTGGCCGTCAACTGCTGGCGGCGCTGGATCACCTGACCACCAA	MANPYERGPDPTESSIEAVRGP<br>FAVAQTTVSRLQADGFGGGTIY<br>YPTDTSQGTFGAVAISPGFTAG<br>QESIAWLGPRIASQGFVVITIDTI<br>TRLDQPDSRGRQLLAALDHLTT

	CAGCGTGGTTCGTAACCGTATTGACCCGAACCGTATGGCGGTGATGGGTCACAGCATGGGCGGTGGC<br>GGTGCGCTGAGCGCGGCGGCGAACAACCCGAGCCTGAAAGCGGCGATCCCGCTGCAGCCGTGGCAC<br>ACCCGTAAAAACTGGAGCAGCGTGCGTGTTCCGACCCTGGTGGTTGGCGCGCAACTGGATACCATTGC<br>GCCGGTTAGCAGCCACAGCGAGGCGTTCTACAACAGCCTGCCGAGCGATCTGGACAAGGCGTATATG<br>GAACTGCGTGGTGCGAGCCACCTGGTTAGCAACACCCCGGACACCACCACCGCGAAGTACAGCATCG<br>CGTGGCTGAAACGTTTCGTTGACGATGACACCCGTTATGAGCAATTTCTGTGCCCGGCGCCGGATGAC<br>CCGGCGATTAGCGAATATCGTAGCACCTGCCCGTTTCTCGAGCACCACCACCACCACCACTGA	NSVVRNRIDPNRMAVMGHSMG<br>GGGALSAAANNPSLKAAIPLQP<br>WHTRKNWSSVRVPTLVVGAQL<br>DTIAPVSSHSEAFYNSLPSDLDK<br>AYMELRGASHLVSNTPDTTTAK<br>YSIAWLKRFVDDDTRYEQFLCP<br>APDDPAISEYRSTCPFLEHHHH<br>HH
**R1M2**	ATGGCGAACCCGTACGAGCGTGGTCCGGACCCGACCGAGAGCAGCATCGAAGCGGTTCGTGGTCCGT<br>TTGCGGTGGCGCAGACCACCGTTAGCCGTCTGCAAGCGGATGGCTTTGGTGGCGGTACCATCTACTAT<br>CCGACCGACACCAGCCAGGGTACCTTTGGTGCGGTTGCGATTAGCCCGGGCTTTACCGCGGGTCAGG<br>AAAGCATTGCGTGGCTGGGTCCGCGTATTGCGAGCCAAGGTTTCGTGGTTATCACCATTGACACCATCA<br>CCCGTCTGGATCAGCCGGACAGCCGTGGCCGTCAACTGCTGGCGGCGCTGGATCACCTGACCACCAA<br>CAGCGTGGTTCGTAACCGTATTGACCCGAACCGTATGGCGGTGATGGGTCACAGCATGGGCGGTGGC<br>GGTGCGCTGAGCGCGGCGGCGAACAACCCGAGCCTGAAAGCGGCGATCCCGCTGCAGCCGTGGCAC<br>ACCCGTAAAAACTGGAGCAGCGTGCGTGTTCCGACCCTGGTGGTTGGCGCGCAAAACGATACCATTGC<br>GCCGGTTAGCAGCCACAGCGAGGCGTTCTACAACAGCCTGCCGAGCGATCTGGACAAGGCGTATATG<br>GAACTGCGTGGTGCGAGCCACCTGGTTAGCAACACCCCGGACACCACCATCGCGAAGTACAGCATTGC<br>GTGGCTGAAACGTTTCGTTGACGATGACACCCGTTATGAGCAATTTCTGTGCCCGGCGCCGGATGACC<br>CGGCGATTAGCGAATATCGTAGCACCTGCCCGTTTCTCGAGCACCACCACCACCACCACTGA	MANPYERGPDPTESSIEAVRGP<br>FAVAQTTVSRLQADGFGGGTIY<br>YPTDTSQGTFGAVAISPGFTAG<br>QESIAWLGPRIASQGFVVITIDTI<br>TRLDQPDSRGRQLLAALDHLTT<br>NSVVRNRIDPNRMAVMGHSMG<br>GGGALSAAANNPSLKAAIPLQP<br>WHTRKNWSSVRVPTLVVGAQN<br>DTIAPVSSHSEAFYNSLPSDLDK<br>AYMELRGASHLVSNTPDTTIAK<br>YSIAWLKRFVDDDTRYEQFLCP<br>APDDPAISEYRSTCPFLEHHHH<br>HH
**R1M3**	ATGGCGAACCCGTACGAGCGTGGTCCGGACCCGACCGAGAGCAGCATCGAAGCGGTTCGTGGTCCGT<br>TTGCGGTGGCGCAGACCACCGTTAGCCGTCTGCAAGCGGATGGCTTTGGTGGCGGTACCATCTACTAT<br>CCGACCGACACCAGCCAGGGTACCTTTGGTGCGGTTGCGATTAGCCCGGGTTTTACCGCGACCCAAGA<br>AAGCATTGCGTGGCTGGGTCCGCGTATTGCGAGCCAAGGTTTCGTGGTTATCACCATTGACACCATCAC<br>CCGTCTGGATCAGCCGGACAGCCGTGGCCGTCAACTGCTGGCGGCGCTGGATCACCTGACCACCAAC<br>AGCGTGGTTCGTAACCGTATTGACCCGAACCGTATGGCGGTGATGGGTCACAGCATGGGCGGTGGCG<br>GTGCGCTGAGCGCGGCGGCGAACAACCCGAGCCTGAAAGCGGCGATCCCGCTGCAGCCGTGGCACA<br>CCCGTAAAAACTGGAGCAGCGTGCGTGTTCCGACCCTGGTGGTTGGCGCGCAAAACGATACCATTGCG<br>CCGGTTAGCAGCCACAGCGAGGCGTTCTACAACAGCCTGCCGAGCGATCTGGACAAGGCGTATATGGA<br>ACTGCGTGGTGCGAGCCACCTGGTGACCAACACCCCGGACACCACCATCGCGAAGTACAGCATTGCGT<br>GGCTGAAACGTTTCGTTGACGATGACACCCGTTATGAGCAATTTCTGTGCCCGGCGCCGGATGACCCG<br>GCGATTAGCGAATATCGTAGCACCTGCCCGTTTCTCGAGCACCACCACCACCACCACTGA	MANPYERGPDPTESSIEAVRGP<br>FAVAQTTVSRLQADGFGGGTIY<br>YPTDTSQGTFGAVAISPGFTAT<br>QESIAWLGPRIASQGFVVITIDTI<br>TRLDQPDSRGRQLLAALDHLTT<br>NSVVRNRIDPNRMAVMGHSMG<br>GGGALSAAANNPSLKAAIPLQP<br>WHTRKNWSSVRVPTLVVGAQN<br>DTIAPVSSHSEAFYNSLPSDLDK<br>AYMELRGASHLVTNTPDTTIAK<br>YSIAWLKRFVDDDTRYEQFLCP<br>APDDPAISEYRSTCPFLEHHHH<br>HH
**R1M4**	ATGGCGAACCCGTACGAGCGTGGTCCGGACCCGACCGAGAGCAGCATCGAAGCGGTTCGTGGTCCGT<br>TTGCGGTGGCGCAGACCACCGTTAGCCGTCTGCAAGCGGATGGCTTTGGTGGCGGTACCATCTACTAT<br>CCGACCGACACCAGCCAGGGTACCTTTGGTGCGGTTGCGATTAGCCCGGGTTTTACCGCGACCCAAAG<br>CAGCATTGCGTGGCTGGGTCCGCGTATTGCGAGCCAAGGTTTCGTGGTTATCACCATTGACACCATCAC<br>CCGTCTGGATCAGCCGGACAGCCGTGGCCGTCAACTGCTGGCGGCGCTGGATTATCTGACCACCAACA<br>GCGTGGTTCGTAACCGTATTGACCCGAACCGTATGGCGGTGATGGGTCACAGCATGGGCGGTGGCGG<br>TGCGCTGAGCGCGGCGGCGAACAACCCGAGCCTGAAAGCGGCGATCCCGCTGCAGCCGTGGCACAC<br>CCGTAAAAACTGGAGCAGCGTGCGTGTTCCGACCCTGGTGGTTGGCGCGCAAAACGATACCATTGCGC<br>CGGTTAGCAGCCATGCGGAGGCGTTCTACAACAGCCTGCCGAGCGATCTGGACAAGGCGTATATGGAA<br>CTGCGTGGTGCGAGCCACCTGGTGACCAACACCCCGGACACCACCATCGCGAAGTACAGCATTGCGT<br>GGCTGAAACGTTTCGTTGACGATGACACCCGTTATGAGCAATTTCTGTGCCCGGCGCCGGATGACCCG<br>GCGATTAGCGAATACCGTAGCACCTGCCCGTTTCTCGAGCACCACCACCACCACCACTGA	MANPYERGPDPTESSIEAVRGP<br>FAVAQTTVSRLQADGFGGGTIY<br>YPTDTSQGTFGAVAISPGFTAT<br>QSSIAWLGPRIASQGFVVITIDTI<br>TRLDQPDSRGRQLLAALDYLTT<br>NSVVRNRIDPNRMAVMGHSMG<br>GGGALSAAANNPSLKAAIPLQP<br>WHTRKNWSSVRVPTLVVGAQN<br>DTIAPVSSHAEAFYNSLPSDLDK<br>AYMELRGASHLVTNTPDTTIAK<br>YSIAWLKRFVDDDTRYEQFLCP<br>APDDPAISEYRSTCPFLEHHHH<br>HH
**R1M5**	ATGGCGAACCCGTACGAGCGTGGTCCGGACCCGACCGAGAGCAGCATTGAAGCGGCGCGTGGTCCGT<br>TTGCGGTGGCGCAGACCACCGTTAGCCGTCTGCAAGCGGATGGCTTTGGTGGCGGTACCATCTACTAT<br>CCGACCGATACCAGCCAGGGTACCTTTGGTGCGGTGGCGATTAGCCCGGGTTTTACCGCGACCCAAAG<br>CAGCATTGCGTGGCTGGGTCCGCGTATTGCGAGCCAAGGTTTCGTGGTTATCACCATTGACACCAACA<br>CCCGTCTGGATCAGCCGGACAGCCGTGGCCGTCAACTGCTGGCGGCGCTGGATTATCTGACCACCAA<br>CAGCGTGGTTCGTAACCGTATCGACCCGAACCGTATGGCGGTTATGGGTCACAGCATGGGCGGTGGC<br>GGTGCGCTGAGCGCGGCGGCGAACAACCCGAGCCTGAAAGCGGCGATTCCGCTGCAGCCGTGGCAC<br>ACCCGTAAAAACTGGAGCAGCGTGCGTGTTCCGACCCTGGTGATCGGCGCGCAAAACGATACCATTGC<br>GCCGGTTAGCAGCCATGCGGAGGCGTTCTACAACAGCCTGCCGAGCGATCTGGACAAGGCGTATATG<br>GAACTGCGTGGTGCGAGCCACCTGGTGACCAACACCCCGGACACCACCATCGCGAAGTACAGCATTGC<br>GTGGCTGAAACGTTTCGTTGACGATGACACCCGTTATGAGCAATTTCTGTGCCCGGCGCCGGATGACC<br>CGGCGATTAGCGAATACCGTAGCACCTGCCCGTTTCTCGAGCACCACCACCACCACCACTGA	MANPYERGPDPTESSIEAARGP<br>FAVAQTTVSRLQADGFGGGTIY<br>YPTDTSQGTFGAVAISPGFTAT<br>QSSIAWLGPRIASQGFVVITIDT<br>NTRLDQPDSRGRQLLAALDYLT<br>TNSVVRNRIDPNRMAVMGHSM<br>GGGGALSAAANNPSLKAAIPLQ<br>PWHTRKNWSSVRVPTLVIGAQ<br>NDTIAPVSSHAEAFYNSLPSDLD<br>KAYMELRGASHLVTNTPDTTIA<br>KYSIAWLKRFVDDDTRYEQFLC<br>PAPDDPAISEYRSTCPFLEHHH<br>HHH
**R1M6**	ATGGCGAACCCGTACGAGCGTGGTCCGGACCCGACCGAGAGCAGCATTGAAGCGGCGCGTGGTCCGT<br>TTGCGGTGGCGCAGACCACCGTTAGCCGTCTGCAAGCGAGCGGCTTTGGTGGCGGTACCATCTACTAT<br>CCGACCGATACCAGCCAGGGTACCTTTGGTGCGGTGGCGATTAGCCCGGGTTTTACCGCGACCCAAAG<br>CAGCATTGCGTGGCTGGGTCCGCGTATTGCGAGCCAAGGTTTCGTGGTTATCACCATTGATACCAACAC<br>CCGTCTGGATCAGCCGGACAGCCGTGGCCGTCAACTGCTGGCGGCGCTGGACTATCTGACCACCAAC<br>AGCACCGTGCGTGATCGTATTGACCCGAACCGTATGGCGGTTATGGGTCACAGCATGGGCGGTGGCG<br>GTGCGCTGAGCGCGGCGGCGAACAACCCGAGCCTGAAAGCGGCGATCCCGCTGCAGCCGTGGCACA<br>CCCGTAAAAACTGGAGCAGCGTGCGTGTTCCGACCCTGATCATTGGCGCGCAAAACGATACCATTGCG<br>CCGGTTAGCAGCCATGCGGAGGCGTTCTACAACAGCCTGCCGAGCGATCTGGACAAGGCGTATATGGA<br>ACTGCGTGGTGCGAGCCACCTGGTGACCAACACCCCGAACACCACCATCGCGAAGTACAGCATTGCGT<br>GGCTGAAACGTTTCGTTGACGATGACACCCGTTATGAGCAATTTCTGTGCCCGGCGCCGGATGACCCG<br>GCGATCAGCGAATACCGTAGCACCTGCCCGTTTCTCGAGCACCACCACCACCACCACTGA	MANPYERGPDPTESSIEAARGP<br>FAVAQTTVSRLQASGFGGGTIY<br>YPTDTSQGTFGAVAISPGFTAT<br>QSSIAWLGPRIASQGFVVITIDT<br>NTRLDQPDSRGRQLLAALDYLT<br>TNSTVRDRIDPNRMAVMGHSM<br>GGGGALSAAANNPSLKAAIPLQ<br>PWHTRKNWSSVRVPTLIIGAQN<br>DTIAPVSSHAEAFYNSLPSDLDK<br>AYMELRGASHLVTNTPNTTIAK<br>YSIAWLKRFVDDDTRYEQFLCP<br>APDDPAISEYRSTCPFLEHHHH<br>HH
**R1M7**	ATGGCGAACCCGTACGAGCGTGGTCCGGACCCGACCGAGAGCAGCATTGAAGCGGCGCGTGGTCCGT<br>TTGCGGTGGCGCAGACCACCGTTAGCCGTCTGCAAGCGAGCGGCTTTGGTGGCGGTACCATCTACTAT<br>CCGACCGATACCAGCCAGGGTACCTTTGGTGCGGTGGCGATTAGCCCGGGTTTTACCGCGACCCAAAG<br>CAGCATTGCGTGGCTGGGTCCGCGTATTGCGAGCCAAGGTTTCGTGGTTATCACCATTGACACCAACA<br>CCCGTCTGGACCAGCCGGATAGCCGTGGCCGTCAACTGCTGGCGGCGCTGGACTATCTGACCACCGA<br>TAGCACCGTGCGTGACCGTATTGATCCGAACCGTATGGCGGTTATGGGTCACAGCATGGGCGGTGGCG	MANPYERGPDPTESSIEAARGP<br>FAVAQTTVSRLQASGFGGGTIY<br>YPTDTSQGTFGAVAISPGFTAT<br>QSSIAWLGPRIASQGFVVITIDT<br>NTRLDQPDSRGRQLLAALDYLT<br>TDSTVRDRIDPNRMAVMGHSM

	GTGCGCTGAGCGCGGCGGCGAACAACCCGAGCCTGAAAGCGGCGATCCCGCTGCAGCCGTGGCACA<br>CCCGTAAAAACTGGAGCAGCGTGCGTGTTCCGACCCTGATCATTGGCGCGCAAAACGACACCATTGCG<br>CCGGTTAGCAGCCATGCGGAGGCGTTCTACAACAGCCTGCCGAGCAGCCTGGATAAGGCGTATATGGA<br>ACTGCGTGGTGCGAGCCACCTGGTGACCAACACCCCGAACACCACCATCGCGAAGTACAGCATTGCGT<br>GGCTGAAACGTTTCGTTGACAACGATACCCGTTATGAGCAATTTCTGTGCCCGGCGCCGGATGATCCG<br>GCGATCAGCGAATACCGTAGCACCTGCCCGTTTCTCGAGCACCACCACCACCACCACTGA	GGGGALSAAANNPSLKAAIPLQ<br>PWHTRKNWSSVRVPTLIIGAQN<br>DTIAPVSSHAEAFYNSLPSSLDK<br>AYMELRGASHLVTNTPNTTIAK<br>YSIAWLKRFVDNDTRYEQFLCP<br>APDDPAISEYRSTCPFLEHHHH<br>HH
**R1M8**	ATGGCGAACCCGTACGAGCGTGGTCCGGACCCGACCGAGGCGAGCATTGAAGCGCCGCGTGGTCCGT<br>TTGCGGTGGCGCAGACCACCGTTAGCCGTCTGCAAGCGCGTGGCTTTGGTGGCGGTACCATTTACTAT<br>CCGACCGATACCAGCCAAGGTACCTTTGGTGCGGTGGCGATCAGCCCGGGTTTTACCGCGACCCAAAG<br>CAGCATTGCGTGGCTGGGTCCGCGTCTGGCGAGCCAAGGTTTCGTGGTTATCACCATTGATACCAACA<br>CCCGTTACGATCAGCCGGACAGCCGTGGCCGTCAACTGCTGGCGGCGCTGGATTATCTGACCACCGA<br>CAGCACCGTGCGTGATCGTATCGACCCGAACCGTATGGCGGTTATGGGTCACAGCATGGGCGGTGGC<br>GGTGCGCTGGCGGCGGCGGCGAACAACCCGAGCCTGAAAGCGGCGATCCCGCTGCAGCCGTGGCAC<br>ACCCGTAAAAACTGGAGCAGCGTGCGTGTTCCGACCCTGATCATTGGCGCGCAGAACGATACCATTGC<br>GCCGGTTAGCCAACACGCGGAGGCGTTCTACAACAGCCTGCCGAGCAGCCTGGACAAGGCGTATATG<br>GAACTGCGTGGTGCGAGCCACCTGGTGACCAACACCCCGAACACCACCATCGCGAAGTACGCGATTGC<br>GTGGCTGAAACGTTTCGTTGACGATGACACCCGTTATGAGCAATTTCTGTGCCCGGCGCCGGATGACC<br>CGGCGATTAGCGAATATCGTAGCACCTGCCCGTTTCTCGAGCACCACCACCACCACCACTGA	MANPYERGPDPTEASIEAPRGP<br>FAVAQTTVSRLQARGFGGGTIY<br>YPTDTSQGTFGAVAISPGFTAT<br>QSSIAWLGPRLASQGFVVITIDT<br>NTRYDQPDSRGRQLLAALDYLT<br>TDSTVRDRIDPNRMAVMGHSM<br>GGGGALAAAANNPSLKAAIPLQ<br>PWHTRKNWSSVRVPTLIIGAQN<br>DTIAPVSQHAEAFYNSLPSSLDK<br>AYMELRGASHLVTNTPNTTIAK<br>YAIAWLKRFVDDDTRYEQFLCP<br>APDDPAISEYRSTCPFLEHHHH<br>HH
**R1M9**	ATGGCGAACCCGTATCAACGTGGTCCGGACCCGACCGAGGCGAGCATTGAAGCGCCGCGTGGTCCGT<br>TTGCGGTGGCGCAAACCACCGTTAGCCGTCTGGATGCGCGTGGCTTTGGTGGCGGTACCATTTACTAT<br>CCGACCGATACCAGCCAAGGTACCTTTGGTGCGGTGGCGATCAGCCCGGGTTATCTGGCGACCCAAAG<br>CAGCATTGCGTGGCTGGGTCCGCGTCTGGCGAGCCAAGGTTTTGTGGTTATCACCATTGACACCAACA<br>GCCGTTACGATCAGCCGGACAGCCGTGGCCGTCAACTGCTGGCGGCGCTGGATTATCTGACCACCGA<br>CAGCACCGTGCGTGATCGTATCGACCCGAACCGTATGGCGGTTATGGGTCACAGCATGGGCGGTGGC<br>GGTGCGCTGGCGGCGGCGGCGAACAACCCGAGCCTGAAAGCGGCGATCCCGCTGCAGCCGTGGCAC<br>ACCCGTAAAGATTGGAGCAGCGTGCGTGTTCCGACCCTGATCATTGGCGCGCAGAACGACACCATTGC<br>GCCGGTTAGCCAACATGCGGAGGCGTTCTACAACAGCCTGCCGAGCAGCCTGCCGAAGGCGTATATG<br>GAACTGCGTGGTGCGGATCACCTGGTTGCGACCACCCCGAACACCACCATCGCGAAGTACGTGATTGC<br>GTGGCTGAAACGTTTCGTTGACGATGACACCCGTTATGAGCAATTTCTGTGCCCGGCGCCGGATGACC<br>CGGCGATTAGCGAATACCGTAGCACCTGCCCGTTTCTCGAGCACCACCACCACCACCACTGA	MANPYQRGPDPTEASIEAPRGP<br>FAVAQTTVSRLDARGFGGGTIY<br>YPTDTSQGTFGAVAISPGYLAT<br>QSSIAWLGPRLASQGFVVITIDT<br>NSRYDQPDSRGRQLLAALDYLT<br>TDSTVRDRIDPNRMAVMGHSM<br>GGGGALAAAANNPSLKAAIPLQ<br>PWHTRKDWSSVRVPTLIIGAQN<br>DTIAPVSQHAEAFYNSLPSSLPK<br>AYMELRGADHLVATTPNTTIAK<br>YVIAWLKRFVDDDTRYEQFLCP<br>APDDPAISEYRSTCPFLEHHHH<br>HH
**R2M1**	ATGGCGAACCCGTACGAGCGTGGTCCGGATCCGACCGAGAGCAGCATCGAAGCGGTTCGTGGTCCGT<br>TTGCGGTGGCGCAGACCACCGTTAGCCGTCTGCAAGCGGATGGCTTTGGTGGCGGTACCATCTACTAT<br>CCGACCGACACCAGCCAGGGTACCTTTGGTGCGGTTGCGATTAGCCCGGGCTTTACCGCGGGTCAGA<br>GCAGCATTGCGTGGCTGGGTCCGCGTATTGCGAGCCAAGGTTTCGTGGTTATCACCATTGATACCATCA<br>CCCGTCTGGATCAGCCGGACAGCCGTGGCCGTCAACTGCTGGCGGCGCTGGATTATCTGACCACCGA<br>CAGCGTGGTTCGTAACCGTATTGACCCGAACCGTATGGCGGTGATGGGTCACAGCATGGGCGGTGGC<br>GGTGCGCTGAGCGCGGCGGCGAACAACCCGAGCCTGAAAGCGGCGATCCCGCTGCAGCCGTGGCAC<br>ACCCGTAAAAACTGGAGCAGCGTGCGTGTTCCGACCCTGGTGGTTGGCGCGCAAAACGACACCATTGC<br>GCCGGTTAGCAGCCATGCGGAGGCGTTCTACAACAGCCTGCCGAGCAGCCTGGATAAGGCGTATATG<br>GAACTGCGTGGTGCGAGCCACCTGGTTAGCAACACCCCGGACACCACCATCGCGAAGTACAGCATTGC<br>GTGGCTGAAACGTTTCGTTGACGATGACACCCGTTATGAGCAATTTCTGTGCCCGGCGCCGGATGACC<br>CGGCGATTAGCGAATACCGTAGCACCTGCCCGTTTCTCGAGCACCACCACCACCACCACTGA	MANPYERGPDPTESSIEAVRGP<br>FAVAQTTVSRLQADGFGGGTIY<br>YPTDTSQGTFGAVAISPGFTAG<br>QSSIAWLGPRIASQGFVVITIDTI<br>TRLDQPDSRGRQLLAALDYLTT<br>DSVVRNRIDPNRMAVMGHSMG<br>GGGALSAAANNPSLKAAIPLQP<br>WHTRKNWSSVRVPTLVVGAQN<br>DTIAPVSSHAEAFYNSLPSSLDK<br>AYMELRGASHLVSNTPDTTIAK<br>YSIAWLKRFVDDDTRYEQFLCP<br>APDDPAISEYRSTCPFLEHHHH<br>HH
**R2M2**	ATGGCGAACCCGTATCAACGTGGTCCGGATCCGACCGAGAGCAGCATTGAAGCGGTGCGTGGTCCGTT<br>TGCGGTGGCGCAAACCACCGTTAGCCGTCTGGATGCGAGCGGCTTTGGTGGCGGTACCATCTACTATC<br>CGACCGACACCAGCCAGGGTACCTTTGGTGCGGTTGCGATTAGCCCGGGCTTTACCGCGGGTCAGAG<br>CAGCATTGCGTGGCTGGGTCCGCGTATTGCGAGCCAAGGTTTCGTGGTTATCACCATTGACACCATCA<br>GCCGTCTGGATCAGCCGGACAGCCGTGGCCGTCAACTGCTGGCGGCGCTGGATTATCTGACCACCGA<br>CAGCACCGTGCGTGATCGTATTGACCCGAACCGTATGGCGGTTATGGGTCACAGCATGGGCGGTGGC<br>GGTGCGCTGAGCGCGGCGGCGAACAACCCGAGCCTGAAAGCGGCGATTCCGCTGCAGCCGTGGCAC<br>ACCCGTAAAGATTGGAGCAGCGTGCGTGTTCCGACCCTGATCGTGGGCGCGCAACTGGACACCATTGC<br>GCCGGTTAGCAGCCATGCGGAGGCGTTCTACAACAGCCTGCCGAGCAGCCTGCCGAAGGCGTATATG<br>GAACTGCGTGGTGCGGATCACCTGGTTAGCAACACCCCGAACACCACCACCGCGAAGTACAGCATCGC<br>GTGGCTGAAACGTTTCGTTGACGATGACACCCGTTATGAGCAATTTCTGTGCCCGGCGCCGGATGACC<br>CGGCGATTAGCGAATACCGTAGCACCTGCCCGTTTCTCGAGCACCACCACCACCACCACTGA	MANPYQRGPDPTESSIEAVRGP<br>FAVAQTTVSRLDASGFGGGTIY<br>YPTDTSQGTFGAVAISPGFTAG<br>QSSIAWLGPRIASQGFVVITIDTI<br>SRLDQPDSRGRQLLAALDYLTT<br>DSTVRDRIDPNRMAVMGHSMG<br>GGGALSAAANNPSLKAAIPLQP<br>WHTRKDWSSVRVPTLIVGAQL<br>DTIAPVSSHAEAFYNSLPSSLPK<br>AYMELRGADHLVSNTPNTTTAK<br>YSIAWLKRFVDDDTRYEQFLCP<br>APDDPAISEYRSTCPFLEHHHH<br>HH
**R2M2-Q6E**	ATGGCGAACCCGTATCAACGTGGTCCGGATCCGACCGAGAGCAGCATTGAAGCGGTGCGTGGTCCGTT<br>TGCGGTGGCGCAAACCACCGTTAGCCGTCTGGATGCGAGCGGCTTTGGTGGCGGTACCATCTACTATC<br>CGACCGACACCAGCCAGGGTACCTTTGGTGCGGTTGCGATTAGCCCGGGCTTTACCGCGGGTCAGAG<br>CAGCATTGCGTGGCTGGGTCCGCGTATTGCGAGCCAAGGTTTCGTGGTTATCACCATTGACACCATCA<br>GCCGTCTGGATCAGCCGGACAGCCGTGGCCGTCAACTGCTGGCGGCGCTGGATTATCTGACCACCGA<br>CAGCACCGTGCGTGATCGTATTGACCCGAACCGTATGGCGGTTATGGGTCACAGCATGGGCGGTGGC<br>GGTGCGCTGAGCGCGGCGGCGAACAACCCGAGCCTGAAAGCGGCGATTCCGCTGCAGCCGTGGCAC<br>ACCCGTAAAGATTGGAGCAGCGTGCGTGTTCCGACCCTGATCGTGGGCGCGCAACTGGACACCATTGC<br>GCCGGTTAGCAGCCATGCGGAGGCGTTCTACAACAGCCTGCCGAGCAGCCTGGACAAGGCGTATATG<br>GAACTGCGTGGTGCGGATCACCTGGTTAGCAACACCCCGAACACCACCACCGCGAAGTACAGCATCGC<br>GTGGCTGAAACGTTTCGTTGACGATGACACCCGTTATGAGCAATTTCTGTGCCCGGCGCCGGATGACC<br>CGGCGATTAGCGAATACCGTAGCACCTGCCCGTTTCTCGAGCACCACCACCACCACCACTGA	MANPYERGPDPTESSIEAVRGP<br>FAVAQTTVSRLDASGFGGGTIY<br>YPTDTSQGTFGAVAISPGFTAG<br>QSSIAWLGPRIASQGFVVITIDTI<br>SRLDQPDSRGRQLLAALDYLTT<br>DSTVRDRIDPNRMAVMGHSMG<br>GGGALSAAANNPSLKAAIPLQP<br>WHTRKDWSSVRVPTLIVGAQL<br>DTIAPVSSHAEAFYNSLPSSLPK<br>AYMELRGADHLVSNTPNTTTAK<br>YSIAWLKRFVDDDTRYEQFLCP<br>APDDPAISEYRSTCPFLEHHHH<br>HH
**R2M2-Q6E+P198D**	ATGGCGAACCCGTATGAACGTGGTCCGGATCCGACCGAGAGCAGCATTGAAGCGGTGCGTGGTCCGT<br>TTGCGGTGGCGCAAACCACCGTTAGCCGTCTGGATGCGAGCGGCTTTGGTGGCGGTACCATCTACTAT<br>CCGACCGACACCAGCCAGGGTACCTTTGGTGCGGTTGCGATTAGCCCGGGCTTTACCGCGGGTCAGA<br>GCAGCATTGCGTGGCTGGGTCCGCGTATTGCGAGCCAAGGTTTCGTGGTTATCACCATTGACACCATC<br>AGCCGTCTGGATCAGCCGGACAGCCGTGGCCGTCAACTGCTGGCGGCGCTGGATTATCTGACCACCG<br>ACAGCACCGTGCGTGATCGTATTGACCCGAACCGTATGGCGGTTATGGGTCACAGCATGGGCGGTGGC<br>GGTGCGCTGAGCGCGGCGGCGAACAACCCGAGCCTGAAAGCGGCGATTCCGCTGCAGCCGTGGCAC	MANPYERGPDPTESSIEAVRGP<br>FAVAQTTVSRLDASGFGGGTIY<br>YPTDTSQGTFGAVAISPGFTAG<br>QSSIAWLGPRIASQGFVVITIDTI<br>SRLDQPDSRGRQLLAALDYLTT<br>DSTVRDRIDPNRMAVMGHSMG<br>GGGALSAAANNPSLKAAIPLQP

	ACCCGTAAAGATTGGAGCAGCGTGCGTGTTCCGACCCTGATCGTGGGCGCGCAACTGGACACCATTGC<br>GCCGGTTAGCAGCCATGCGGAGGCGTTCTACAACAGCCTGCCGAGCAGCCTGGACAAGGCGTATATG<br>GAACTGCGTGGTGCGGATCACCTGGTTAGCAACACCCCGAACACCACCACCGCGAAGTACAGCATCGC<br>GTGGCTGAAACGTTTCGTTGACGATGACACCCGTTATGAGCAATTTCTGTGCCCGGCGCCGGATGACC<br>CGGCGATTAGCGAATACCGTAGCACCTGCCCGTTTCTCGAGCACCACCACCACCACCACTGA	WHTRKDWSSVRVPTLIVGAQL<br>DTIAPVSSHAEAFYNSLPSSLDK<br>AYMELRGADHLVSNTPNTTTAK<br>YSIAWLKRFVDDDTRYEQFLCP<br>APDDPAISEYRSTCPFLEHHHH<br>HH
**R2M2-Q6E+P198D+S196D**	ATGGCTAACCCGTATGAGAGAGGACCCGACCCGACGGAGAGTTCAATCGAAGCCGTTAGAGGACCCTT<br>CGCCGTAGCGCAGACCACCGTGTCCCGCCTTGATGCTTCCGGTTTCGGCGGCGGCACGATTTACTACC<br>CTACCGACACGAGTCAGGGCACTTTTGGCGCGGTGGCGATTTCACCAGGGTTCACTGCTGGGCAGTCG<br>TCGATAGCATGGTTGGGGCCAAGAATCGCTTCACAGGGATTTGTGGTGATAACAATAGACACTATTTCA<br>CGGCTTGATCAACCTGATTCCAGAGGTCGTCAGCTGCTGGCCGCACTTGACTATCTTACCACAGATTCG<br>ACGGTTCGGGATCGGATCGATCCAAATCGGATGGCGGTCATGGGGCATAGTATGGGTGGTGGCGGCG<br>CACTGAGCGCTGCTGCTAATAATCCGTCGTTAAAAGCCGCGATACCGTTGCAGCCGTGGCATACAAGAA<br>AGGATTGGAGCTCAGTACGTGTGCCGACTCTTATTGTGGGTGCTCAACTGGATACCATCGCCCCGGTAA<br>GTAGCCATGCGGAGGCTTTCTACAATTCCTTACCTTCAGATTTGGACAAAGCTTACATGGAACTTCGGG<br>GCGCAGACCACCTGGTTTCGAACACTCCAAATACAACGACCGCCAAATATTCTATAGCCTGGCTGAAGC<br>GGTTCGTTGACGACGATACTAGATACGAGCAGTTCCTTTGCCCGGCTCCCGATGACCCCGCGATTTCG<br>GAGTACAGATCCACCTGTCCATTTCTCGAGCACCACCACCACCACCACTGA	MANPYERGPDPTESSIEAVRGP<br>FAVAQTTVSRLDASGFGGGTIY<br>YPTDTSQGTFGAVAISPGFTAG<br>QSSIAWLGPRIASQGFVVITIDTI<br>SRLDQPDSRGRQLLAALDYLTT<br>DSTVRDRIDPNRMAVMGHSMG<br>GGGALSAAANNPSLKAAIPLQP<br>WHTRKDWSSVRVPTLIVGAQL<br>DTIAPVSSHAEAFYNSLPSDLDK<br>AYMELRGADHLVSNTPNTTTAK<br>YSIAWLKRFVDDDTRYEQFLCP<br>APDDPAISEYRSTCPFLEHHHH<br>HH
**R2M2-Q6E+P198D+S196D+K148E**	ATGGCTAACCCGTATGAGAGAGGACCCGACCCGACGGAGAGTTCAATCGAAGCCGTTAGAGGACCCTT<br>CGCCGTAGCGCAGACCACCGTGTCCCGCCTTGATGCTTCCGGTTTCGGCGGCGGCACGATTTACTACC<br>CTACCGACACGAGTCAGGGCACTTTTGGCGCGGTGGCGATTTCACCAGGGTTCACTGCTGGGCAGTCG<br>TCGATAGCATGGTTGGGGCCAAGAATCGCTTCACAGGGATTTGTGGTGATAACAATAGACACTATTTCA<br>CGGCTTGATCAACCTGATTCCAGAGGTCGTCAGCTGCTGGCCGCACTTGACTATCTTACCACAGATTCG<br>ACGGTTCGGGATCGGATCGATCCAAATCGGATGGCGGTCATGGGGCATAGTATGGGTGGTGGCGGCG<br>CACTGAGCGCTGCTGCTAATAATCCGTCGTTAGAGGCCGCGATACCGTTGCAGCCGTGGCATACAAGA<br>AAGGATTGGAGCTCAGTACGTGTGCCGACTCTTATTGTGGGTGCTCAACTGGATACCATCGCCCCGGTA<br>AGTAGCCATGCGGAGGCTTTCTACAATTCCTTACCTTCAGATTTGGACAAAGCTTACATGGAACTTCGG<br>GGCGCAGACCACCTGGTTTCGAACACTCCAAATACAACGACCGCCAAATATTCTATAGCCTGGCTGAAG<br>CGGTTCGTTGACGACGATACTAGATACGAGCAGTTCCTTTGCCCGGCTCCCGATGACCCCGCGATTTC<br>GGAGTACAGATCCACCTGTCCATTTCTCGAGCACCACCACCACCACCACTGA	MANPYERGPDPTESSIEAVRGP<br>FAVAQTTVSRLDASGFGGGTIY<br>YPTDTSQGTFGAVAISPGFTAG<br>QSSIAWLGPRIASQGFVVITIDTI<br>SRLDQPDSRGRQLLAALDYLTT<br>DSTVRDRIDPNRMAVMGHSMG<br>GGGALSAAANNPSLEAAIPLQP<br>WHTRKDWSSVRVPTLIVGAQL<br>DTIAPVSSHAEAFYNSLPSDLDK<br>AYMELRGADHLVSNTPNTTTAK<br>YSIAWLKRFVDDDTRYEQFLCP<br>APDDPAISEYRSTCPFLEHHHH<br>HH
**R3M1 (=R2M2-S68E)**	ATGGCGAACCCGTATCAACGTGGTCCGGATCCGACCGAGAGCAGCATTGAAGCGGTGCGTGGTCCGTT<br>TGCGGTGGCGCAAACCACCGTTAGCCGTCTGGATGCGAGCGGCTTTGGTGGCGGTACCATCTACTATC<br>CGACCGACACCAGCCAGGGTACCTTTGGTGCGGTTGCGATTAGCCCGGGCTTTACCGCGGGTCAGGA<br>GAGCATTGCGTGGCTGGGTCCGCGTATTGCGAGCCAAGGTTTCGTGGTTATCACCATTGACACCATCA<br>GCCGTCTGGATCAGCCGGACAGCCGTGGCCGTCAACTGCTGGCGGCGCTGGATTATCTGACCACCGA<br>CAGCACCGTGCGTGATCGTATTGACCCGAACCGTATGGCGGTTATGGGTCACAGCATGGGCGGTGGC<br>GGTGCGCTGAGCGCGGCGGCGAACAACCCGAGCCTGAAAGCGGCGATTCCGCTGCAGCCGTGGCAC<br>ACCCGTAAAGATTGGAGCAGCGTGCGTGTTCCGACCCTGATCGTGGGCGCGCAACTGGACACCATTGC<br>GCCGGTTAGCAGCCATGCGGAGGCGTTCTACAACAGCCTGCCGAGCAGCCTGCCGAAGGCGTATATG<br>GAACTGCGTGGTGCGGATCACCTGGTTAGCAACACCCCGAACACCACCACCGCGAAGTACAGCATCGC<br>GTGGCTGAAACGTTTCGTTGACGATGACACCCGTTATGAGCAATTTCTGTGCCCGGCGCCGGATGACC<br>CGGCGATTAGCGAATACCGTAGCACCTGCCCGTTTCTCGAGCACCACCACCACCACCACTGA	MANPYQRGPDPTESSIEAVRGP<br>FAVAQTTVSRLDASGFGGGTIY<br>YPTDTSQGTFGAVAISPGFTAG<br>QESIAWLGPRIASQGFVVITIDTI<br>SRLDQPDSRGRQLLAALDYLTT<br>DSTVRDRIDPNRMAVMGHSMG<br>GGGALSAAANNPSLKAAIPLQP<br>WHTRKDWSSVRVPTLIVGAQL<br>DTIAPVSSHAEAFYNSLPSSLPK<br>AYMELRGADHLVSNTPNTTTAK<br>YSIAWLKRFVDDDTRYEQFLCP<br>APDDPAISEYRSTCPFLEHHHH<br>HH
**R3M2 (=R2M2-P155G)**	ATGGCGAACCCGTATCAACGTGGTCCGGATCCGACCGAGAGCAGCATTGAAGCGGTGCGTGGTCCGTT<br>TGCGGTGGCGCAAACCACCGTTAGCCGTCTGGATGCGAGCGGCTTTGGTGGCGGTACCATCTACTATC<br>CGACCGACACCAGCCAGGGTACCTTTGGTGCGGTTGCGATTAGCCCGGGCTTTACCGCGGGTCAGAG<br>CAGCATTGCGTGGCTGGGTCCGCGTATTGCGAGCCAAGGTTTCGTGGTTATCACCATTGACACCATCA<br>GCCGTCTGGATCAGCCGGACAGCCGTGGCCGTCAACTGCTGGCGGCGCTGGATTATCTGACCACCGA<br>CAGCACCGTGCGTGATCGTATTGACCCGAACCGTATGGCGGTTATGGGTCACAGCATGGGCGGTGGC<br>GGTGCGCTGAGCGCGGCGGCGAACAACCCGAGCCTGAAAGCGGCGATTCCGCTGCAGGGGTGGCAC<br>ACCCGTAAAGATTGGAGCAGCGTGCGTGTTCCGACCCTGATCGTGGGCGCGCAACTGGACACCATTGC<br>GCCGGTTAGCAGCCATGCGGAGGCGTTCTACAACAGCCTGCCGAGCAGCCTGCCGAAGGCGTATATG<br>GAACTGCGTGGTGCGGATCACCTGGTTAGCAACACCCCGAACACCACCACCGCGAAGTACAGCATCGC<br>GTGGCTGAAACGTTTCGTTGACGATGACACCCGTTATGAGCAATTTCTGTGCCCGGCGCCGGATGACC<br>CGGCGATTAGCGAATACCGTAGCACCTGCCCGTTTCTCGAGCACCACCACCACCACCACTGA	MANPYQRGPDPTESSIEAVRGP<br>FAVAQTTVSRLDASGFGGGTIY<br>YPTDTSQGTFGAVAISPGFTAG<br>QSSIAWLGPRIASQGFVVITIDTI<br>SRLDQPDSRGRQLLAALDYLTT<br>DSTVRDRIDPNRMAVMGHSMG<br>GGGALSAAANNPSLKAAIPLQG<br>WHTRKDWSSVRVPTLIVGAQL<br>DTIAPVSSHAEAFYNSLPSSLPK<br>AYMELRGADHLVSNTPNTTTAK<br>YSIAWLKRFVDDDTRYEQFLCP<br>APDDPAISEYRSTCPFLEHHHH<br>HH
**R3M3 (=R2M2-I171V)**	ATGGCGAACCCGTATCAACGTGGTCCGGATCCGACCGAGAGCAGCATTGAAGCGGTGCGTGGTCCGTT<br>TGCGGTGGCGCAAACCACCGTTAGCCGTCTGGATGCGAGCGGCTTTGGTGGCGGTACCATCTACTATC<br>CGACCGACACCAGCCAGGGTACCTTTGGTGCGGTTGCGATTAGCCCGGGCTTTACCGCGGGTCAGAG<br>CAGCATTGCGTGGCTGGGTCCGCGTATTGCGAGCCAAGGTTTCGTGGTTATCACCATTGACACCATCA<br>GCCGTCTGGATCAGCCGGACAGCCGTGGCCGTCAACTGCTGGCGGCGCTGGATTATCTGACCACCGA<br>CAGCACCGTGCGTGATCGTATTGACCCGAACCGTATGGCGGTTATGGGTCACAGCATGGGCGGTGGC<br>GGTGCGCTGAGCGCGGCGGCGAACAACCCGAGCCTGAAAGCGGCGATTCCGCTGCAGCCGTGGCAC<br>ACCCGTAAAGATTGGAGCAGCGTGCGTGTTCCGACCCTGGTCGTGGGCGCGCAACTGGACACCATTGC<br>GCCGGTTAGCAGCCATGCGGAGGCGTTCTACAACAGCCTGCCGAGCAGCCTGCCGAAGGCGTATATG<br>GAACTGCGTGGTGCGGATCACCTGGTTAGCAACACCCCGAACACCACCACCGCGAAGTACAGCATCGC<br>GTGGCTGAAACGTTTCGTTGACGATGACACCCGTTATGAGCAATTTCTGTGCCCGGCGCCGGATGACC<br>CGGCGATTAGCGAATACCGTAGCACCTGCCCGTTTCTCGAGCACCACCACCACCACCACTGA	MANPYQRGPDPTESSIEAVRGP<br>FAVAQTTVSRLDASGFGGGTIY<br>YPTDTSQGTFGAVAISPGFTAG<br>QSSIAWLGPRIASQGFVVITIDTI<br>SRLDQPDSRGRQLLAALDYLTT<br>DSTVRDRIDPNRMAVMGHSMG<br>GGGALSAAANNPSLKAAIPLQP<br>WHTRKDWSSVRVPTLVVGAQL<br>DTIAPVSSHAEAFYNSLPSSLPK<br>AYMELRGADHLVSNTPNTTTAK<br>YSIAWLKRFVDDDTRYEQFLCP<br>APDDPAISEYRSTCPFLEHHHH<br>HH
**R3M4 (=R2M2-A186S)**	ATGGCGAACCCGTATCAACGTGGTCCGGATCCGACCGAGAGCAGCATTGAAGCGGTGCGTGGTCCGTT<br>TGCGGTGGCGCAAACCACCGTTAGCCGTCTGGATGCGAGCGGCTTTGGTGGCGGTACCATCTACTATC<br>CGACCGACACCAGCCAGGGTACCTTTGGTGCGGTTGCGATTAGCCCGGGCTTTACCGCGGGTCAGAG<br>CAGCATTGCGTGGCTGGGTCCGCGTATTGCGAGCCAAGGTTTCGTGGTTATCACCATTGACACCATCA<br>GCCGTCTGGATCAGCCGGACAGCCGTGGCCGTCAACTGCTGGCGGCGCTGGATTATCTGACCACCGA<br>CAGCACCGTGCGTGATCGTATTGACCCGAACCGTATGGCGGTTATGGGTCACAGCATGGGCGGTGGC<br>GGTGCGCTGAGCGCGGCGGCGAACAACCCGAGCCTGAAAGCGGCGATTCCGCTGCAGCCGTGGCAC<br>ACCCGTAAAGATTGGAGCAGCGTGCGTGTTCCGACCCTGATCGTGGGCGCGCAACTGGACACCATTGC	MANPYQRGPDPTESSIEAVRGP<br>FAVAQTTVSRLDASGFGGGTIY<br>YPTDTSQGTFGAVAISPGFTAG<br>QSSIAWLGPRIASQGFVVITIDTI<br>SRLDQPDSRGRQLLAALDYLTT<br>DSTVRDRIDPNRMAVMGHSMG<br>GGGALSAAANNPSLKAAIPLQP<br>WHTRKDWSSVRVPTLIVGAQL

	GCCGGTTAGCAGCCATTCGGAGGCGTTCTACAACAGCCTGCCGAGCAGCCTGCCGAAGGCGTATATG<br>GAACTGCGTGGTGCGGATCACCTGGTTAGCAACACCCCGAACACCACCACCGCGAAGTACAGCATCGC<br>GTGGCTGAAACGTTTCGTTGACGATGACACCCGTTATGAGCAATTTCTGTGCCCGGCGCCGGATGACC<br>CGGCGATTAGCGAATACCGTAGCACCTGCCCGTTTCTCGAGCACCACCACCACCACCACTGA	DTIAPVSSHSEAFYNSLPSSLPK<br>AYMELRGADHLVSNTPNTTTAK<br>YSIAWLKRFVDDDTRYEQFLCP<br>APDDPAISEYRSTCPFLEHHHH<br>HH
**R3M5 (=R2M2-D208S)**	ATGGCGAACCCGTATCAACGTGGTCCGGATCCGACCGAGAGCAGCATTGAAGCGGTGCGTGGTCCGTT<br>TGCGGTGGCGCAAACCACCGTTAGCCGTCTGGATGCGAGCGGCTTTGGTGGCGGTACCATCTACTATC<br>CGACCGACACCAGCCAGGGTACCTTTGGTGCGGTTGCGATTAGCCCGGGCTTTACCGCGGGTCAGAG<br>CAGCATTGCGTGGCTGGGTCCGCGTATTGCGAGCCAAGGTTTCGTGGTTATCACCATTGACACCATCA<br>GCCGTCTGGATCAGCCGGACAGCCGTGGCCGTCAACTGCTGGCGGCGCTGGATTATCTGACCACCGA<br>CAGCACCGTGCGTGATCGTATTGACCCGAACCGTATGGCGGTTATGGGTCACAGCATGGGCGGTGGC<br>GGTGCGCTGAGCGCGGCGGCGAACAACCCGAGCCTGAAAGCGGCGATTCCGCTGCAGCCGTGGCAC<br>ACCCGTAAAGATTGGAGCAGCGTGCGTGTTCCGACCCTGATCGTGGGCGCGCAACTGGACACCATTGC<br>GCCGGTTAGCAGCCATGCGGAGGCGTTCTACAACAGCCTGCCGAGCAGCCTGCCGAAGGCGTATATG<br>GAACTGCGTGGTGCGAGTCACCTGGTTAGCAACACCCCGAACACCACCACCGCGAAGTACAGCATCGC<br>GTGGCTGAAACGTTTCGTTGACGATGACACCCGTTATGAGCAATTTCTGTGCCCGGCGCCGGATGACC<br>CGGCGATTAGCGAATACCGTAGCACCTGCCCGTTTCTCGAGCACCACCACCACCACCACTGA	MANPYQRGPDPTESSIEAVRGP<br>FAVAQTTVSRLDASGFGGGTIY<br>YPTDTSQGTFGAVAISPGFTAG<br>QSSIAWLGPRIASQGFVVITIDTI<br>SRLDQPDSRGRQLLAALDYLTT<br>DSTVRDRIDPNRMAVMGHSMG<br>GGGALSAAANNPSLKAAIPLQP<br>WHTRKDWSSVRVPTLIVGAQL<br>DTIAPVSSHAEAFYNSLPSSLPK<br>AYMELRGASHLVSNTPNTTTAK<br>YSIAWLKRFVDDDTRYEQFLCP<br>APDDPAISEYRSTCPFLEHHHH<br>HH
**R3M6 (=R2M2-P155G+A186S)**	ATGGCGAACCCGTATCAACGTGGTCCGGATCCGACCGAGAGCAGCATTGAAGCGGTGCGTGGTCCGTT<br>TGCGGTGGCGCAAACCACCGTTAGCCGTCTGGATGCGAGCGGCTTTGGTGGCGGTACCATCTACTATC<br>CGACCGACACCAGCCAGGGTACCTTTGGTGCGGTTGCGATTAGCCCGGGCTTTACCGCGGGTCAGAG<br>CAGCATTGCGTGGCTGGGTCCGCGTATTGCGAGCCAAGGTTTCGTGGTTATCACCATTGACACCATCA<br>GCCGTCTGGATCAGCCGGACAGCCGTGGCCGTCAACTGCTGGCGGCGCTGGATTATCTGACCACCGA<br>CAGCACCGTGCGTGATCGTATTGACCCGAACCGTATGGCGGTTATGGGTCACAGCATGGGCGGTGGC<br>GGTGCGCTGAGCGCGGCGGCGAACAACCCGAGCCTGAAAGCGGCGATTCCGCTGCAGGGGTGGCAC<br>ACCCGTAAAGATTGGAGCAGCGTGCGTGTTCCGACCCTGATCGTGGGCGCGCAACTGGACACCATTGC<br>GCCGGTTAGCAGCCATTCGGAGGCGTTCTACAACAGCCTGCCGAGCAGCCTGCCGAAGGCGTATATG<br>GAACTGCGTGGTGCGGATCACCTGGTTAGCAACACCCCGAACACCACCACCGCGAAGTACAGCATCGC<br>GTGGCTGAAACGTTTCGTTGACGATGACACCCGTTATGAGCAATTTCTGTGCCCGGCGCCGGATGACC<br>CGGCGATTAGCGAATACCGTAGCACCTGCCCGTTTCTCGAGCACCACCACCACCACCACTGA	MANPYQRGPDPTESSIEAVRGP<br>FAVAQTTVSRLDASGFGGGTIY<br>YPTDTSQGTFGAVAISPGFTAG<br>QSSIAWLGPRIASQGFVVITIDTI<br>SRLDQPDSRGRQLLAALDYLTT<br>DSTVRDRIDPNRMAVMGHSMG<br>GGGALSAAANNPSLKAAIPLQG<br>WHTRKDWSSVRVPTLIVGAQL<br>DTIAPVSSHSEAFYNSLPSSLPK<br>AYMELRGADHLVSNTPNTTTAK<br>YSIAWLKRFVDDDTRYEQFLCP<br>APDDPAISEYRSTCPFLEHHHH<br>HH
**R3M7 (=R2M2-P155G+D208S)**	ATGGCGAACCCGTATCAACGTGGTCCGGATCCGACCGAGAGCAGCATTGAAGCGGTGCGTGGTCCGTT<br>TGCGGTGGCGCAAACCACCGTTAGCCGTCTGGATGCGAGCGGCTTTGGTGGCGGTACCATCTACTATC<br>CGACCGACACCAGCCAGGGTACCTTTGGTGCGGTTGCGATTAGCCCGGGCTTTACCGCGGGTCAGAG<br>CAGCATTGCGTGGCTGGGTCCGCGTATTGCGAGCCAAGGTTTCGTGGTTATCACCATTGACACCATCA<br>GCCGTCTGGATCAGCCGGACAGCCGTGGCCGTCAACTGCTGGCGGCGCTGGATTATCTGACCACCGA<br>CAGCACCGTGCGTGATCGTATTGACCCGAACCGTATGGCGGTTATGGGTCACAGCATGGGCGGTGGC<br>GGTGCGCTGAGCGCGGCGGCGAACAACCCGAGCCTGAAAGCGGCGATTCCGCTGCAGGGGTGGCAC<br>ACCCGTAAAGATTGGAGCAGCGTGCGTGTTCCGACCCTGATCGTGGGCGCGCAACTGGACACCATTGC<br>GCCGGTTAGCAGCCATGCGGAGGCGTTCTACAACAGCCTGCCGAGCAGCCTGCCGAAGGCGTATATG<br>GAACTGCGTGGTGCGAGTCACCTGGTTAGCAACACCCCGAACACCACCACCGCGAAGTACAGCATCGC<br>GTGGCTGAAACGTTTCGTTGACGATGACACCCGTTATGAGCAATTTCTGTGCCCGGCGCCGGATGACC<br>CGGCGATTAGCGAATACCGTAGCACCTGCCCGTTTCTCGAGCACCACCACCACCACCACTGA	MANPYQRGPDPTESSIEAVRGP<br>FAVAQTTVSRLDASGFGGGTIY<br>YPTDTSQGTFGAVAISPGFTAG<br>QSSIAWLGPRIASQGFVVITIDTI<br>SRLDQPDSRGRQLLAALDYLTT<br>DSTVRDRIDPNRMAVMGHSMG<br>GGGALSAAANNPSLKAAIPLQG<br>WHTRKDWSSVRVPTLIVGAQL<br>DTIAPVSSHAEAFYNSLPSSLPK<br>AYMELRGASHLVSNTPNTTTAK<br>YSIAWLKRFVDDDTRYEQFLCP<br>APDDPAISEYRSTCPFLEHHHH<br>HH
**R3M8 (R2M2-A186S+D208S)**	ATGGCGAACCCGTATCAACGTGGTCCGGATCCGACCGAGAGCAGCATTGAAGCGGTGCGTGGTCCGTT<br>TGCGGTGGCGCAAACCACCGTTAGCCGTCTGGATGCGAGCGGCTTTGGTGGCGGTACCATCTACTATC<br>CGACCGACACCAGCCAGGGTACCTTTGGTGCGGTTGCGATTAGCCCGGGCTTTACCGCGGGTCAGAG<br>CAGCATTGCGTGGCTGGGTCCGCGTATTGCGAGCCAAGGTTTCGTGGTTATCACCATTGACACCATCA<br>GCCGTCTGGATCAGCCGGACAGCCGTGGCCGTCAACTGCTGGCGGCGCTGGATTATCTGACCACCGA<br>CAGCACCGTGCGTGATCGTATTGACCCGAACCGTATGGCGGTTATGGGTCACAGCATGGGCGGTGGC<br>GGTGCGCTGAGCGCGGCGGCGAACAACCCGAGCCTGAAAGCGGCGATTCCGCTGCAGCCGTGGCAC<br>ACCCGTAAAGATTGGAGCAGCGTGCGTGTTCCGACCCTGATCGTGGGCGCGCAACTGGACACCATTGC<br>GCCGGTTAGCAGCCATTCGGAGGCGTTCTACAACAGCCTGCCGAGCAGCCTGCCGAAGGCGTATATG<br>GAACTGCGTGGTGCGAGTCACCTGGTTAGCAACACCCCGAACACCACCACCGCGAAGTACAGCATCGC<br>GTGGCTGAAACGTTTCGTTGACGATGACACCCGTTATGAGCAATTTCTGTGCCCGGCGCCGGATGACC<br>CGGCGATTAGCGAATACCGTAGCACCTGCCCGTTTCTCGAGCACCACCACCACCACCACTGA	MANPYQRGPDPTESSIEAVRGP<br>FAVAQTTVSRLDASGFGGGTIY<br>YPTDTSQGTFGAVAISPGFTAG<br>QSSIAWLGPRIASQGFVVITIDTI<br>SRLDQPDSRGRQLLAALDYLTT<br>DSTVRDRIDPNRMAVMGHSMG<br>GGGALSAAANNPSLKAAIPLQP<br>WHTRKDWSSVRVPTLIVGAQL<br>DTIAPVSSHSEAFYNSLPSSLPK<br>AYMELRGASHLVSNTPNTTTAK<br>YSIAWLKRFVDDDTRYEQFLCP<br>APDDPAISEYRSTCPFLEHHHH<br>HH
**R3M9 (=R2M2-P155G+A186S+D208S)**	ATGGCGAACCCGTATCAACGTGGTCCGGATCCGACCGAGAGCAGCATTGAAGCGGTGCGTGGTCCGTT<br>TGCGGTGGCGCAAACCACCGTTAGCCGTCTGGATGCGAGCGGCTTTGGTGGCGGTACCATCTACTATC<br>CGACCGACACCAGCCAGGGTACCTTTGGTGCGGTTGCGATTAGCCCGGGCTTTACCGCGGGTCAGAG<br>CAGCATTGCGTGGCTGGGTCCGCGTATTGCGAGCCAAGGTTTCGTGGTTATCACCATTGACACCATCA<br>GCCGTCTGGATCAGCCGGACAGCCGTGGCCGTCAACTGCTGGCGGCGCTGGATTATCTGACCACCGA<br>CAGCACCGTGCGTGATCGTATTGACCCGAACCGTATGGCGGTTATGGGTCACAGCATGGGCGGTGGC<br>GGTGCGCTGAGCGCGGCGGCGAACAACCCGAGCCTGAAAGCGGCGATTCCGCTGCAGGGGTGGCAC<br>ACCCGTAAAGATTGGAGCAGCGTGCGTGTTCCGACCCTGATCGTGGGCGCGCAACTGGACACCATTGC<br>GCCGGTTAGCAGCCATTCGGAGGCGTTCTACAACAGCCTGCCGAGCAGCCTGCCGAAGGCGTATATG<br>GAACTGCGTGGTGCGAGTCACCTGGTTAGCAACACCCCGAACACCACCACCGCGAAGTACAGCATCGC<br>GTGGCTGAAACGTTTCGTTGACGATGACACCCGTTATGAGCAATTTCTGTGCCCGGCGCCGGATGACC<br>CGGCGATTAGCGAATACCGTAGCACCTGCCCGTTTCTCGAGCACCACCACCACCACCACTGA	MANPYQRGPDPTESSIEAVRGP<br>FAVAQTTVSRLDASGFGGGTIY<br>YPTDTSQGTFGAVAISPGFTAG<br>QSSIAWLGPRIASQGFVVITIDTI<br>SRLDQPDSRGRQLLAALDYLTT<br>DSTVRDRIDPNRMAVMGHSMG<br>GGGALSAAANNPSLKAAIPLQG<br>WHTRKDWSSVRVPTLIVGAQL<br>DTIAPVSSHSEAFYNSLPSSLPK<br>AYMELRGASHLVSNTPNTTTAK<br>YSIAWLKRFVDDDTRYEQFLCP<br>APDDPAISEYRSTCPFLEHHHH<br>HH
**R4M1 (=R2M2-A186S+L104Q)**	ATGGCGAACCCGTATCAACGTGGTCCGGATCCGACCGAGAGCAGCATTGAAGCGGTGCGTGGTCCGTT<br>TGCGGTGGCGCAAACCACCGTTAGCCGTCTGGATGCGAGCGGCTTTGGTGGCGGTACCATCTACTATC<br>CGACCGACACCAGCCAGGGTACCTTTGGTGCGGTTGCGATTAGCCCGGGCTTTACCGCGGGTCAGAG<br>CAGCATTGCGTGGCTGGGTCCGCGTATTGCGAGCCAAGGTTTCGTGGTTATCACCATTGACACCATCA<br>GCCGTCTGGATCAGCCGGACAGCCGTGGCCGTCAACTGCAGGCGGCGCTGGATTATCTGACCACCGA<br>CAGCACCGTGCGTGATCGTATTGACCCGAACCGTATGGCGGTTATGGGTCACAGCATGGGCGGTGGC<br>GGTGCGCTGAGCGCGGCGGCGAACAACCCGAGCCTGAAAGCGGCGATTCCGCTGCAGCCGTGGCAC<br>ACCCGTAAAGATTGGAGCAGCGTGCGTGTTCCGACCCTGATCGTGGGCGCGCAACTGGACACCATTGC<br>GCCGGTTAGCAGCCATTCGGAGGCGTTCTACAACAGCCTGCCGAGCAGCCTGCCGAAGGCGTATATG	MANPYQRGPDPTESSIEAVRGP<br>FAVAQTTVSRLDASGFGGGTIY<br>YPTDTSQGTFGAVAISPGFTAG<br>QSSIAWLGPRIASQGFVVITIDTI<br>SRLDQPDSRGRQLQAALDYLTT<br>DSTVRDRIDPNRMAVMGHSMG<br>GGGALSAAANNPSLKAAIPLQP<br>WHTRKDWSSVRVPTLIVGAQL<br>DTIAPVSSHSEAFYNSLPSSLPK

	GAACTGCGTGGTGCGGATCACCTGGTTAGCAACACCCCGAACACCACCACCGCGAAGTACAGCATCGC<br>GTGGCTGAAACGTTTCGTTGACGATGACACCCGTTATGAGCAATTTCTGTGCCCGGCGCCGGATGACC<br>CGGCGATTAGCGAATACCGTAGCACCTGCCCGTTTCTCGAGCACCACCACCACCACCACTGA	AYMELRGADHLVSNTPNTTTAK<br>YSIAWLKRFVDDDTRYEQFLCP<br>APDDPAISEYRSTCPFLEHHHH<br>HH
**R4M2 (=R2M2-A186S+L104Q+Q175E)**	ATGGCGAACCCGTATCAACGTGGTCCGGATCCGACCGAGAGCAGCATTGAAGCGGTGCGTGGTCCGTT<br>TGCGGTGGCGCAAACCACCGTTAGCCGTCTGGATGCGAGCGGCTTTGGTGGCGGTACCATCTACTATC<br>CGACCGACACCAGCCAGGGTACCTTTGGTGCGGTTGCGATTAGCCCGGGCTTTACCGCGGGTCAGAG<br>CAGCATTGCGTGGCTGGGTCCGCGTATTGCGAGCCAAGGTTTCGTGGTTATCACCATTGACACCATCA<br>GCCGTCTGGATCAGCCGGACAGCCGTGGCCGTCAACTGCAGGCGGCGCTGGATTATCTGACCACCGA<br>CAGCACCGTGCGTGATCGTATTGACCCGAACCGTATGGCGGTTATGGGTCACAGCATGGGCGGTGGC<br>GGTGCGCTGAGCGCGGCGGCGAACAACCCGAGCCTGAAAGCGGCGATTCCGCTGCAGCCGTGGCAC<br>ACCCGTAAAGATTGGAGCAGCGTGCGTGTTCCGACCCTGATCGTGGGCGCGGAACTGGACACCATTGC<br>GCCGGTTAGCAGCCATTCGGAGGCGTTCTACAACAGCCTGCCGAGCAGCCTGCCGAAGGCGTATATG<br>GAACTGCGTGGTGCGGATCACCTGGTTAGCAACACCCCGAACACCACCACCGCGAAGTACAGCATCGC<br>GTGGCTGAAACGTTTCGTTGACGATGACACCCGTTATGAGCAATTTCTGTGCCCGGCGCCGGATGACC<br>CGGCGATTAGCGAATACCGTAGCACCTGCCCGTTTCTCGAGCACCACCACCACCACCACTGA	MANPYQRGPDPTESSIEAVRGP<br>FAVAQTTVSRLDASGFGGGTIY<br>YPTDTSQGTFGAVAISPGFTAG<br>QSSIAWLGPRIASQGFVVITIDTI<br>SRLDQPDSRGRQLQAALDYLTT<br>DSTVRDRIDPNRMAVMGHSMG<br>GGGALSAAANNPSLKAAIPLQP<br>WHTRKDWSSVRVPTLIVGAELD<br>TIAPVSSHSEAFYNSLPSSLPKA<br>YMELRGADHLVSNTPNTTTAKY<br>SIAWLKRFVDDDTRYEQFLCPA<br>PDDPAISEYRSTCPFLEHHHHH<br>H
**R4M3 (=R2M2-A186S+L104Q+Q175E+L210T)**	ATGGCGAACCCGTATCAACGTGGTCCGGATCCGACCGAGAGCAGCATTGAAGCGGTGCGTGGTCCGTT<br>TGCGGTGGCGCAAACCACCGTTAGCCGTCTGGATGCGAGCGGCTTTGGTGGCGGTACCATCTACTATC<br>CGACCGACACCAGCCAGGGTACCTTTGGTGCGGTTGCGATTAGCCCGGGCTTTACCGCGGGTCAGAG<br>CAGCATTGCGTGGCTGGGTCCGCGTATTGCGAGCCAAGGTTTCGTGGTTATCACCATTGACACCATCA<br>GCCGTCTGGATCAGCCGGACAGCCGTGGCCGTCAACTGCAGGCGGCGCTGGATTATCTGACCACCGA<br>CAGCACCGTGCGTGATCGTATTGACCCGAACCGTATGGCGGTTATGGGTCACAGCATGGGCGGTGGC<br>GGTGCGCTGAGCGCGGCGGCGAACAACCCGAGCCTGAAAGCGGCGATTCCGCTGCAGCCGTGGCAC<br>ACCCGTAAAGATTGGAGCAGCGTGCGTGTTCCGACCCTGATCGTGGGCGCGGAACTGGACACCATTGC<br>GCCGGTTAGCAGCCATTCGGAGGCGTTCTACAACAGCCTGCCGAGCAGCCTGCCGAAGGCGTATATG<br>GAACTGCGTGGTGCGGATCACACGGTTAGCAACACCCCGAACACCACCACCGCGAAGTACAGCATCGC<br>GTGGCTGAAACGTTTCGTTGACGATGACACCCGTTATGAGCAATTTCTGTGCCCGGCGCCGGATGACC<br>CGGCGATTAGCGAATACCGTAGCACCTGCCCGTTTCTCGAGCACCACCACCACCACCACTGA	MANPYQRGPDPTESSIEAVRGP<br>FAVAQTTVSRLDASGFGGGTIY<br>YPTDTSQGTFGAVAISPGFTAG<br>QSSIAWLGPRIASQGFVVITIDTI<br>SRLDQPDSRGRQLQAALDYLTT<br>DSTVRDRIDPNRMAVMGHSMG<br>GGGALSAAANNPSLKAAIPLQP<br>WHTRKDWSSVRVPTLIVGAELD<br>TIAPVSSHSEAFYNSLPSSLPKA<br>YMELRGADHTVSNTPNTTTAKY<br>SIAWLKRFVDDDTRYEQFLCPA<br>PDDPAISEYRSTCPFLEHHHHH<br>H
**R4M4 (=R2M2-P155G+A186S+D208S+L104Q)**	ATGGCGAACCCGTATCAACGTGGTCCGGATCCGACCGAGAGCAGCATTGAAGCGGTGCGTGGTCCGTT<br>TGCGGTGGCGCAAACCACCGTTAGCCGTCTGGATGCGAGCGGCTTTGGTGGCGGTACCATCTACTATC<br>CGACCGACACCAGCCAGGGTACCTTTGGTGCGGTTGCGATTAGCCCGGGCTTTACCGCGGGTCAGAG<br>CAGCATTGCGTGGCTGGGTCCGCGTATTGCGAGCCAAGGTTTCGTGGTTATCACCATTGACACCATCA<br>GCCGTCTGGATCAGCCGGACAGCCGTGGCCGTCAACTGCAGGCGGCGCTGGATTATCTGACCACCGA<br>CAGCACCGTGCGTGATCGTATTGACCCGAACCGTATGGCGGTTATGGGTCACAGCATGGGCGGTGGC<br>GGTGCGCTGAGCGCGGCGGCGAACAACCCGAGCCTGAAAGCGGCGATTCCGCTGCAGGGGTGGCAC<br>ACCCGTAAAGATTGGAGCAGCGTGCGTGTTCCGACCCTGATCGTGGGCGCGCAACTGGACACCATTGC<br>GCCGGTTAGCAGCCATTCGGAGGCGTTCTACAACAGCCTGCCGAGCAGCCTGCCGAAGGCGTATATG<br>GAACTGCGTGGTGCGAGTCACCTGGTTAGCAACACCCCGAACACCACCACCGCGAAGTACAGCATCGC<br>GTGGCTGAAACGTTTCGTTGACGATGACACCCGTTATGAGCAATTTCTGTGCCCGGCGCCGGATGACC<br>CGGCGATTAGCGAATACCGTAGCACCTGCCCGTTTCTCGAGCACCACCACCACCACCACTGA	MANPYQRGPDPTESSIEAVRGP<br>FAVAQTTVSRLDASGFGGGTIY<br>YPTDTSQGTFGAVAISPGFTAG<br>QSSIAWLGPRIASQGFVVITIDTI<br>SRLDQPDSRGRQLQAALDYLTT<br>DSTVRDRIDPNRMAVMGHSMG<br>GGGALSAAANNPSLKAAIPLQG<br>WHTRKDWSSVRVPTLIVGAQL<br>DTIAPVSSHSEAFYNSLPSSLPK<br>AYMELRGASHLVSNTPNTTTAK<br>YSIAWLKRFVDDDTRYEQFLCP<br>APDDPAISEYRSTCPFLEHHHH<br>HH
**R4M5 (=R2M2-P155G+A186S+D208S+L104Q+Q175E)**	ATGGCGAACCCGTATCAACGTGGTCCGGATCCGACCGAGAGCAGCATTGAAGCGGTGCGTGGTCCGTT<br>TGCGGTGGCGCAAACCACCGTTAGCCGTCTGGATGCGAGCGGCTTTGGTGGCGGTACCATCTACTATC<br>CGACCGACACCAGCCAGGGTACCTTTGGTGCGGTTGCGATTAGCCCGGGCTTTACCGCGGGTCAGAG<br>CAGCATTGCGTGGCTGGGTCCGCGTATTGCGAGCCAAGGTTTCGTGGTTATCACCATTGACACCATCA<br>GCCGTCTGGATCAGCCGGACAGCCGTGGCCGTCAACTGCAGGCGGCGCTGGATTATCTGACCACCGA<br>CAGCACCGTGCGTGATCGTATTGACCCGAACCGTATGGCGGTTATGGGTCACAGCATGGGCGGTGGC<br>GGTGCGCTGAGCGCGGCGGCGAACAACCCGAGCCTGAAAGCGGCGATTCCGCTGCAGGGGTGGCAC<br>ACCCGTAAAGATTGGAGCAGCGTGCGTGTTCCGACCCTGATCGTGGGCGCGGAACTGGACACCATTGC<br>GCCGGTTAGCAGCCATTCGGAGGCGTTCTACAACAGCCTGCCGAGCAGCCTGCCGAAGGCGTATATG<br>GAACTGCGTGGTGCGAGTCACCTGGTTAGCAACACCCCGAACACCACCACCGCGAAGTACAGCATCGC<br>GTGGCTGAAACGTTTCGTTGACGATGACACCCGTTATGAGCAATTTCTGTGCCCGGCGCCGGATGACC<br>CGGCGATTAGCGAATACCGTAGCACCTGCCCGTTTCTCGAGCACCACCACCACCACCACTGA	MANPYQRGPDPTESSIEAVRGP<br>FAVAQTTVSRLDASGFGGGTIY<br>YPTDTSQGTFGAVAISPGFTAG<br>QSSIAWLGPRIASQGFVVITIDTI<br>SRLDQPDSRGRQLQAALDYLTT<br>DSTVRDRIDPNRMAVMGHSMG<br>GGGALSAAANNPSLKAAIPLQG<br>WHTRKDWSSVRVPTLIVGAELD<br>TIAPVSSHSEAFYNSLPSSLPKA<br>YMELRGASHLVSNTPNTTTAKY<br>SIAWLKRFVDDDTRYEQFLCPA<br>PDDPAISEYRSTCPFLEHHHHH<br>H
**R4M6 (=R2M2-P155G+A186S+D208S+L104Q+Q175E+L210T)**	ATGGCGAACCCGTATCAACGTGGTCCGGATCCGACCGAGAGCAGCATTGAAGCGGTGCGTGGTCCGTT<br>TGCGGTGGCGCAAACCACCGTTAGCCGTCTGGATGCGAGCGGCTTTGGTGGCGGTACCATCTACTATC<br>CGACCGACACCAGCCAGGGTACCTTTGGTGCGGTTGCGATTAGCCCGGGCTTTACCGCGGGTCAGAG<br>CAGCATTGCGTGGCTGGGTCCGCGTATTGCGAGCCAAGGTTTCGTGGTTATCACCATTGACACCATCA<br>GCCGTCTGGATCAGCCGGACAGCCGTGGCCGTCAACTGCAGGCGGCGCTGGATTATCTGACCACCGA<br>CAGCACCGTGCGTGATCGTATTGACCCGAACCGTATGGCGGTTATGGGTCACAGCATGGGCGGTGGC<br>GGTGCGCTGAGCGCGGCGGCGAACAACCCGAGCCTGAAAGCGGCGATTCCGCTGCAGGGGTGGCAC<br>ACCCGTAAAGATTGGAGCAGCGTGCGTGTTCCGACCCTGATCGTGGGCGCGGAACTGGACACCATTGC<br>GCCGGTTAGCAGCCATTCGGAGGCGTTCTACAACAGCCTGCCGAGCAGCCTGCCGAAGGCGTATATG<br>GAACTGCGTGGTGCGAGTCACACGGTTAGCAACACCCCGAACACCACCACCGCGAAGTACAGCATCGC<br>GTGGCTGAAACGTTTCGTTGACGATGACACCCGTTATGAGCAATTTCTGTGCCCGGCGCCGGATGACC<br>CGGCGATTAGCGAATACCGTAGCACCTGCCCGTTTCTCGAGCACCACCACCACCACCACTGA	MANPYQRGPDPTESSIEAVRGP<br>FAVAQTTVSRLDASGFGGGTIY<br>YPTDTSQGTFGAVAISPGFTAG<br>QSSIAWLGPRIASQGFVVITIDTI<br>SRLDQPDSRGRQLQAALDYLTT<br>DSTVRDRIDPNRMAVMGHSMG<br>GGGALSAAANNPSLKAAIPLQG<br>WHTRKDWSSVRVPTLIVGAELD<br>TIAPVSSHSEAFYNSLPSSLPKA<br>YMELRGASHTVSNTPNTTTAKY<br>SIAWLKRFVDDDTRYEQFLCPA<br>PDDPAISEYRSTCPFLEHHHHH<br>H
**R4M7 (=R2M2--A186S+D208S/L104Q+Q175E)**	ATGGCGAACCCGTATCAACGTGGTCCGGATCCGACCGAGAGCAGCATTGAAGCGGTGCGTGGTCCGTT<br>TGCGGTGGCGCAAACCACCGTTAGCCGTCTGGATGCGAGCGGCTTTGGTGGCGGTACCATCTACTATC<br>CGACCGACACCAGCCAGGGTACCTTTGGTGCGGTTGCGATTAGCCCGGGCTTTACCGCGGGTCAGAG<br>CAGCATTGCGTGGCTGGGTCCGCGTATTGCGAGCCAAGGTTTCGTGGTTATCACCATTGACACCATCA<br>GCCGTCTGGATCAGCCGGACAGCCGTGGCCGTCAACTGCAGGCGGCGCTGGATTATCTGACCACCGA<br>CAGCACCGTGCGTGATCGTATTGACCCGAACCGTATGGCGGTTATGGGTCACAGCATGGGCGGTGGC<br>GGTGCGCTGAGCGCGGCGGCGAACAACCCGAGCCTGAAAGCGGCGATTCCGCTGCAGCCGTGGCAC<br>ACCCGTAAAGATTGGAGCAGCGTGCGTGTTCCGACCCTGATCGTGGGCGCGGAACTGGACACCATTGC<br>GCCGGTTAGCAGCCATTCGGAGGCGTTCTACAACAGCCTGCCGAGCAGCCTGCCGAAGGCGTATATG<br>GAACTGCGTGGTGCGAGTCACCTGGTTAGCAACACCCCGAACACCACCACCGCGAAGTACAGCATCGC	MANPYQRGPDPTESSIEAVRGP<br>FAVAQTTVSRLDASGFGGGTIY<br>YPTDTSQGTFGAVAISPGFTAG<br>QSSIAWLGPRIASQGFVVITIDTI<br>SRLDQPDSRGRQLQAALDYLTT<br>DSTVRDRIDPNRMAVMGHSMG<br>GGGALSAAANNPSLKAAIPLQP<br>WHTRKDWSSVRVPTLIVGAELD<br>TIAPVSSHSEAFYNSLPSSLPKA<br>YMELRGASHLVSNTPNTTTAKY

	GTGGCTGAAACGTTTCGTTGACGATGACACCCGTTATGAGCAATTTCTGTGCCCGGCGCCGGATGACC<br>CGGCGATTAGCGAATACCGTAGCACCTGCCCGTTTCTCGAGCACCACCACCACCACCACTGA	SIAWLKRFVDDDTRYEQFLCPA<br>PDDPAISEYRSTCPFLEHHHHH<br>H
**R4M8 (R2M2-A186S+D208S+L104Q+Q175E+L210T)**	ATGGCGAACCCGTATCAACGTGGTCCGGATCCGACCGAGAGCAGCATTGAAGCGGTGCGTGGTCCGTT<br>TGCGGTGGCGCAAACCACCGTTAGCCGTCTGGATGCGAGCGGCTTTGGTGGCGGTACCATCTACTATC<br>CGACCGACACCAGCCAGGGTACCTTTGGTGCGGTTGCGATTAGCCCGGGCTTTACCGCGGGTCAGAG<br>CAGCATTGCGTGGCTGGGTCCGCGTATTGCGAGCCAAGGTTTCGTGGTTATCACCATTGACACCATCA<br>GCCGTCTGGATCAGCCGGACAGCCGTGGCCGTCAACTGCAGGCGGCGCTGGATTATCTGACCACCGA<br>CAGCACCGTGCGTGATCGTATTGACCCGAACCGTATGGCGGTTATGGGTCACAGCATGGGCGGTGGC<br>GGTGCGCTGAGCGCGGCGGCGAACAACCCGAGCCTGAAAGCGGCGATTCCGCTGCAGCCGTGGCAC<br>ACCCGTAAAGATTGGAGCAGCGTGCGTGTTCCGACCCTGATCGTGGGCGCGGAACTGGACACCATTGC<br>GCCGGTTAGCAGCCATTCGGAGGCGTTCTACAACAGCCTGCCGAGCAGCCTGCCGAAGGCGTATATG<br>GAACTGCGTGGTGCGAGTCACACGGTTAGCAACACCCCGAACACCACCACCGCGAAGTACAGCATCGC<br>GTGGCTGAAACGTTTCGTTGACGATGACACCCGTTATGAGCAATTTCTGTGCCCGGCGCCGGATGACC<br>CGGCGATTAGCGAATACCGTAGCACCTGCCCGTTTCTCGAGCACCACCACCACCACCACTGA	MANPYQRGPDPTESSIEAVRGP<br>FAVAQTTVSRLDASGFGGGTIY<br>YPTDTSQGTFGAVAISPGFTAG<br>QSSIAWLGPRIASQGFVVITIDTI<br>SRLDQPDSRGRQLQAALDYLTT<br>DSTVRDRIDPNRMAVMGHSMG<br>GGGALSAAANNPSLKAAIPLQP<br>WHTRKDWSSVRVPTLIVGAELD<br>TIAPVSSHSEAFYNSLPSSLPKA<br>YMELRGASHTVSNTPNTTTAKY<br>SIAWLKRFVDDDTRYEQFLCPA<br>PDDPAISEYRSTCPFLEHHHHH<br>H
**R4M11 (=R2M2-A186S+L104Q+Q175E+L210T+K148E+D233K)**	ATGGCGAATCCGTATCAACGGGGGCCAGACCCTACTGAGAGTTCCATCGAGGCGGTGAGAGGCCCTTT<br>CGCAGTAGCCCAGACCACAGTGAGTCGCTTAGACGCCTCTGGTTTCGGGGGTGGTACGATCTATTATC<br>CAACTGACACATCACAAGGCACGTTCGGAGCAGTGGCTATCAGCCCAGGATTCACAGCAGGGCAGTCT<br>TCTATCGCCTGGCTTGGCCCCCGCATAGCGAGCCAGGGGTTTGTAGTAATCACCATCGATACGATTTCG<br>CGTTTAGACCAGCCAGATAGCCGTGGTCGCCAGTTACAAGCAGCGTTGGATTATTTAACAACGGACTCT<br>ACCGTTCGGGACCGGATAGATCCCAATCGTATGGCAGTGATGGGGCATTCCATGGGAGGGGGAGGAG<br>CACTGTCCGCTGCAGCAAATAATCCCAGCTTAGAAGCTGCCATTCCCTTACAGCCGTGGCACACCCGTA<br>AAGATTGGTCCTCCGTCCGGGTTCCCACGTTAATTGTGGGAGCTGAATTAGATACCATCGCGCCAGTGT<br>CCTCACATTCGGAAGCCTTTTACAACTCGCTGCCCAGTTCGTTACCAAAGGCATACATGGAATTACGCG<br>GAGCAGATCATACCGTTTCCAACACTCCTAACACTACCACTGCGAAATACAGTATAGCCTGGTTAAAGC<br>GTTTCGTAGACAAAGATACTCGTTACGAACAGTTCCTTTGCCCCGCTCCAGATGACCCTGCCATCTCTG<br>AATACCGTAGCACCTGTCCATTTCTCGAGCACCACCACCACCACTGA	MANPYQRGPDPTESSIEAVRGP<br>FAVAQTTVSRLDASGFGGGTIY<br>YPTDTSQGTFGAVAISPGFTAG<br>QSSIAWLGPRIASQGFVVITIDTI<br>SRLDQPDSRGRQLQAALDYLTT<br>DSTVRDRIDPNRMAVMGHSMG<br>GGGALSAAANNPSLEAAIPLQP<br>WHTRKDWSSVRVPTLIVGAELD<br>TIAPVSSHSEAFYNSLPSSLPKA<br>YMELRGADHTVSNTPNTTTAKY<br>SIAWLKRFVDKDTRYEQFLCPA<br>PDDPAISEYRSTCPFLEHHHHH<br>H
**R4M12 (=R2M2-P155G+A186S+D208S+L104Q+Q175E+L210T+K148E+D233K)**	ATGGCGAATCCGTATCAACGGGGGCCAGACCCTACTGAGAGTTCCATCGAGGCGGTGAGAGGCCCTTT<br>CGCAGTAGCCCAGACCACAGTGAGTCGCTTAGACGCCTCTGGTTTCGGGGGTGGTACGATCTATTATC<br>CAACTGACACATCACAAGGCACGTTCGGAGCAGTGGCTATCAGCCCAGGATTCACAGCAGGGCAGTCT<br>TCTATCGCCTGGCTTGGCCCCCGCATAGCGAGCCAGGGGTTTGTAGTAATCACCATCGATACGATTTCG<br>CGTTTAGACCAGCCAGATAGCCGTGGTCGCCAGTTACAAGCAGCGTTGGATTATTTAACAACGGACTCT<br>ACCGTTCGGGACCGGATAGATCCCAATCGTATGGCAGTGATGGGGCATTCCATGGGAGGGGGAGGAG<br>CACTGTCCGCTGCAGCAAATAATCCCAGCTTAGAAGCTGCCATTCCCTTACAGGGATGGCACACCCGTA<br>AAGATTGGTCCTCCGTCCGGGTTCCCACGTTAATTGTGGGAGCTGAATTAGATACCATCGCGCCAGTGT<br>CCTCACATTCGGAAGCCTTTTACAACTCGCTGCCCAGTTCGTTACCAAAGGCATACATGGAATTACGCG<br>GAGCAAGTCATACCGTTTCCAACACTCCTAACACTACCACTGCGAAATACAGTATAGCCTGGTTAAAGC<br>GTTTCGTAGACAAAGATACTCGTTACGAACAGTTCCTTTGCCCCGCTCCAGATGACCCTGCCATCTCTG<br>AATACCGTAGCACCTGTCCATTTCTCGAGCACCACCACCACCACCACTGA	MANPYQRGPDPTESSIEAVRGP<br>FAVAQTTVSRLDASGFGGGTIY<br>YPTDTSQGTFGAVAISPGFTAG<br>QSSIAWLGPRIASQGFVVITIDTI<br>SRLDQPDSRGRQLQAALDYLTT<br>DSTVRDRIDPNRMAVMGHSMG<br>GGGALSAAANNPSLEAAIPLQG<br>WHTRKDWSSVRVPTLIVGAELD<br>TIAPVSSHSEAFYNSLPSSLPKA<br>YMELRGASHTVSNTPNTTTAKY<br>SIAWLKRFVDKDTRYEQFLCPA<br>PDDPAISEYRSTCPFLEHHHHH<br>H
**R4M9 (=R4M6+L93F+Q95G)**	ATGGCGAACCCGTATCAACGTGGTCCGGATCCGACCGAGAGCAGCATTGAAGCGGTGCGTGGTCCGTT<br>TGCGGTGGCGCAAACCACCGTTAGCCGTCTGGATGCGAGCGGCTTTGGTGGCGGTACCATCTACTATC<br>CGACCGACACCAGCCAGGGTACCTTTGGTGCGGTTGCGATTAGCCCGGGCTTTACCGCGGGTCAGAG<br>CAGCATTGCGTGGCTGGGTCCGCGTATTGCGAGCCAAGGTTTCGTGGTTATCACCATTGACACCATCA<br>GCCGTTTTGATGGGCCGGACAGCCGTGGCCGTCAACTGCAGGCGGCGCTGGATTATCTGACCACCGA<br>CAGCACCGTGCGTGATCGTATTGACCCGAACCGTATGGCGGTTATGGGTCACAGCATGGGCGGTGGC<br>GGTGCGCTGAGCGCGGCGGCGAACAACCCGAGCCTGAAAGCGGCGATTCCGCTGCAGGGGTGGCAC<br>ACCCGTAAAGATTGGAGCAGCGTGCGTGTTCCGACCCTGATCGTGGGCGCGGAACTGGACACCATTGC<br>GCCGGTTAGCAGCCATTCGGAGGCGTTCTACAACAGCCTGCCGAGCAGCCTGCCGAAGGCGTATATG<br>GAACTGCGTGGTGCGAGTCACACGGTTAGCAACACCCCGAACACCACCACCGCGAAGTACAGCATCGC<br>GTGGCTGAAACGTTTCGTTGACGATGACACCCGTTATGAGCAATTTCTGTGCCCGGCGCCGGATGACC<br>CGGCGATTAGCGAATACCGTAGCACCTGCCCGTTTCTCGAGCACCACCACCACCACCACTGA	MANPYQRGPDPTESSIEAVRGP<br>FAVAQTTVSRLDASGFGGGTIY<br>YPTDTSQGTFGAVAISPGFTAG<br>QSSIAWLGPRIASQGFVVITIDTI<br>SRFDGPDSRGRQLQAALDYLTT<br>DSTVRDRIDPNRMAVMGHSMG<br>GGGALSAAANNPSLKAAIPLQG<br>WHTRKDWSSVRVPTLIVGAELD<br>TIAPVSSHSEAFYNSLPSSLPKA<br>YMELRGASHTVSNTPNTTTAKY<br>SIAWLKRFVDDDTRYEQFLCPA<br>PDDPAISEYRSTCPFLEHHHHH<br>H
**R4M10 (=R4M6+L93F+Q95Y**	ATGGCGAACCCGTATCAACGTGGTCCGGATCCGACCGAGAGCAGCATTGAAGCGGTGCGTGGTCCGTT<br>TGCGGTGGCGCAAACCACCGTTAGCCGTCTGGATGCGAGCGGCTTTGGTGGCGGTACCATCTACTATC<br>CGACCGACACCAGCCAGGGTACCTTTGGTGCGGTTGCGATTAGCCCGGGCTTTACCGCGGGTCAGAG<br>CAGCATTGCGTGGCTGGGTCCGCGTATTGCGAGCCAAGGTTTCGTGGTTATCACCATTGACACCATCA<br>GCCGTTTTGATTATCCGGACAGCCGTGGCCGTCAACTGCAGGCGGCGCTGGATTATCTGACCACCGAC<br>AGCACCGTGCGTGATCGTATTGACCCGAACCGTATGGCGGTTATGGGTCACAGCATGGGCGGTGGCG<br>GTGCGCTGAGCGCGGCGGCGAACAACCCGAGCCTGAAAGCGGCGATTCCGCTGCAGGGGTGGCACA<br>CCCGTAAAGATTGGAGCAGCGTGCGTGTTCCGACCCTGATCGTGGGCGCGGAACTGGACACCATTGC<br>GCCGGTTAGCAGCCATTCGGAGGCGTTCTACAACAGCCTGCCGAGCAGCCTGCCGAAGGCGTATATG<br>GAACTGCGTGGTGCGAGTCACACGGTTAGCAACACCCCGAACACCACCACCGCGAAGTACAGCATCGC<br>GTGGCTGAAACGTTTCGTTGACGATGACACCCGTTATGAGCAATTTCTGTGCCCGGCGCCGGATGACC<br>CGGCGATTAGCGAATACCGTAGCACCTGCCCGTTTCTCGAGCACCACCACCACCACCACTGA	MANPYQRGPDPTESSIEAVRGP<br>FAVAQTTVSRLDASGFGGGTIY<br>YPTDTSQGTFGAVAISPGFTAG<br>QSSIAWLGPRIASQGFVVITIDTI<br>SRFDYPDSRGRQLQAALDYLTT<br>DSTVRDRIDPNRMAVMGHSMG<br>GGGALSAAANNPSLKAAIPLQG<br>WHTRKDWSSVRVPTLIVGAELD<br>TIAPVSSHSEAFYNSLPSSLPKA<br>YMELRGASHTVSNTPNTTTAKY<br>SIAWLKRFVDDDTRYEQFLCPA<br>PDDPAISEYRSTCPFLEHHHHH<br>H
**R4M13 (=R4M8+L93F+Q95G)**	ATGGCGAACCCGTACCAGCGGGGGCCGGATCCCACAGAGTCGAGCATTGAGGCCGTAAGAGGACCCT<br>TTGCCGTTGCCCAGACGACGGTGTCGCGGCTTGACGCGAGTGGCTTCGGCGGTGGCACTATCTACTAT<br>CCCACCGACACTAGCCAAGGTACATTCGGGGCAGTGGCAATTTCACCCGGATTTACGGCGGGCCAATC<br>TTCAATCGCATGGTTGGGGCCAAGAATTGCGAGCCAGGGCTTCGTTGTCATAACTATTGATACGATCTC<br>TCGTTTCGATGGTCCAGATAGTCGTGGTCGTCAATTACAGGCAGCCTTAGATTATTTGACCACTGATTCG<br>ACCGTGCGCGATCGTATAGATCCAAACAGAATGGCTGTCATGGGGCACAGTATGGGCGGTGGTGGAGC<br>ATTGTCCGCCGCCGCTAACAACCCTTCCTTGAAGGCAGCTATTCCGTTACAGCCCTGGCATACACGTAA<br>AGACTGGAGTTCTGTGCGCGTTCCGACGTTAATTGTGGGAGCTGAACTGGACACTATCGCCCCGGTAT<br>CCTCGCACTCAGAAGCATTTTACAATTCCTTGCCATCATCGTTACCAAAGGCTTACATGGAGCTTAGAGG<br>GGCCAGTCACACTGTCTCCAACACTCCCAATACTACTACTGCTAAATACAGCATTGCATGGCTTAAACGC	MANPYQRGPDPTESSIEAVRGP<br>FAVAQTTVSRLDASGFGGGTIY<br>YPTDTSQGTFGAVAISPGFTAG<br>QSSIAWLGPRIASQGFVVITIDTI<br>SRFDGPDSRGRQLQAALDYLTT<br>DSTVRDRIDPNRMAVMGHSMG<br>GGGALSAAANNPSLKAAIPLQP<br>WHTRKDWSSVRVPTLIVGAELD<br>TIAPVSSHSEAFYNSLPSSLPKA<br>YMELRGASHTVSNTPNTTTAKY<br>SIAWLKRFVDDDTRYEQFLCPA

	TTCGTAGATGATGACACGCGCTACGAACAGTTCCTGTGCCCTGCCCCCGACGATCCGGCGATCTCTGA<br>GTACCGGAGTACTTGCCCATTTCTCGAGCACCACCACCACCACCACTGA	PDDPAISEYRSTCPFLEHHHHH<br>H
**R4M14 (=R4M8+L93F+Q95Y)**	ATGGCGAACCCGTACCAGCGGGGGCCGGATCCCACAGAGTCGAGCATTGAGGCCGTAAGAGGACCCT<br>TTGCCGTTGCCCAGACGACGGTGTCGCGGCTTGACGCGAGTGGCTTCGGCGGTGGCACTATCTACTAT<br>CCCACCGACACTAGCCAAGGTACATTCGGGGCAGTGGCAATTTCACCCGGATTTACGGCGGGCCAATC<br>TTCAATCGCATGGTTGGGGCCAAGAATTGCGAGCCAGGGCTTCGTTGTCATAACTATTGATACGATCTC<br>TCGTTTCGATTATCCAGATAGTCGTGGTCGTCAATTACAGGCAGCCTTAGATTATTTGACCACTGATTCG<br>ACCGTGCGCGATCGTATAGATCCAAACAGAATGGCTGTCATGGGGCACAGTATGGGCGGTGGTGGAGC<br>ATTGTCCGCCGCCGCTAACAACCCTTCCTTGAAGGCAGCTATTCCGTTACAGCCCTGGCATACACGTAA<br>AGACTGGAGTTCTGTGCGCGTTCCGACGTTAATTGTGGGAGCTGAACTGGACACTATCGCCCCGGTAT<br>CCTCGCACTCAGAAGCATTTTACAATTCCTTGCCATCATCGTTACCAAAGGCTTACATGGAGCTTAGAGG<br>GGCCAGTCACACTGTCTCCAACACTCCCAATACTACTACTGCTAAATACAGCATTGCATGGCTTAAACGC<br>TTCGTAGATGATGACACGCGCTACGAACAGTTCCTGTGCCCTGCCCCCGACGATCCGGCGATCTCTGA<br>GTACCGGAGTACTTGCCCATTTCTCGAGCACCACCACCACCACCACTGA	MANPYQRGPDPTESSIEAVRGP<br>FAVAQTTVSRLDASGFGGGTIY<br>YPTDTSQGTFGAVAISPGFTAG<br>QSSIAWLGPRIASQGFVVITIDTI<br>SRFDYPDSRGRQLQAALDYLTT<br>DSTVRDRIDPNRMAVMGHSMG<br>GGGALSAAANNPSLKAAIPLQP<br>WHTRKDWSSVRVPTLIVGAELD<br>TIAPVSSHSEAFYNSLPSSLPKA<br>YMELRGASHTVSNTPNTTTAKY<br>SIAWLKRFVDDDTRYEQFLCPA<br>PDDPAISEYRSTCPFLEHHHHH<br>H
**R4M15 (=R4M12+L93F+Q95Y)**	ATGGCGAATCCGTATCAACGGGGGCCAGACCCTACTGAGAGTTCCATCGAGGCGGTGAGAGGCCCTTT<br>CGCAGTAGCCCAGACCACAGTGAGTCGCTTAGACGCCTCTGGTTTCGGGGGTGGTACGATCTATTATC<br>CAACTGACACATCACAAGGCACGTTCGGAGCAGTGGCTATCAGCCCAGGATTCACAGCAGGGCAGTCT<br>TCTATCGCCTGGCTTGGCCCCCGCATAGCGAGCCAGGGGTTTGTAGTAATCACCATCGATACGATTTCG<br>CGTTTCGACTATCCAGATAGCCGTGGTCGCCAGTTACAAGCAGCGTTGGATTATTTAACAACGGACTCT<br>ACCGTTCGGGACCGGATAGATCCCAATCGTATGGCAGTGATGGGGCATTCCATGGGAGGGGGAGGAG<br>CACTGTCCGCTGCAGCAAATAATCCCAGCTTAGAAGCTGCCATTCCCTTACAGGGATGGCACACCCGTA<br>AAGATTGGTCCTCCGTCCGGGTTCCCACGTTAATTGTGGGAGCTGAATTAGATACCATCGCGCCAGTGT<br>CCTCACATTCGGAAGCCTTTTACAACTCGCTGCCCAGTTCGTTACCAAAGGCATACATGGAATTACGCG<br>GAGCAAGTCATACCGTTTCCAACACTCCTAACACTACCACTGCGAAATACAGTATAGCCTGGTTAAAGC<br>GTTTCGTAGACAAAGATACTCGTTACGAACAGTTCCTTTGCCCCGCTCCAGATGACCCTGCCATCTCTG<br>AATACCGTAGCACCTGTCCATTTCTCGAGCACCACCACCACCACCACTGA	MANPYQRGPDPTESSIEAVRGP<br>FAVAQTTVSRLDASGFGGGTIY<br>YPTDTSQGTFGAVAISPGFTAG<br>QSSIAWLGPRIASQGFVVITIDTI<br>SRFDYPDSRGRQLQAALDYLTT<br>DSTVRDRIDPNRMAVMGHSMG<br>GGGALSAAANNPSLEAAIPLQG<br>WHTRKDWSSVRVPTLIVGAELD<br>TIAPVSSHSEAFYNSLPSSLPKA<br>YMELRGASHTVSNTPNTTTAKY<br>SIAWLKRFVDKDTRYEQFLCPA<br>PDDPAISEYRSTCPFLEHHHHH<br>H
**ICCG**	ATGTCAAATCCCTACCAACGTGGGCCAAACCCTACGCGGAGTGCTCTGACAGCCGATGGCCCTTCTC<br>GGTTGCAACGTACACAGTATCACGTTTGAGCGTATCAGGATTCGGTGGTGGGGTGATATACTACCCCAC<br>AGGGACCTCGCTGACTTTCGGAGGGATAGCGATGTCTCCCGGGTACACGGCAGATGCCAGTTCCCTTG<br>CATGGCTTGGCCGCCGGCTTGCATCCCACGGTTTCGTTGTCTTAGTCATCAATACAAATTCTAGATTTGA<br>TGGCCCGGACAGTCGGGCTTCCCAGCTTAGCGCAGCTCTTAATTACTTGCGCACTTCGAGTCCGAGCG<br>CTGTGCGCGCGCGGTTGGATGCCAATCGCTTGGCAGTAGCAGGACATAGTATGGGTGGGGGTGGTAC<br>TCTTAGAATTGCTGAACAAAACCCCTCCTTAAAAGCCGCGGTTCCGCTGACGCCCTGGCACACGGACAA<br>GACTTTTAATACCAGCGTACCTGTTTTGATAGTAGGGGCGGAAGCTGACACTGTCGCCCCGGTCTCACA<br>ACATGCTATCCCATTTTATCAAAATCTTCCATCAACAACTCCGAAGGTCTACGTGGAATTATGCAACGCA<br>AGTCACATTGCTCCAAACTCAAACAACGCCGCCATTTCTGTCTATACCATAAGCTGGATGAAACTGTGG<br>GTTGACAACGATACAAGATATAGACAGTTTTTGTGCAACGTGAATGATCCTGCCTTGTGTGATTTTCGTA<br>CTAATAACCGCCATTGTCAGCTCGAGCACCACCACCACCACCACTGA	MSNPYQRGPNPTRSALTADGP<br>FSVATYTVSRLSVSGFGGGVIY<br>YPTGTSLTFGGIAMSPGYTADA<br>SSLAWLGRRLASHGFVVLVINT<br>NSRFDGPDSRASQLSAALNYLR<br>TSSPSAVRARLDANRLAVAGHS<br>MGGGGTLRIAEQNPSLKAAVPL<br>TPWHTDKTFNTSVPVLIVGAEA<br>DTVAPVSQHAIPFYQNLPSTTP<br>KVYVELCNASHIAPNSNNAAISV<br>YTISWMKLWVDNDTRYRQFLC<br>NVNDPALCDFRTNNRHCQLEH<br>HHHHH
**HotPETase**	ATGCAGACTAACCCGTATGCCAGAGGCCCGAACCCAACCGCCGCAAGTTTAGAGGCTTCAGCAGGCCC<br>TTTTACAGTCCGGAGTTTCACTGTGGCTCGTCCTGTGGGCTATGGGGCGGGAACGGTTTACTACCCAAC<br>AAATGCAGGGGGAACTGTCGGCGCAATAGCCATAGTCCCTGGCTACACTGCCACGCAATCCAGTATAA<br>ATTGGTGGGGCCCTCGTCTTGCTAGTCATGGCTTTGTGGTGATAACGATTGACACTAACTCAACGTTAG<br>ACAAACCGGAGAGCCGGTCGAGCCAACAAATGGCAGCACTGCGTCAGGTTGCGTCGTTGAACGGTACA<br>AGCAGTTCGCCTATATATGGCAAGGTGGATACAGCTCGTGGTGGCGTGATGGGATGGAGTATGGGAGG<br>CGGAGGGTCGTTAATCTCAGCCGCTAACAATCCGAGCCTGAAAGCCGCCGCAGTCATGGCCCCCTGGC<br>ATAGCTCTACTAATTTCTCAAGCGTCACGGTGCCTACCCTTATATTCGCTTGTGAGAATGATCGGATTGC<br>ACCTGTTAAGGAATATGCATTACCTATCTATGACTCCATGTCCCTGAACGCGAAGCAATTTCTTGAGATT<br>TGCGGCGGTAGTCACTCATGCGCCTGCAGCGGCAATAGCAACCAGGCTCTTATCGGTATGAAAGGGGT<br>TGCTTGGATGAAAAGATTCATGGACAATGATACCCGTTACTCTCAGTTTGCTTGCGAGAATCCAAACTCA<br>ACCGCTGTGTGCGATTTCCGGACCGCTAACTGTTCTCTCGAGCACCACCACCACCACCACTGA	MQTNPYARGPNPTAASLEASA<br>GPFTVRSFTVARPVGYGAGTVY<br>YPTNAGGTVGAIAIVPGYTATQS<br>SINWWGPRLASHGFVVITIDTNS<br>TLDKPESRSSQQMAALRQVASL<br>NGTSSSPIYGKVDTARGGVMG<br>WSMGGGGSLISAANNPSLKAA<br>AVMAPWHSSTNFSSVTVPTLIF<br>ACENDRIAPVKEYALPIYDSMSL<br>NAKQFLEICGGSHSCACSGNSN<br>QALIGMKGVAWMKRFMDNDTR<br>YSQFACENPNSTAVCDFRTAN<br>CSLEHHHHHH

Supplementary Table 3. Primers used in this study.

Name	Nucleotide Sequence (5' -> 3')
PHL7-G155T_forward	GCTGCAGACCTGGCACACCCGGAAG
PHL7-G155T_reverse	GTGCCAGGTCTGCAGCGGGATGGC
PHL7-G155A_forward	GCTGCAGGCCTGGCACACCCGGAAG
PHL7-G155A_reverse	GTGCCAGGCCTGCAGCGGGATGGC
PHL7-G155S_forward	GCTGCAGAGCTGGCACACCCGGAAG
PHL7-G155S_reverse	GTGCCAGCTCTGCAGCGGGATGGC
PHL7-G155P_forward	GCTGCAGCCCTGGCACACCCGGAAG
PHL7-G155P_reverse	GTGCCAGGGCTGCAGCGGGATGGC
PHL7-R2M2-Q6E_forward	GTATGAACGTGGTCCGGATCCGAC
PHL7-R2M2-Q6E_reverse	CGGACCACGTTCATACGGGTTCGC
PHL7-R2M2-S68E_forward	GTCAGGAGAGCATTGCGTGGCTGG
PHL7-R2M2-S68E_reverse	TGCTCTCCTGACCCGCGGTAAAGC
PHL7-R2M2-P155G_forward	GCAGGGGTGGCACACCCGTAAAGA
PHL7-R2M2-P155G_reverse	GCCACCCCTGCAGCGGAATCGC
PHL7-R2M2-I171V_forward	CCTGGTCGTGGGCGCG
PHL7-R2M2-I171V_reverse	CCACGACCAGGGTCGGAACACG
PHL7-R2M2-L104Q_forward	CCGTCAACTGCAGGCGGCGCTGGATTATC
PHL7-R2M2-L104Q_reverse	CCGCCTGCAGTTGACGGCCACGGCTG
PHL7-R2M2-K148E_forward	CAACCCGAGCCTGGAAGCGGCGATTC
PHL7-R2M2-K148E_reverse	GCTTCCAGGCTCGGGTTGTTCGCCGC
PHL7-R2M2-Q175E_forward	GGGCGCGGAACTGGACACCATTGC
PHL7-R2M2-Q175E_reverse	GTCCAGTTCCGCGCCCACGATCAG
PHL7-R2M2-A186S_forward	CAGCCATTCGGAGGCGTTCTACAAC
PHL7-R2M2-A186S_reverse	GCCTCCGAATGGCTGCTAACCGG
PHL7-R2M2-P198D_forward	CCTGGACAAGGCGTATATGGAACTGCGTGGTGC
PHL7-R2M2-P198D_reverse	CATATACGCCTTGTCCAGGCTGCTCGGCAGG
PHL7-R2M2-P198D-S196D_forward	CTGCCGAGCGACCTGGACAAGGCG
PHL7-R2M2-P198D-S196D_reverse	CCAGGTCGCTCGGCAGGCTGTTGTAG
PHL7-R2M2-D208S_forward	GTGGTGCGAGTCACCTGGTTAGCAAC
PHL7-R2M2-D208S_reverse	CAGGTGACTCGCACCACGCAGTTCC
PHL7-R2M2-L210T_forward	GCGGATCACACGGTTAGCAACACCC
PHL7-R2M2-L210T_reverse	CTAACCGTGTGATCCGCACCACGCAG
PHL7-R2M2-D208S-L210T_forward	GCGAGTCACACGGTTAGCAACACCC
PHL7-R2M2-D208S-L210T_reverse	CTAACCGTGTGACTCGCACCACGCAG
PHL7-R2M2-D233K_forward	GTTGACAAGGACACCCGTTATGAGC
PHL7-R2M2-D233K_reverse	CGGGTGTCCTTGTCAACGAAACG
PHL7-R4M6/R4M8-Q95G_forward	GGATGGGCCGGACAGCCGT
PHL7-R4M6/R4M8-Q95G_reverse	GGCCCATCCAGACGGCTGATGGT
PHL7-Q95Y_foward	GCCTCGACTATCCCGACAGCCGGG
PHL7-Q95Y_reverse	CTGTCGGGATAGTCGAGGCGCGTG
PHL7- L93F_foward	GATCACGCGCTTTGACCAGCCCGACAGC
PHL7- L93F_reverse	GGGCTGGTCAAAGCGCGTGATCGTGTC
PHL7- D233K_foward	GTTCGTCGACAAAGACCTCCGCTACGAGCAG
PHL7-D233K_reverse	GCGGAGGTCTTTGTCGACGAACCGCTTGAGC
PHL7F93-Q95Y_forward	CGCTTTGACTATCCCGACAGCCGGG
PHL7F93-Q95Y_reverse	CTGTCGGGATAGTCAAAGCGCGTGATCG
PHL7-Q95G_forward	CCTCGACGGCCCCGACAGCCGGG
PHL7-Q95G_reverse	CTGTCGGGGCCGTCGAGGCGCGTG
PHL7Q95G-L93F_forward	CACGCGCTTTGACGGCCCCGA
PHL7Q95G-L93F_reverse	GCCGTCAAAGCGCGTGATCGTGTC
PHL7-Q175E_forward	GGGGCCGAACTCGACACCATCGCGC
PHL7-Q175E_reverse	TGTCGAGTTCGGCCCCGACCACC
PHL7-L210T_forward	CCACACCGTGTCGAACACGCCCGA
PHL7-L210T_reverse	GACACGGTGTGGCTGGCCCCG

Supplementary Table 4. Data collection and refinement statistics for PHL7 variant structures.

**Compound**	**PHL7_R2M2**	**PHL7_R2M2-P155G**
**PDB entry ID**	**9QNM**	**9QT8**
Data collection		
Source	DESY EMBL P13	DESY EMBL P13
Wavelength (Å)	0.9762	0.9763
Resolution (Å)	63.10-1.12 (1.24-1.12)	62.98-1.21 (1.35-1.21)
Resolution aniso (Å)	1.73, 1.23, 1.12	1.69, 1.34, 1.21
Space group	$P2_1$	$P2_1$
Unit cell dimensions (Å;°)	52.26, 74.10, 63.60; 90, 97.14, 90	52.83, 74.25, 63.55; 90, 97.71, 90
Unique reflections	99522 (4977)	87694 (4385)
Multiplicity	6.7 (6.5)	7.0 (7.0)
Completeness (%)* spherical/ellipsoidal	53.8 (10.3) / 90.8 (80.3)	59.5 (11.0) / 91.6 (71.9)
Mean I/σ(I)	10.6 (1.6)	11.3 (1.4)
R-meas	0.101 (1.235)	0.094 (1.316)
R-merge	0.094 (1.137)	0.087 (1.219)
R-pim	0.038 (0.475)	0.035 (0.492)
$CC_{1/2}$	0.999 (0.578)	0.999 (0.580)
Wilson B ($Å^2$)	9.86	11.00
Refinement		
Resolution (Å)	63.10-1.12 (1.13-1.12)	31.49-1.21 (1.24-1.21)
R-work	0.1721 (0.2901)	0.1429 (0.2578)
R-free	0.2025 (0.5621)	0.1778 (0.2677)
Number of non-hydrogen atoms, B-value ($Å^2$)		
Protein	4068, 11.96	4117, 13.15
Heterogen	26, 19.92	13, 42.94
Solvent	782, 25.99	893, 27.70
Rmsd bonds (Å), angles (°)	0.010, 1.025	0.008, 0.909
Ramachandran favored, allowed, outliers (%)	98.45, 1.55, 0.00	98.84, 1.16, 0.00
Rotamer outliers (%)	0.00	0.00
MolProbity clashscore	1.11	1.34

*Anisotropic truncation has been used. The first value refers to the spherical and the second value to the ellipsoidal completeness.

Supplementary Table 4: Continued

**Compound**	**PHL7_R2M2-A186S**	**PHL7_R2M2-P155G-A186S**
**PDB entry ID**	**9QV8**	**9QVA**
Data collection		
Source	DESY EMBL P13	DESY EMBL P13
Wavelength (Å)	0.9763	0.9762
Resolution (Å)	63.26-1.34 (1.44-1.25)	62.95-1.59 (1.75-1.59)
Resolution aniso (Å)	1.86, 1.34, 1.25	2.430, 1.681, 1.587
Space group	$P2_1$	$P2_1$
Unit cell dimensions (Å;°)	52.10, 73.73, 63.76; 90, 97.20, 90	52.61, 74.06, 63.50; 90, 97.57, 90
Unique reflections	69216 (3243)	36912 (1847)
Multiplicity	6.3 (6.4)	7.0 (6.6)
Completeness (%)* spherical/ellipsoidal	64.4 (15.3) / 88.9 (52.6)	56.6 (11.4) / 90.6 (64.4)
Mean I/σ(I)	9.9 (1.6)	6.9 (1.5)
R-meas	0.120 (1.228)	0.230 (1.320)
R-merge	0.110 (1.130)	0.213 (1.217)
R-pim	0.047 (0.474)	0.086 (0.503)
$CC_{1/2}$	0.998 (0.624)	0.994 (0.587)
Wilson B (Å^2)	14.72	11.93
Refinement		
Resolution (Å)	42.74-1.25 (1.31-1.25)	62.95-1.59 (1.61-1.59)
R-work	0.1925 (0.4310)	0.2007 (0.3170)
R-free	0.2240 (0.4388)	0.2461 (0.4048)
Number of non-hydrogen atoms, B-value (Å^2)		
Protein	4086, 19.33	4088, 14.94
Heterogen	26, 53.48	26, 41.72
Solvent	636, 31.79	710, 22.49
Rmsd bonds (Å), angles (°)	0.004, 0.684	0.002, 0.482
Ramachandran favored, allowed, outliers (%)	98.65, 1.35, 0.00	98.07, 1.93, 0.00
Rotamer outliers (%)	0.00	0.44
MolProbity clashscore	0.98	0.98

*Anisotropic truncation has been used. The first value refers to the spherical and the second value to the ellipsoidal completeness.

Supplementary Table 4: Continued

**Compound**	**PHL7_R4M6**	**PHL7_R4M10**	**PHL7_R4M12**
**PDB entry ID**	**9QYA**	**9QYB**	**9QYC**
Data collection			
Source	DESY EMBL P14	DESY EMBL P13	DESY EMBL P13
Wavelength (Å)	0.9763	0.7293	0.9762
Resolution (Å)	62.13-1.53 (1.71-1.53)	69.90-1.10 (1.12-1.10)	52.87-1.16 (1.22-1.16)
Resolution aniso (Å)	1.837, 1.837, 1.529	-	1.164, 1.164, 1.236
Space group	$P6_122$	$P6_122$	$P4_12_12$
Unit cell dimensions (Å;°)	80.38, 80.38, 137.82; 90, 90, 120	80.72, 80.72, 138.04; 90, 90, 120	74.77, 74.77, 106.82; 90, 90, 90
Unique reflections	27315 (1366)	107770 (5279)	93166 (4659)
Multiplicity	38.4 (31.3)	40.3 (41.4)	26.6 (28.3)
Completeness (%)* spherical/ellipsoidal	67.7 (12.3) / 96.0 (79.4)	100.0 (100.0)	89.8 (38.0) / 94.0 (53.2)
Mean I/σ(I)	15.4 (1.5)	9.6 (2.1)	13.1 (1.5)
R-meas	0.250 (3.283)	0.289 (2.754)	0.169 (3.037)
R-merge	0.247 (3.231)	0.282 (2.690)	0.166 (2.983)
R-pim	0.040 (0.575)	0.062 (0.589)	0.033 (0.567)
$CC_{1/2}$	0.999 (0.557)	0.995 (0.581)	0.997 (0.363)
Wilson B ($Å^2$)	20.66	11.89	12.29
Refinement			
Resolution (Å)	62.13-1.53 (1.57-1.53)	38.74-1.10 (1.13-1.10)	32.15-1.16 (1.19-1.16)
R-work	0.1570 (0.2409)	0.1528 (0.2606)	0.1208 (0.2885)
R-free	0.1945 (0.3135)	0.1701 (0.2553)	0.1483 (0.3416)
Number of non-hydrogen atoms, B-value ($Å^2$)			
Protein	1985, 21.98	2051, 14.42	2129, 15.87
Heterogen	2, 31.58	2, 20.40	13, 32.60
Solvent	282, 34.82	303, 26.54	396, 35.06
Rmsd bonds (Å), angles (°)	0.004, 0.726	0.013, 1.187	0.014, 1.469
Ramachandran favored, allowed, outliers (%)	98.83, 1.17, 0.00	98.44, 1.56, 0.00	98.46, 1.54, 0.00
Rotamer outliers (%)	0.46	0.00	0.00
MolProbity clashscore	0.00	0.98	0.47

*Anisotropic truncation has been used. The first value refers to the spherical and the second value to the ellipsoidal completeness.

Supplementary Table 5. MMPBSA-computed interaction energies between enzyme variants and 4xMHET substrates in HREX trajectories. The trajectory from the replica with the unmodified topology is analyzed. Reported is the number of frames used for calculation, not the overall number of frames in trajectories.

	In oxyanion hole			Near oxyanion hole			Other poses		
4xMHET	ΔE, kcal/mol	SEM	N frames	ΔE, kcal/mol	SEM	N frames	ΔE, kcal/mol	SEM	N frames
WT	-40.98	0.04	8576	-43.61	0.05	11077	-41.80	0.05	12501
R4M6	-37.01	0.06	9637	-43.61	0.04	15141	-42.93	0.05	12501
R4M9	-37.56	0.06	9166	-44.14	0.04	10726	-41.86	0.05	12501
R4M10	-37.72	0.06	8182	-45.17	0.05	11794	-44.04	0.05	12501

Supplementary Figures

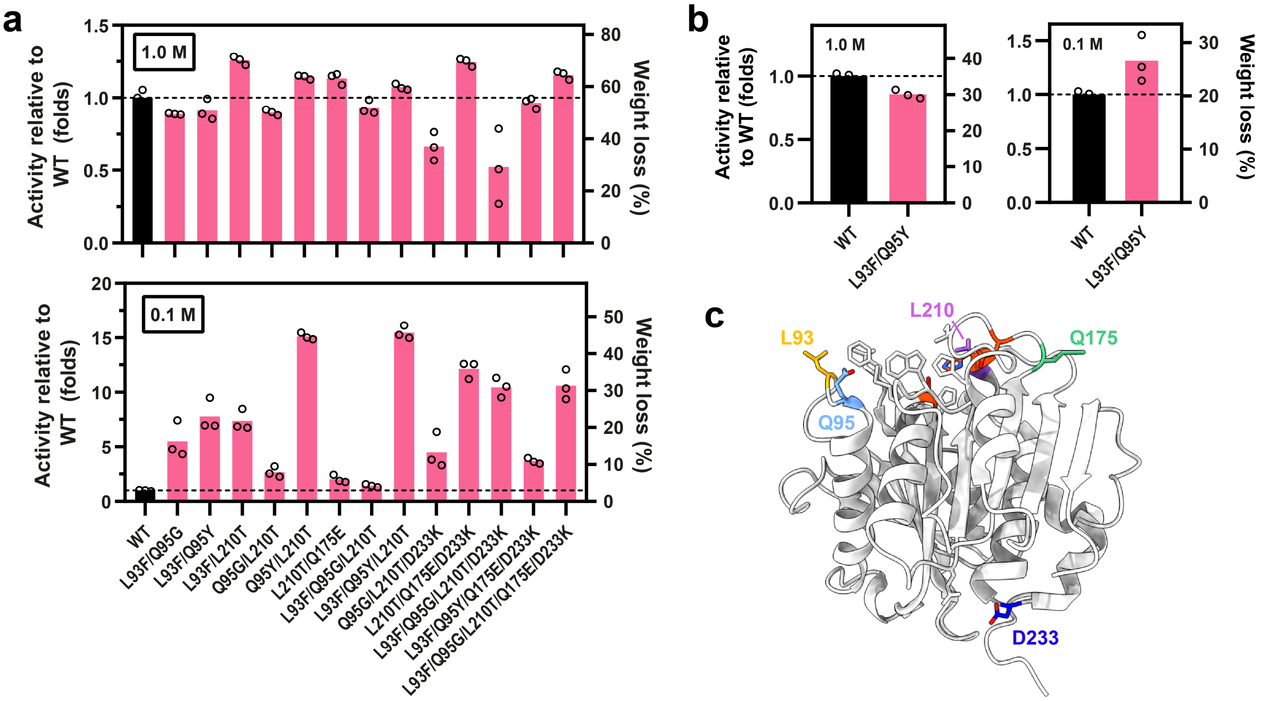

Supplementary Figure 1. PET-hydrolytic activity of PHL7 mutants at 65 °C (**a**) and of mutant L93F/Q95Y at 60 °C (**b**) in 0.1 M and 1.0 M phosphate buffer. All measurements were conducted in triplicates (n = 3 independent experiments). Data represent mean ± standard deviation. **c** Location of mutation sites in the PHL7 structure. The catalytic triad residues (S131, D177, H209) are colored red.

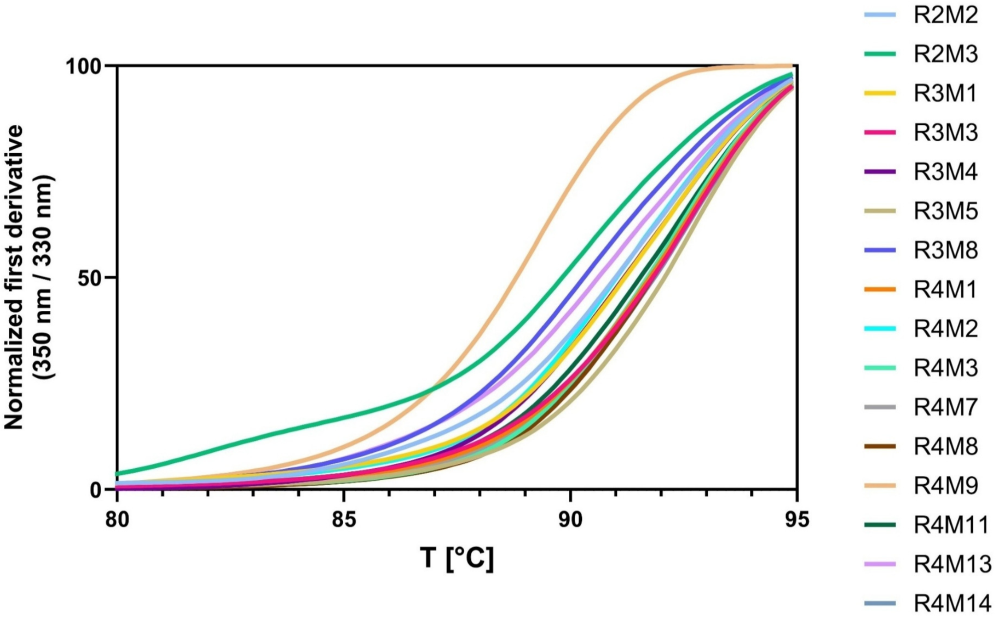

Supplementary Figure 2. Melting curves of the variants that showed a T_m >95 ºC. The graph shows the first derivative of the ratio of the fluorescence measured at 350 and 330 nm by nanoDSF. The measurements were taken in triplicates (n = 3 independent experiments). For clarity, only the mean values are shown.

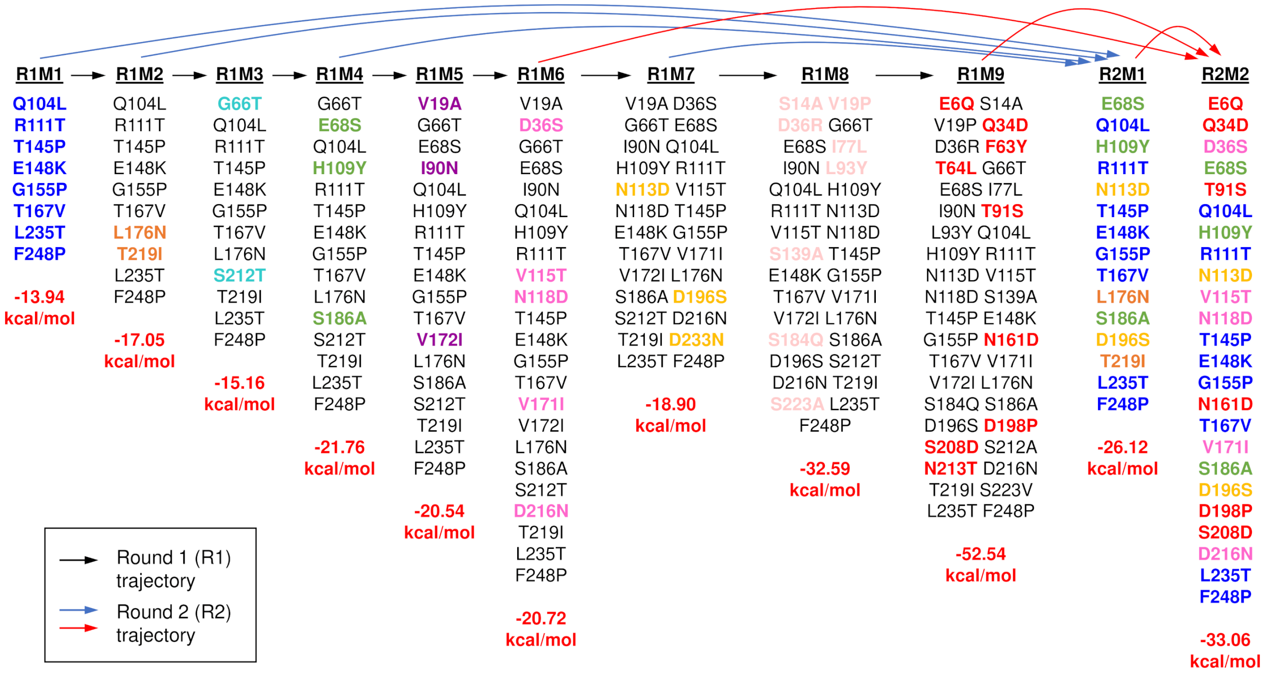

Supplementary Figure 3. Trajectory of mutations in the design of R1 and R2 variants. The mutations that distinguish each variant from WT PHL7 are listed below the variant name. The new mutations that were added in each consecutive variant are displayed with different colors. The Rosetta energy change (in kcal/mol) of each variant with respect to the WT PHL7 structure is indicated below the mutation list. The orders in which mutations were introduced are indicated with arrows. R1 variants were designed in a cumulative fashion (black arrows). R2M1 was designed by combining mutations from R1M1, R1M2, R1M4 and R1M7 (blue arrows). R2M2 was designed by combining mutations from R2M1 (except L176N, T219I) with all mutations from R1M6 and compatible mutations from R1M9 (red arrows).

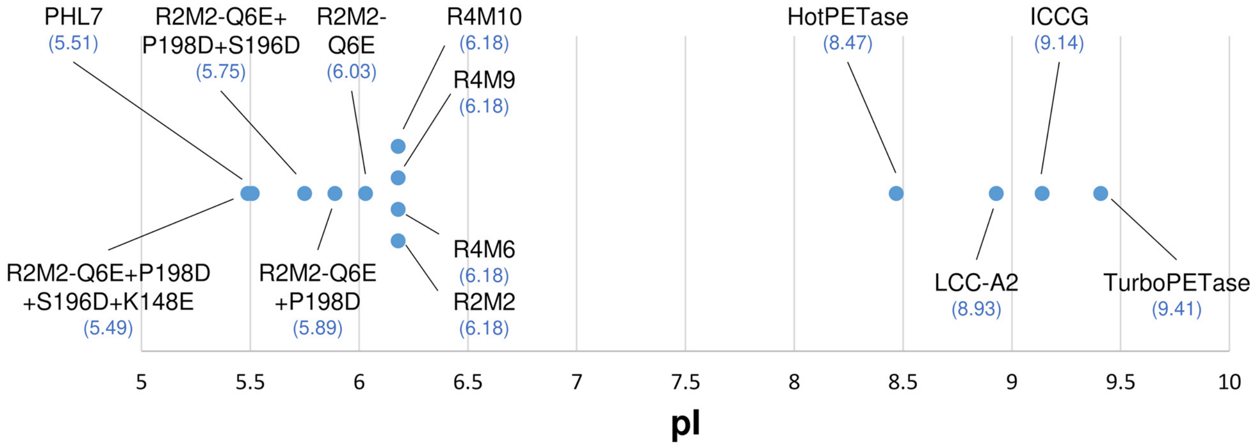

Supplementary Figure 4. Predicted isoelectric points (pI) of PHL7, PHL7 variants and other PET hydrolases. The pI values were calculated from the protein sequences using the Expasy webserver (https://web.expasy.org/protparam/).

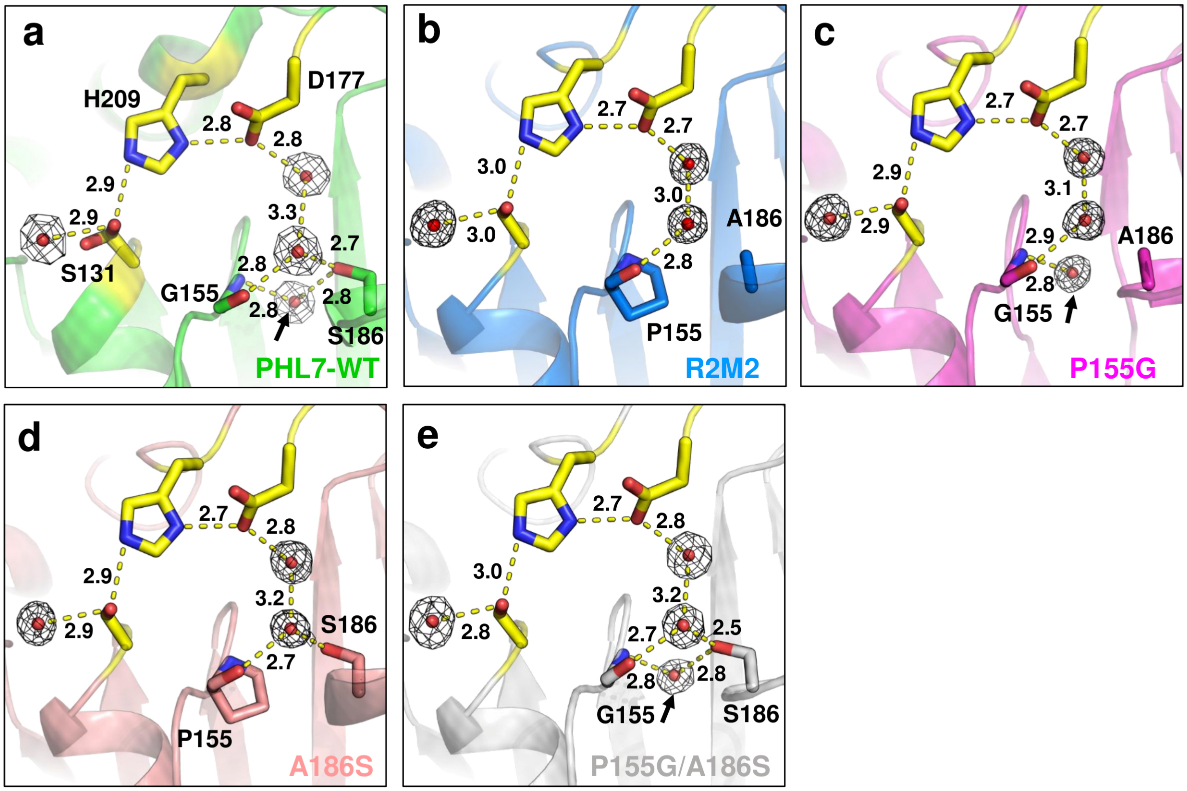

Supplementary Figure 5. Mutations at residue 155 in PHL7 affect the water network which bridges this residue to the catalytic triad. Shown are the sidechains and bridging waters of residues 155, 186 and of the catalytic triad in the X-ray structures of **a** PHL7 WT (PDB 7NEI) (Richter et al. 2023), **b** R2M2, **c** R2M2-P155G, **d** R2M2-A186S, and **e** R2M2-P155G/A186S. Mutation of residue 155 to Pro displaces a water molecule (indicated by an arrow), which reappears after mutation back to Gly. (2Fo-Fc)-type electron density of water molecules is contoured at 1.3 σ_{rmsd} level. Distances are displayed in Å.

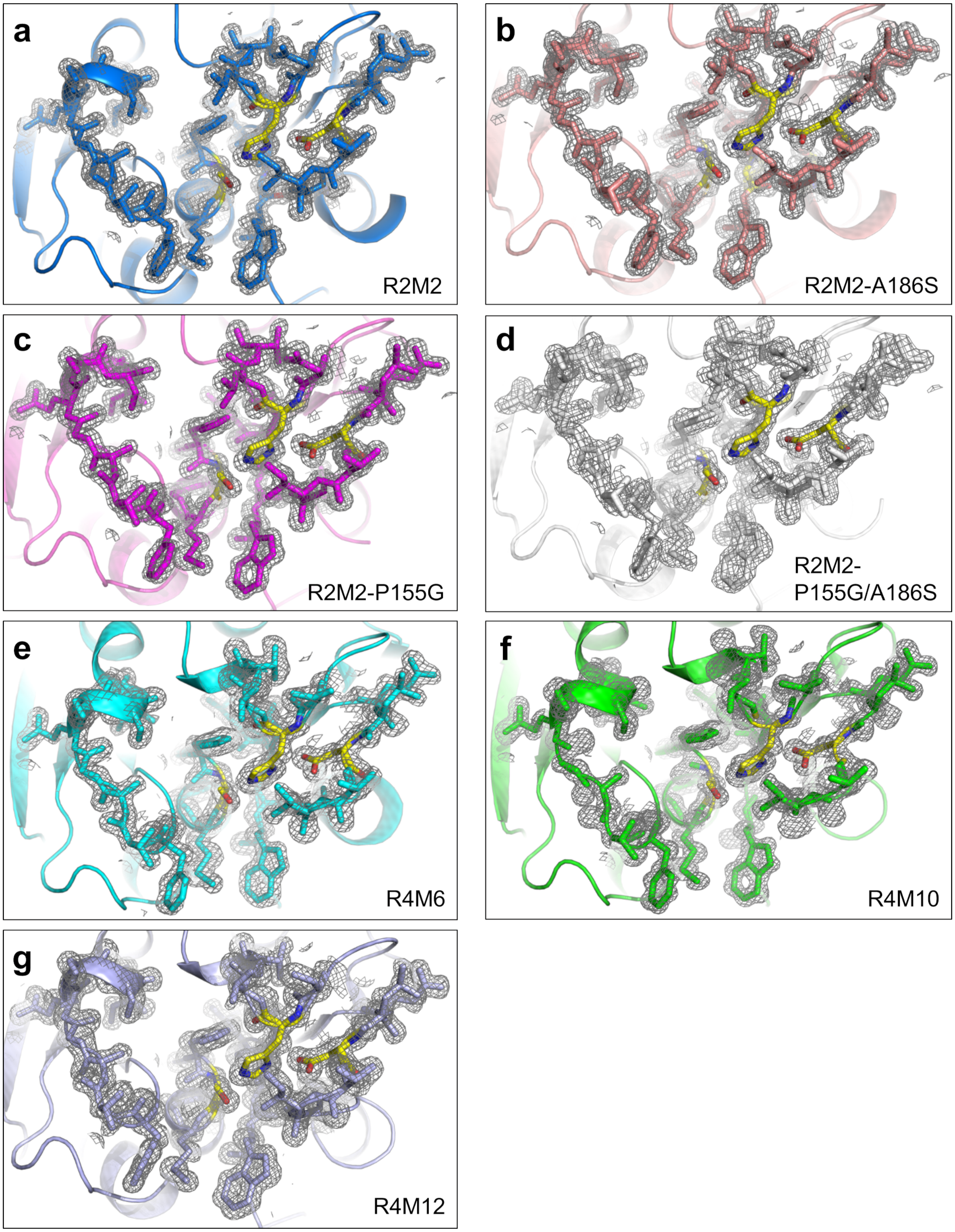

Supplementary Figure 6. Representative electron density maps for PHL7 variants. Representative portions of the electron density are shown for the binding-pocket region of each crystal structure determined in this study. The 2Fo–Fc electron density maps (grey mesh) are contoured at 1.3 σ and overlaid with the refined atomic models. Panels show **a** PHL7-R2M2, **b** R2M2-A186S, **c** R2M2-P155G, **d** R2M2-P155G/A186S, **e** R4M6, **f** R4M10, and **g** R4M12.

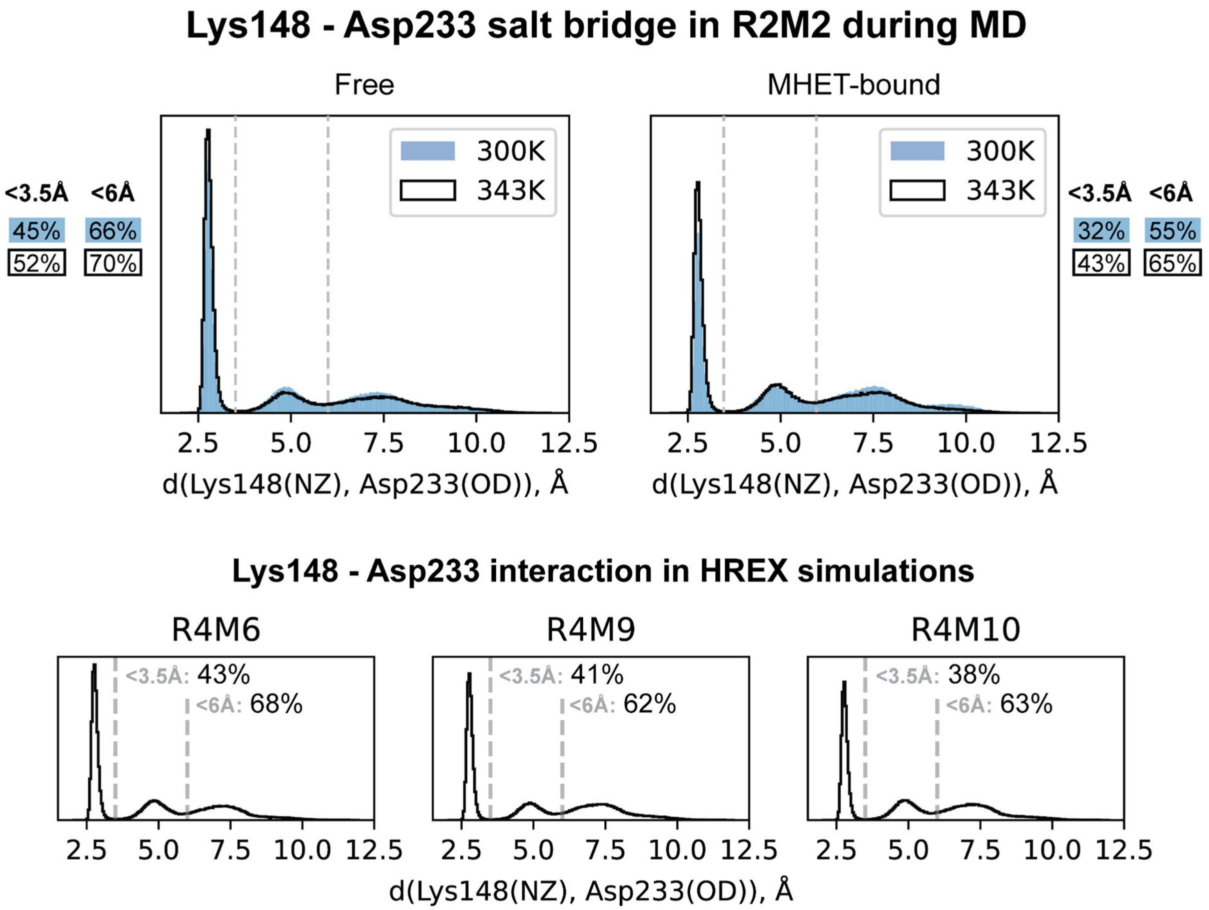

Supplementary Figure 7. K148-D233 interaction in MD simulations of R2M2 (top) and HREX simulations of R4 variants (bottom). Shown is the distance to the closest OD atom of D233, measured over 5*100 ns simulations.

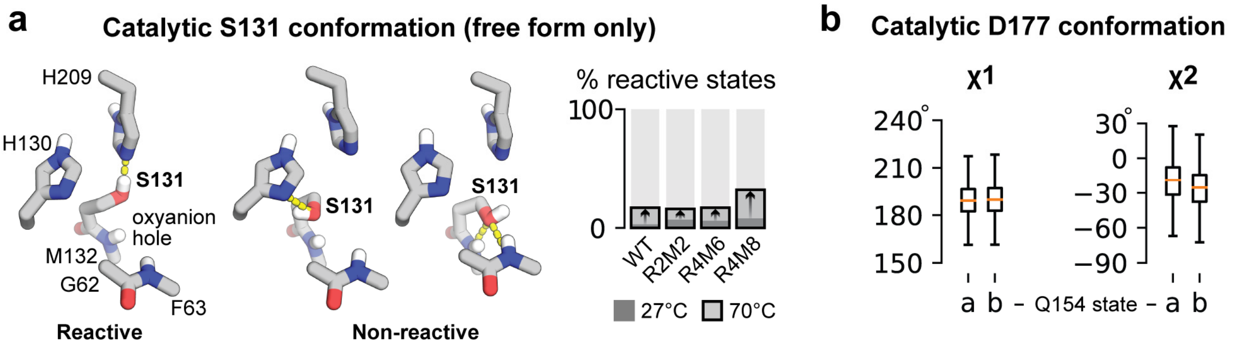

Supplementary Figure 8. Conformations of the catalytic S131 and D177 in MD simulations. **a** In the absence of substrate, PHL7-WT and variants mostly assume non-reactive conformations of S131. **b** The different Q154 rotameric states (referred to as 'a' and 'b' states in Figure 5g) affect the side-chain conformation (χ_1 and χ_2) of the catalytic D177.

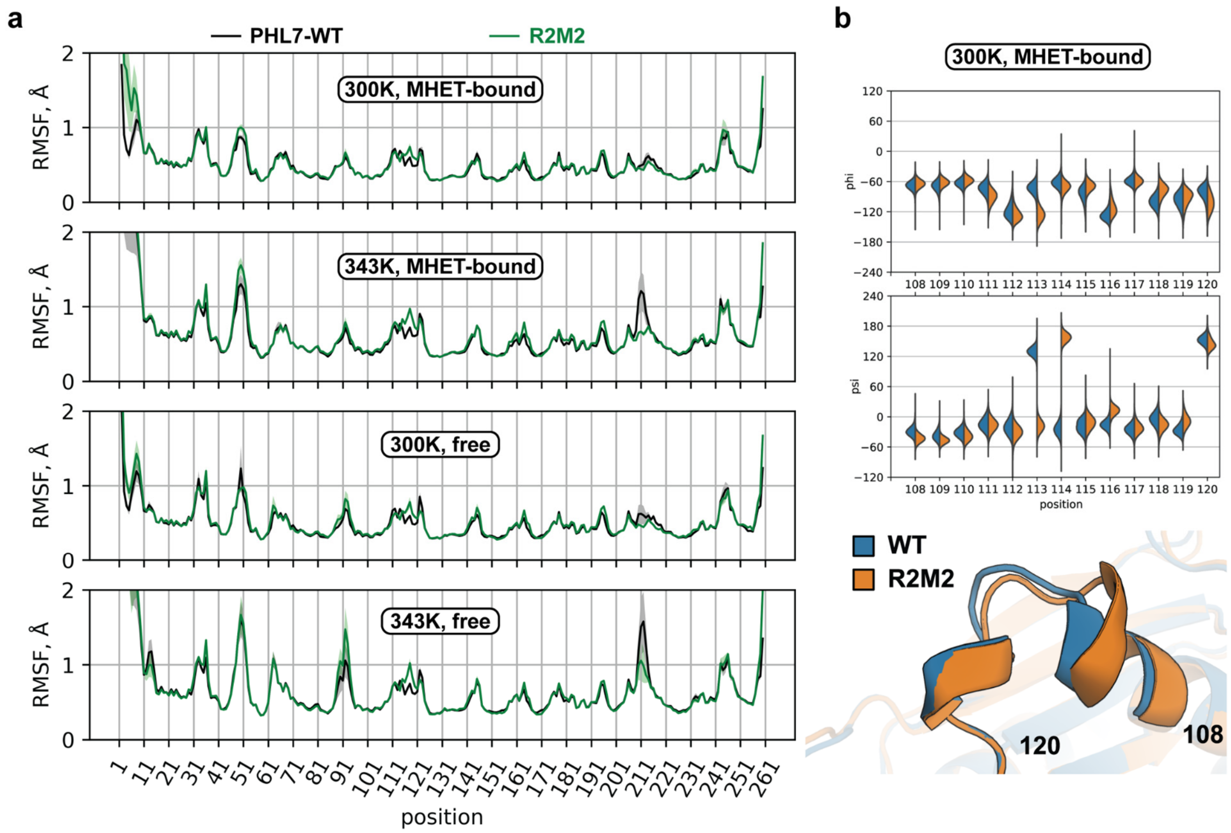

Supplementary Figure 9. Selected dynamical features of WT PHL7 and R2M2. a Root mean square fluctuation (RMSF) of WT PHL7 and R2M2 with and without a ligand, at 27 °C and 70 °C. **b** Backbone angles distributions at the region of greatest difference.

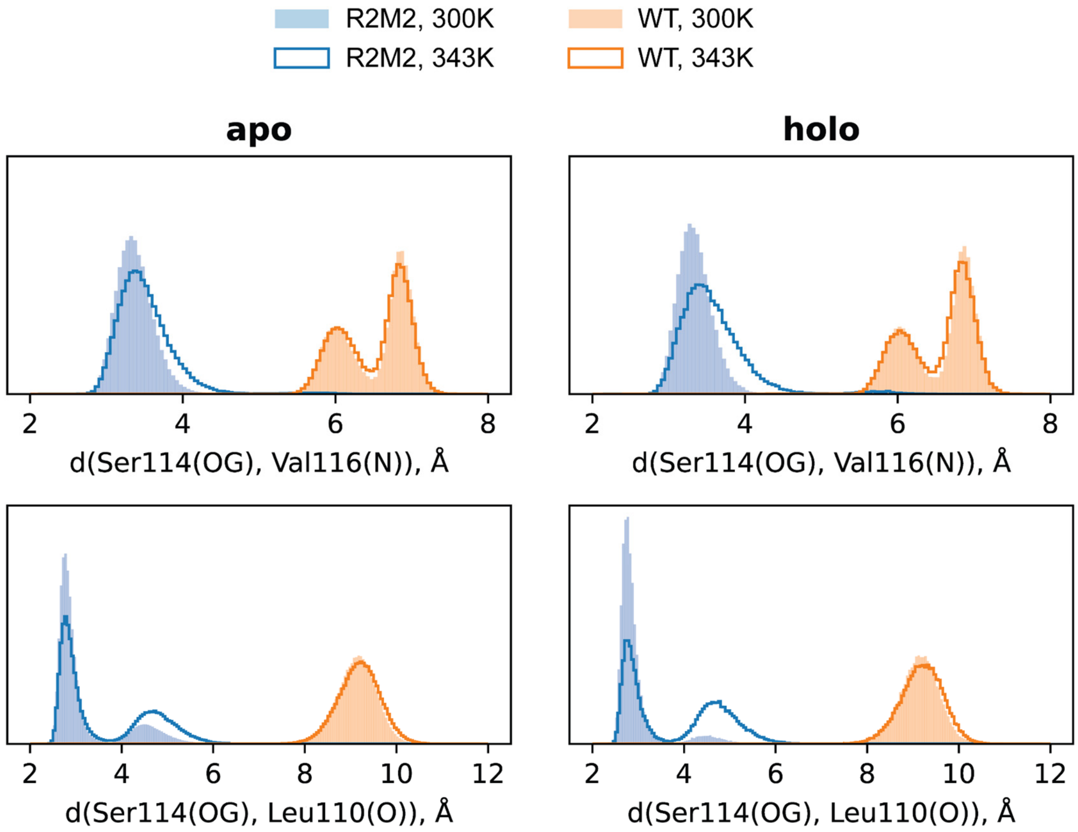

Supplementary Figure 10. Differences in S114 interactions between WT and R2M2 persist in MD simulations. Histograms show the number of frames in which the specified interatomic distances are observed. Shown is data over 5 replicates 100 ns each.

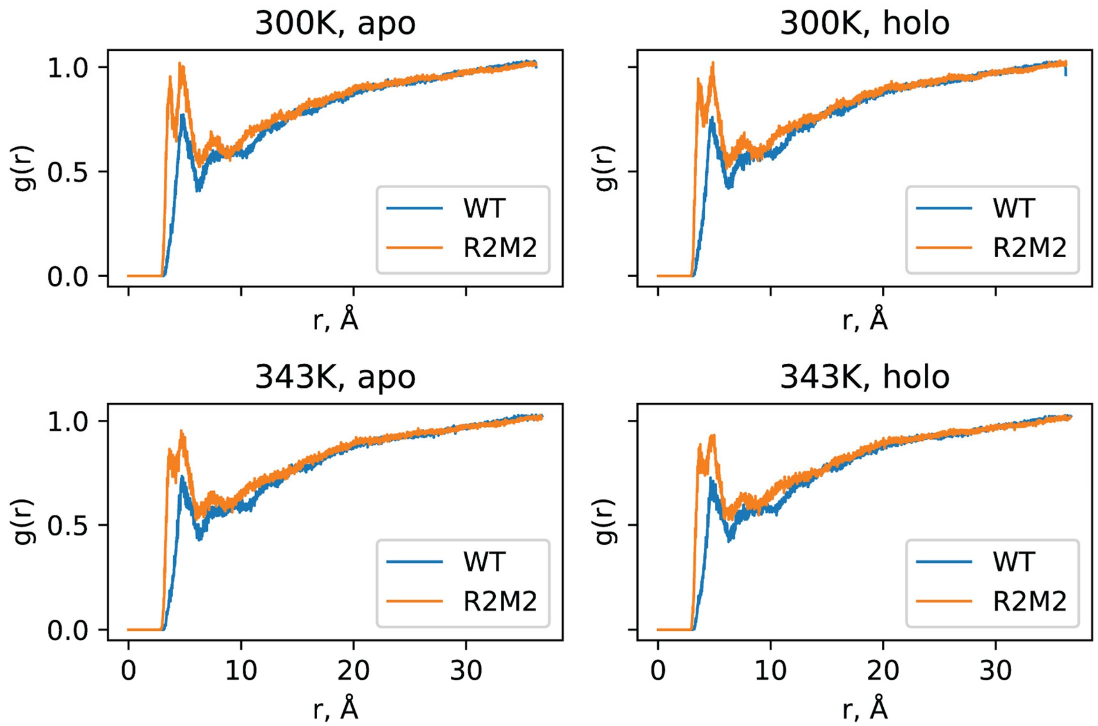

Supplementary Figure 11. Radial distribution functions of water molecules oxygen atoms with respect to the position of CB atom of residue 115. Shown is data over 5 replicates 100 ns each.

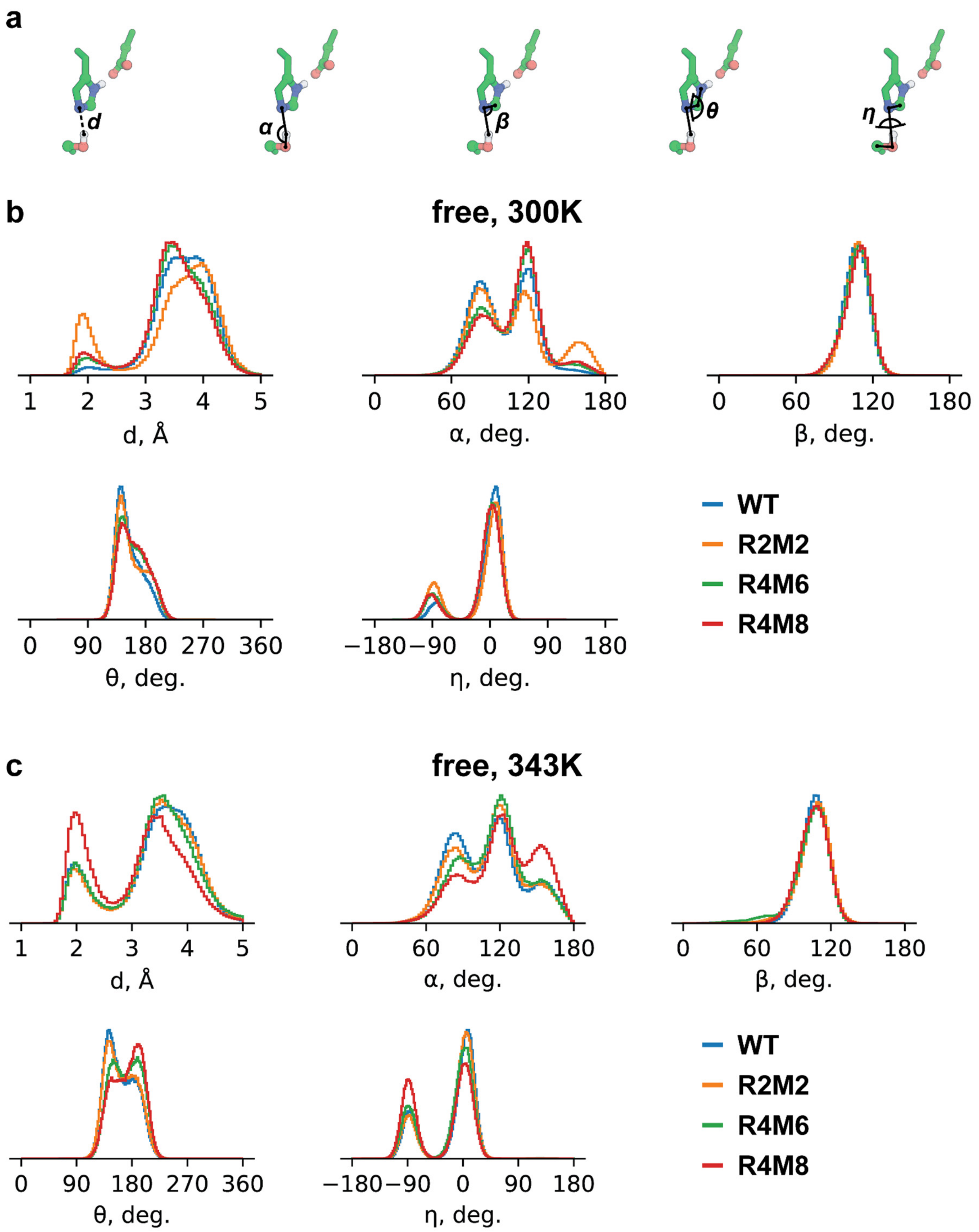

Supplementary Figure 12. Influence of substitutions in R2M2 and R4 mutants on the interaction geometry of catalytic S131 and H209 in MD simulations of free enzyme forms. a Representation of assessed metrics - distances, angles, and torsions. **b** Histograms for free enzyme systems under 300K. **c** Histograms for free enzyme systems under 343K. Shown is data over 5 replicates 100 ns each.

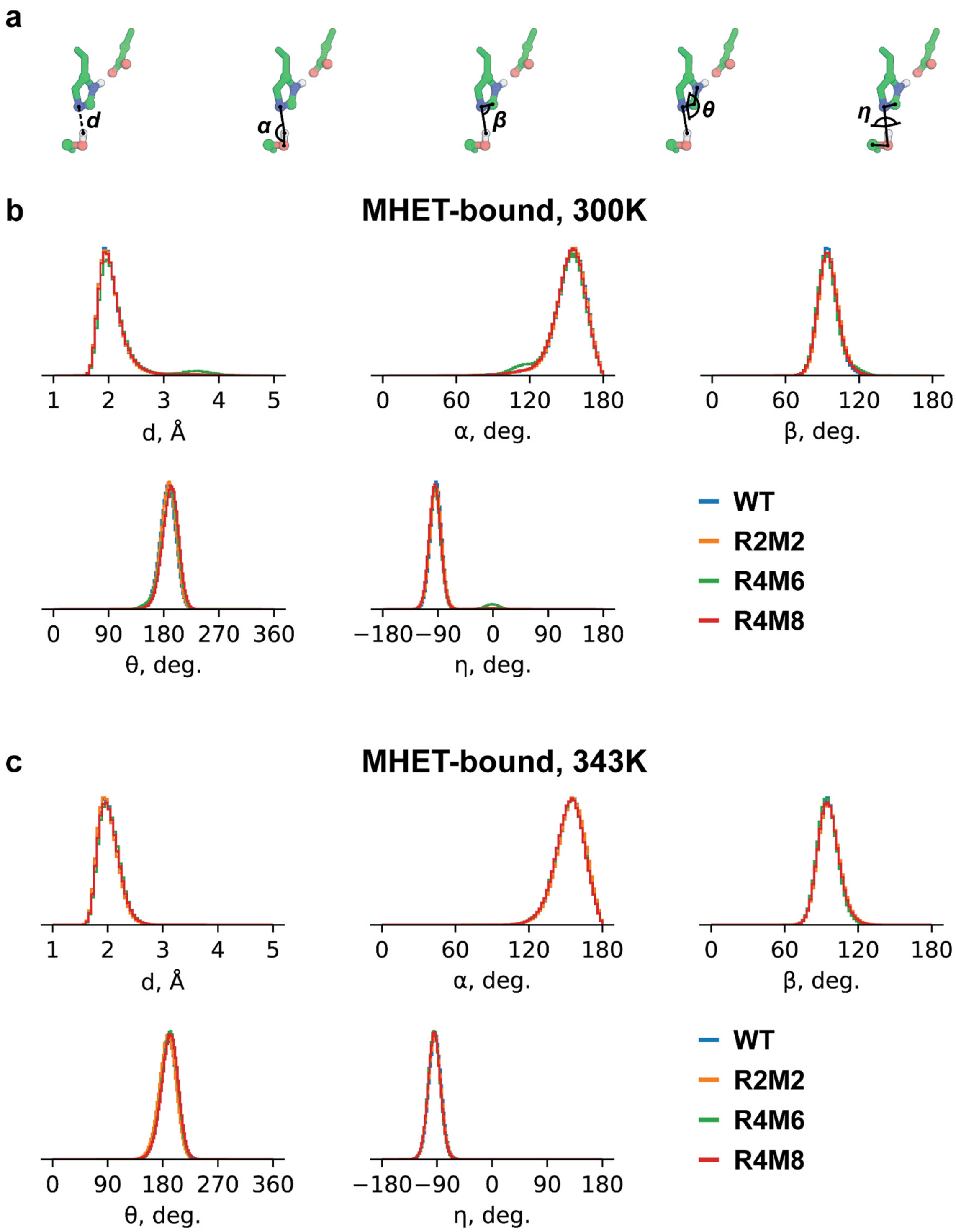

Supplementary Figure 13. Influence of substitutions in R2M2 and R4 mutants on the interaction geometry of catalytic S131 and H209 in MD simulations of substrate-bound enzyme forms. a Representation of assessed metrics - distances, angles, and torsions. **b** Histograms for MHET-bound enzyme systems under 300K. **c** Histograms for MHET-bound enzyme systems under 343K. Shown is data over 5 replicates 100 ns each.

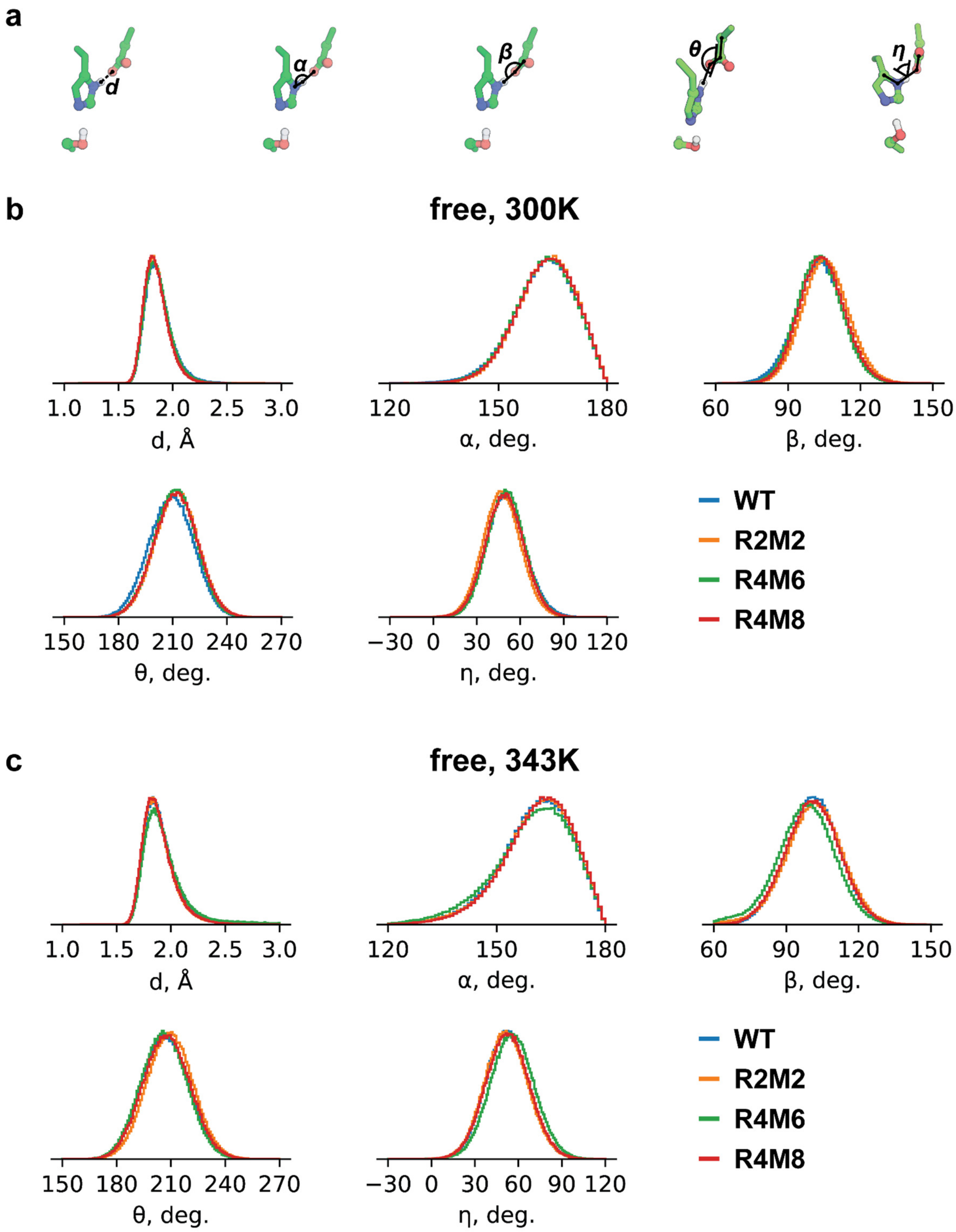

Supplementary Figure 14. Influence of substitutions in R2M2 and R4 mutants on the interaction geometry of catalytic H209 and D177 in MD simulations of free enzyme forms. **a** Representation of assessed metrics - distances, angles, and torsions. **b** Histograms for free enzyme systems under 300K. **c** Histograms for free enzyme systems under 343K. Shown is data over 5 replicates 100 ns each.

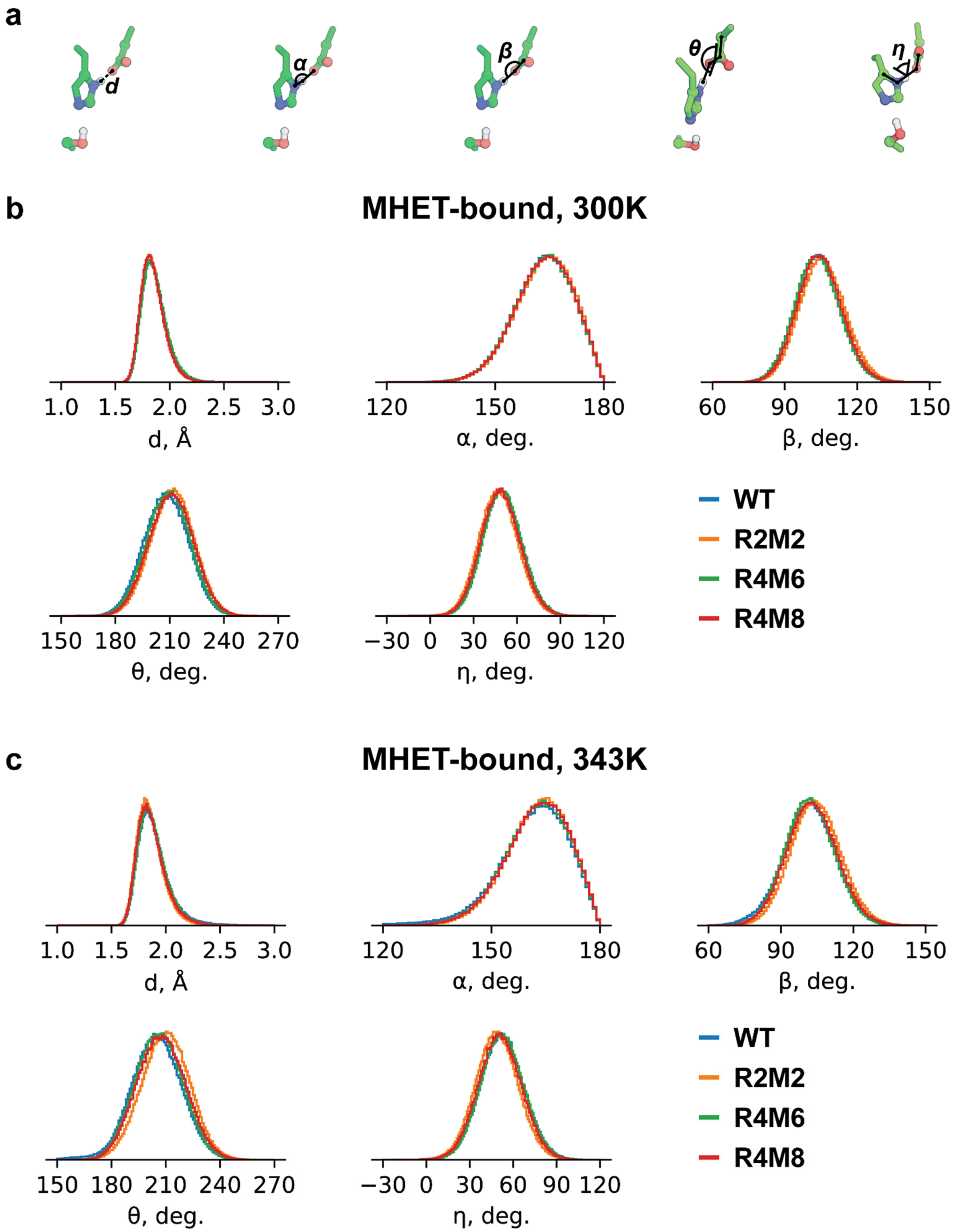

Supplementary Figure 15. Influence of substitutions in R2M2 and R4 mutants on the interaction geometry of catalytic H209 and D177 in MD simulations of substrate-bound enzyme forms. **a** Representation of assessed metrics - distances, angles, and torsions. **b** Histograms for MHET-bound enzyme systems under 300K. **c** Histograms for MHET-bound enzyme systems under 343K. Shown is data over 5 replicates 100 ns each.

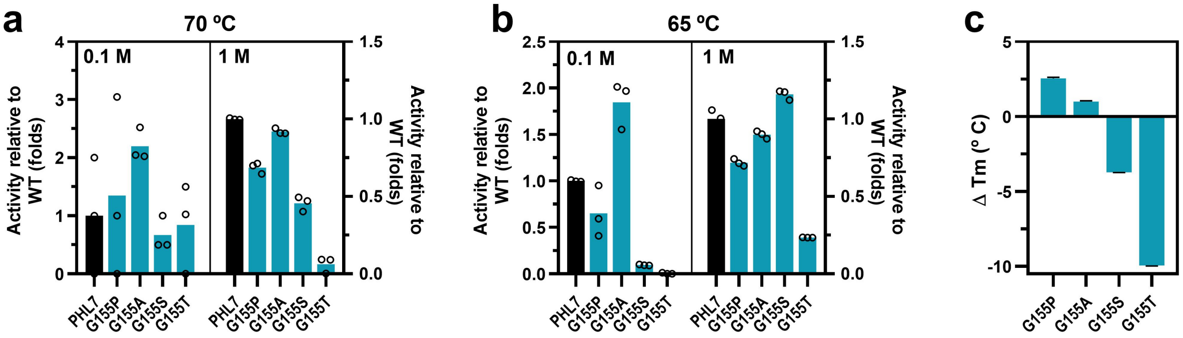

Supplementary Figure 16. PET film degradation efficiency and stability of PHL7 G155X mutants. a-b Fold activity under 0.1 M buffer and 1.0 M buffer conditions relative to PHL7 at 70 °C and 65 ºC. **c** T_m change of G155X mutants relative to WT (T_m = 79.1 °C). Experiments were performed in triplicates (n = 3 independent experiments).

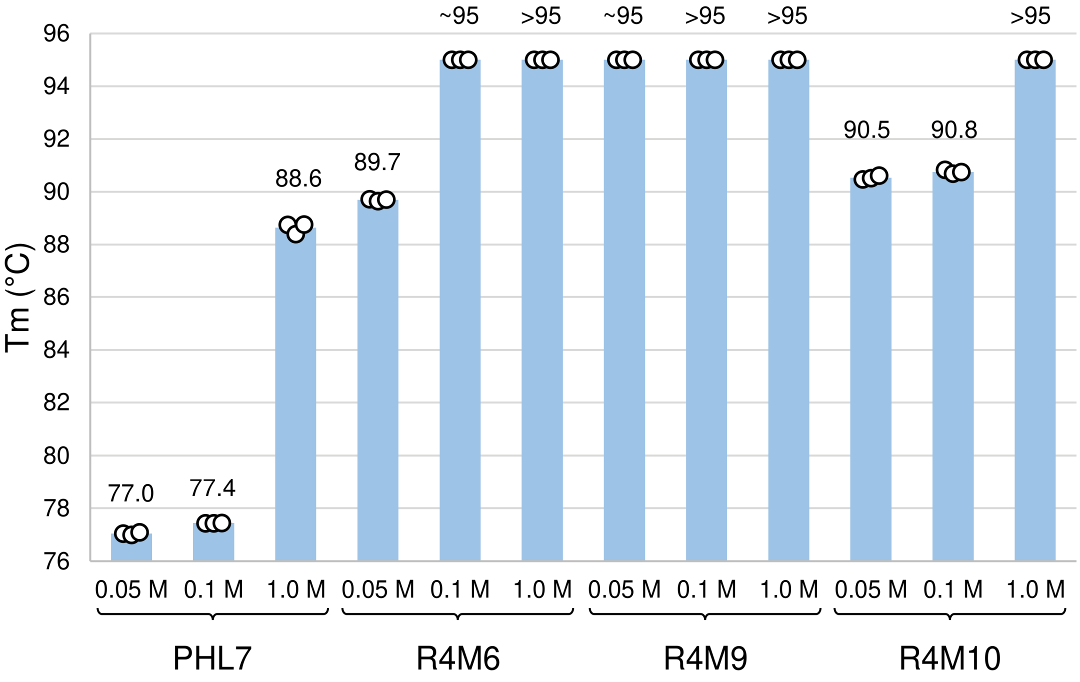

Supplementary Figure 17. Melting temperatures of selected WT PHL7 and selected R4 variants measured in different sodium phosphate buffer concentrations (0.05 M, 0.1 M and 1.0 M).

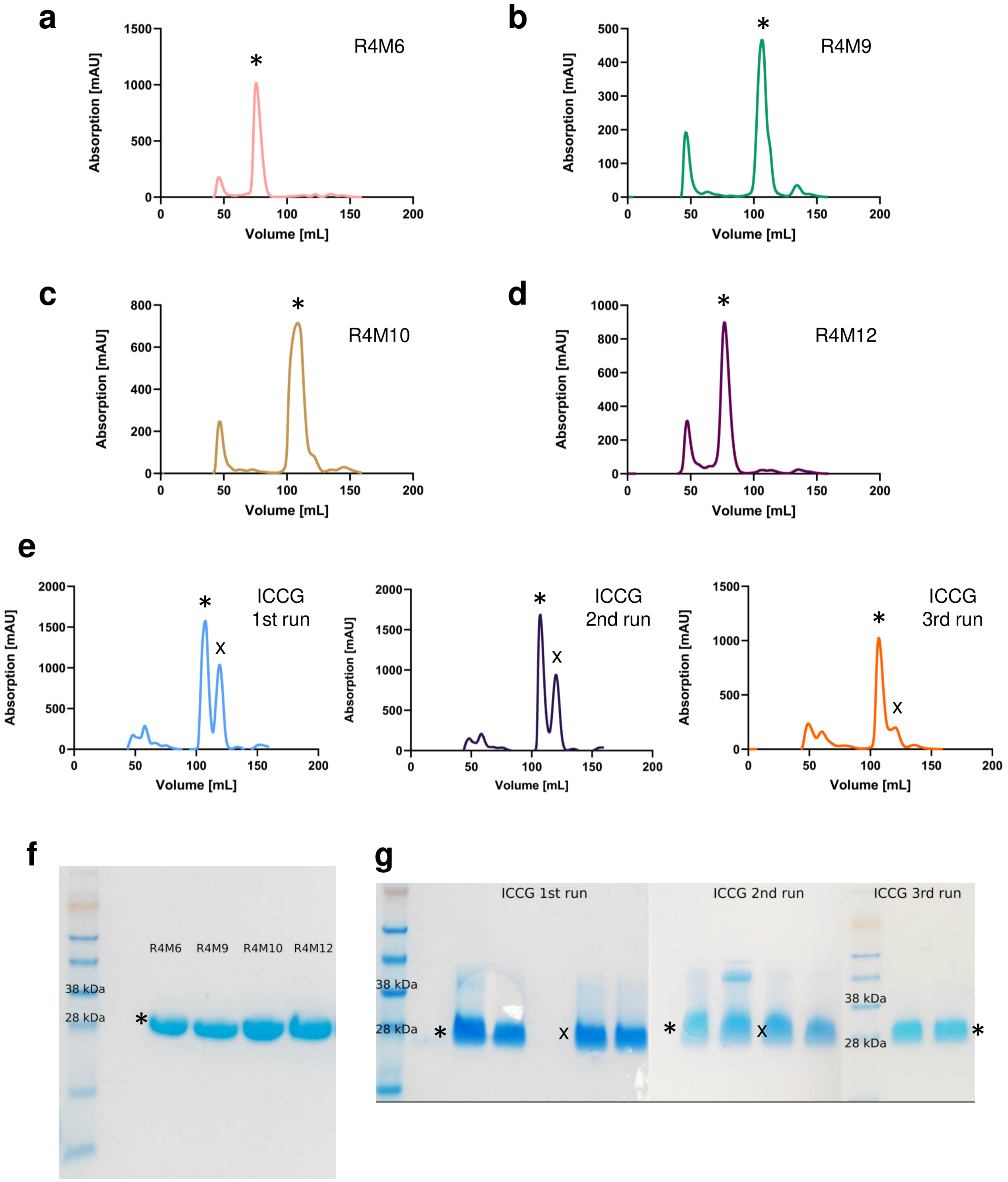

Supplementary Figure 18. Purification of PHL7 R4 mutants and ICCG for testing of PET degradation in bioreactor experiments. a-e SEC chromatograms from the purification of R4M6, R4M9, R4M10, R4M12, and ICCG. The peaks corresponding to the target protein are labeled (*). For ICCG, the target protein eluted in two overlapping peaks (labeled with * and x), which was observed in three independent purification runs. **f, g** SDS PAGE gels showing the SEC peak fractions from the chromatograms in **a-e**. For ICCG, both SEC peak fractions (labeled * and x) were loaded onto the gel.

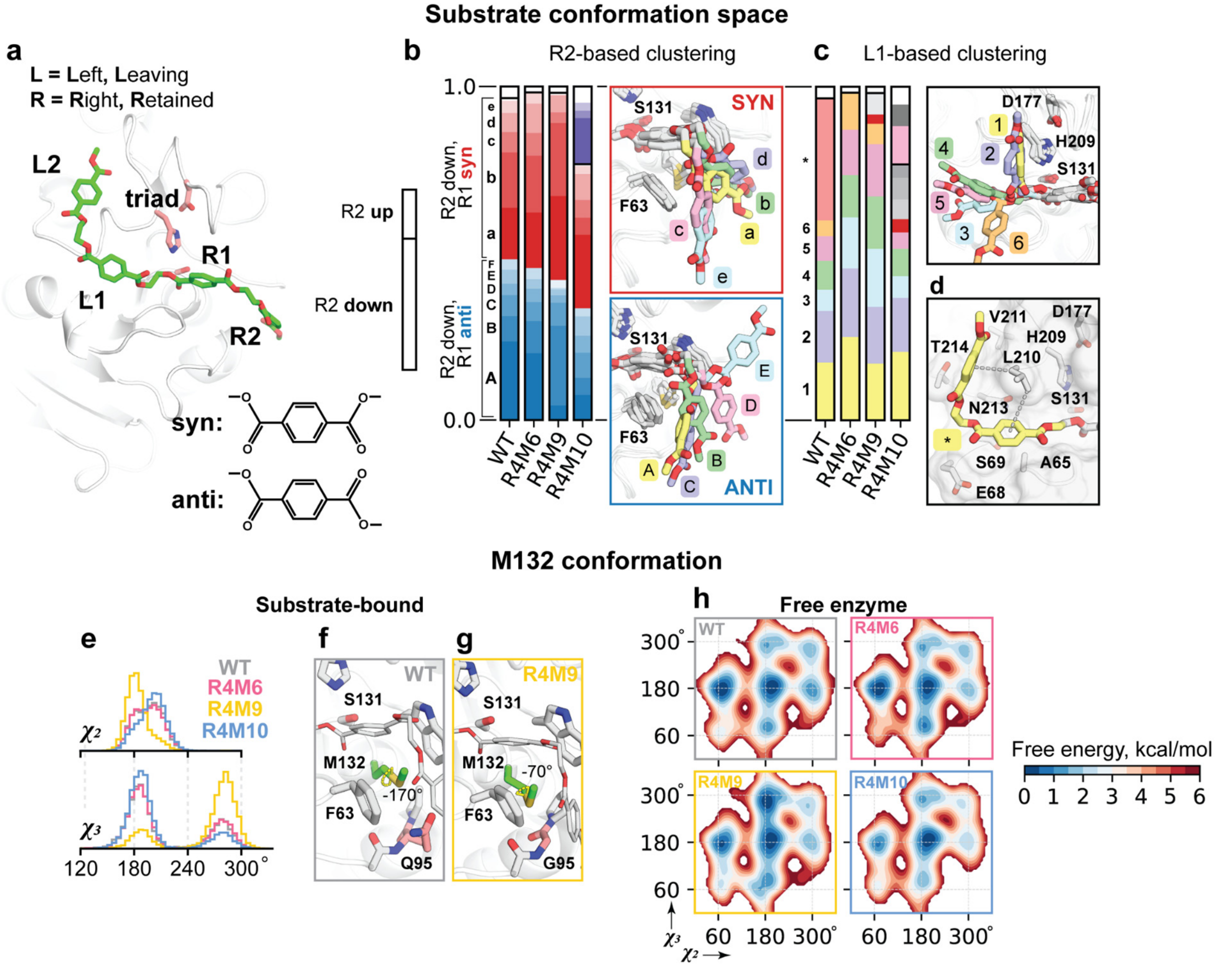

Supplementary Figure 19. MD-derived differences between engineered PHL7 variants in substrate binding modes and M132 conformation. **a** Nomenclature of 4xMHET units. **b** Clusters of R1 locations with populations and representatives. **c** Clusters of L1 locations with populations and representatives. **d** Unique and major PHL7-WT cluster formed by wrapping L2 and L1 around L210. **e** Distributions of M132 side-chain conformations when the substrate is bound. **f, g** examples of M132 conformations. The χ3 angles of M132 are indicated. **h** Free energy profiles of M132 side-chain conformations in the absence of substrate.

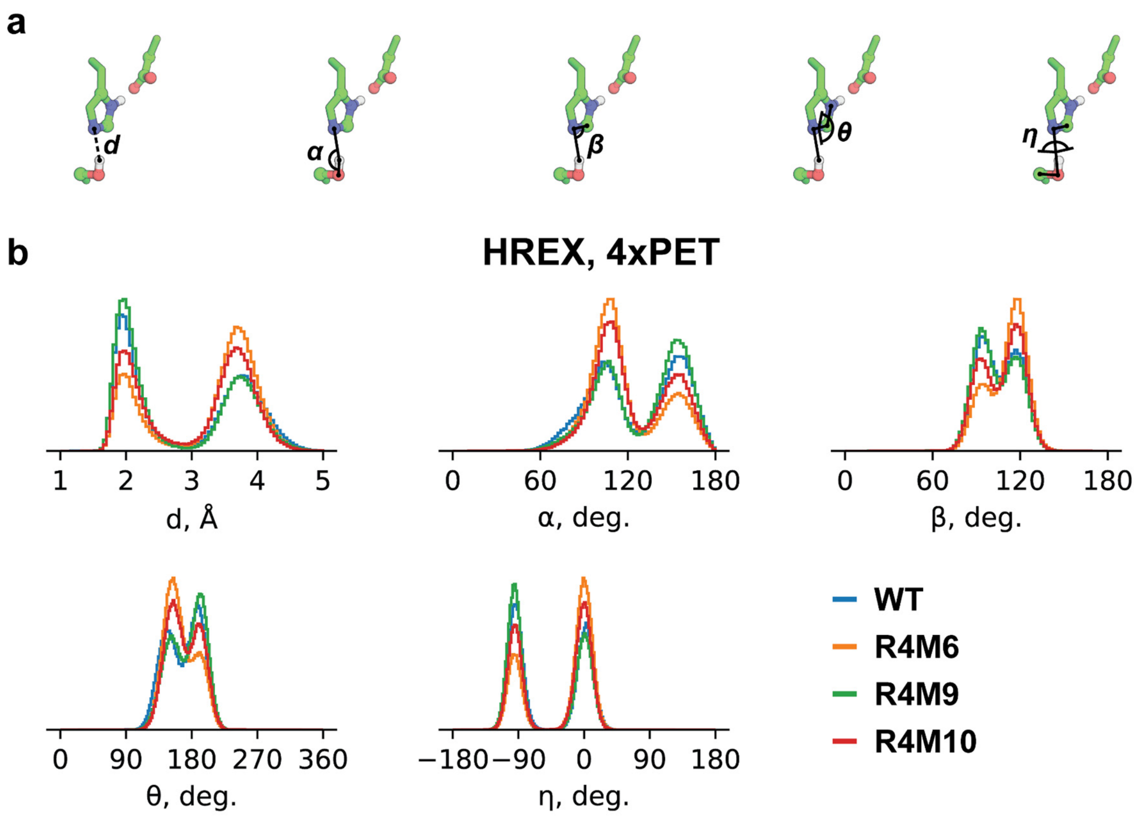

Supplementary Figure 20. Influence of substitutions in R4 variants on the interaction geometry of catalytic S131 and H209 in HREX simulations of 4xMHET-bound enzyme forms. **a** Representation of assessed metrics - distances, angles, and torsions. **b** Histograms for 4xMHET-bound enzyme systems. Shown is data over 5 replicates 100 ns each.

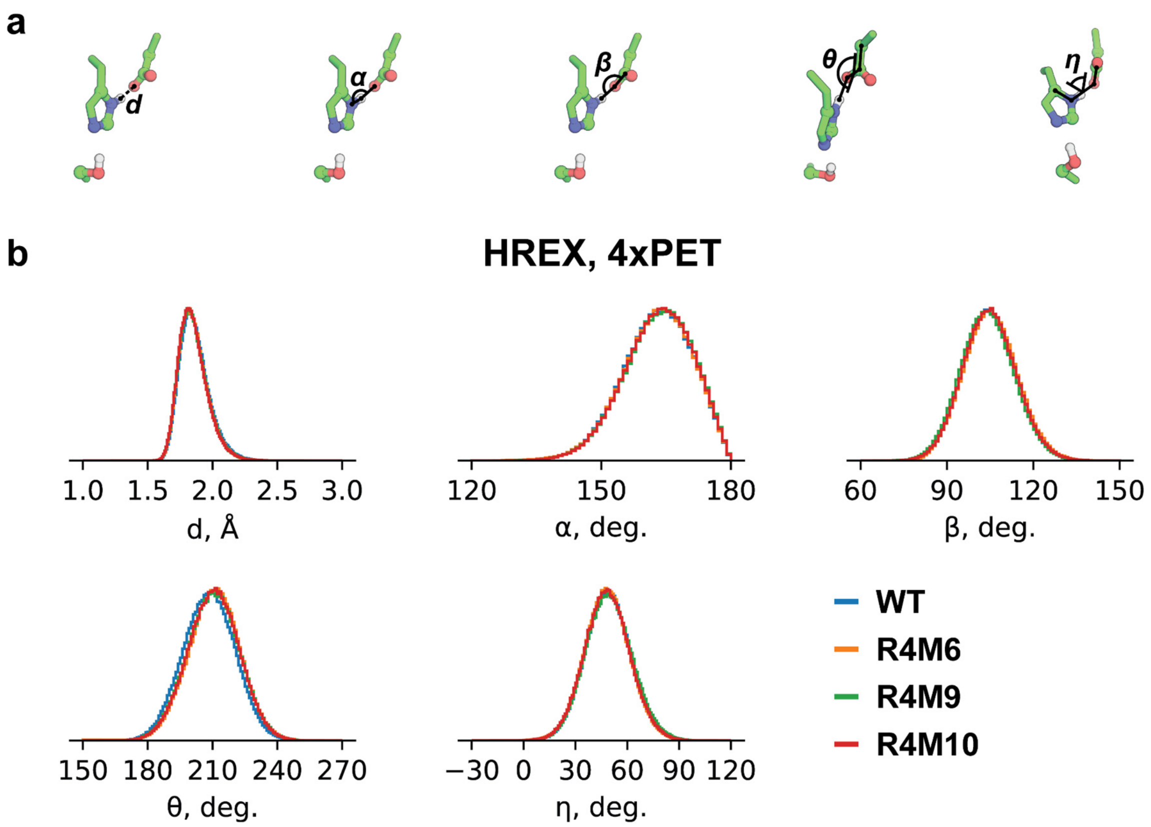

Supplementary Figure 21. Influence of substitutions in R4 variants on the interaction geometry of catalytic H209 and D177 in HREX simulations of substrate-bound enzyme forms. a Representation of assessed metrics, distances and angles. **b** Data for 4xMHET-bound enzyme systems. Shown is data over 5 replicates 100 ns each.

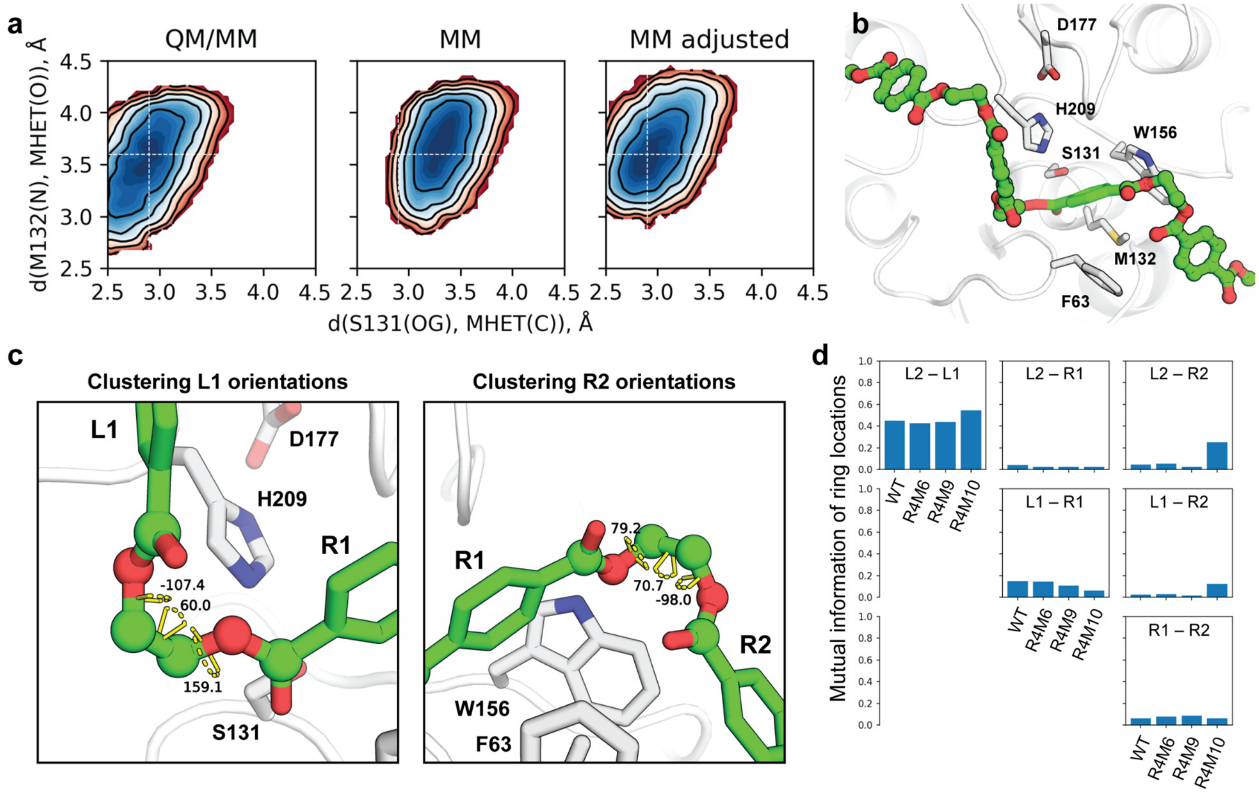

Supplementary Figure 22. Supporting figures for supplementary MD methods. a Improvement of MHET-PHL7 interaction geometry in the oxyanion hole-bound state. **b** "Hot" region for the local HREX experiment. Atoms shown as spheres constitute the "hot" region. **c** Torsion angles used to perform clustering. Atoms used to define those angles are shown as spheres. **d** Interdependence of the four TPA ring locations. Shown are the values of the mutual information function calculated for the locations of the TPA benzene rings from the 4xMHET substrate. Each ring location is represented by the coordinate of the center of mass of the 6 constituent carbon atoms, obtained from the superposed trajectories from HREX simulations.

Supplementary References

1. Khrenova MG, Tsirelson VG, Nemukhin AV. Dynamical properties of enzyme–substrate complexes disclose substrate specificity of the SARS-CoV-2 main protease as characterized by the electron density descriptors. Phys Chem Chem Phys. 2020 Sep 9;22(34):19069–79.

2. Gillet N, Elstner M, Kubař T. Coupled-perturbed DFTB-QM/MM metadynamics: Application to proton-coupled electron transfer. The Journal of Chemical Physics. 2018 Jun 27;149(7):072328.

3. Zlobin A, Belyaeva J, Golovin A. Challenges in Protein QM/MM Simulations with Intra-Backbone Link Atoms. J Chem Inf Model. 2023 Jan 23;63(2):546–60.

4. Bannwarth C, Ehlert S, Grimme S. GFN2-xTB—An Accurate and Broadly Parametrized Self-Consistent Tight-Binding Quantum Chemical Method with Multipole Electrostatics and Density-Dependent Dispersion Contributions. J Chem Theory Comput. 2019 Mar 12;15(3):1652–71.

5. Westerlund AM, Delemotte L. InfleCS: Clustering Free Energy Landscapes with Gaussian Mixtures. J Chem Theory Comput. 2019 Dec 10;15(12):6752–9.

Uncropped gel images corresponding to Supplementary Figures 18f and 18g.

Note that the gels contain additional protein samples not corresponding to the R4 variants or ICCG; these lanes were cropped in the figure panels 18f and 18g to display only the relevant samples.

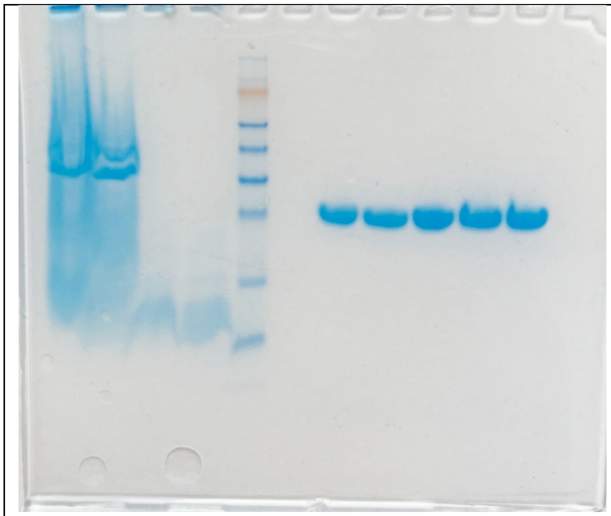

Uncropped gel for supplementary figure 18f.

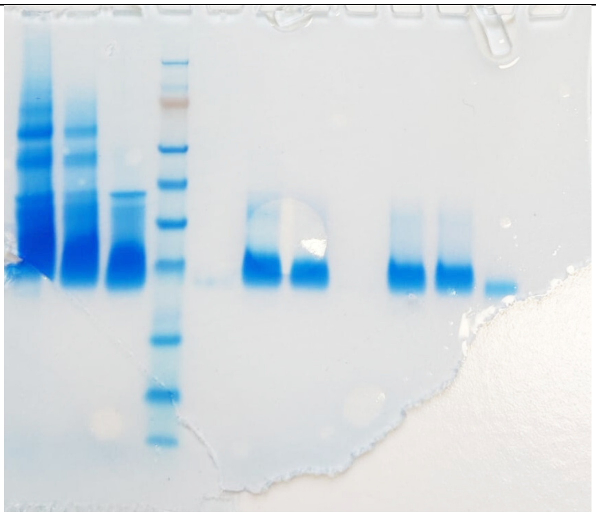

Uncropped gel for supplementary Figure 18g, 1st ICCG purification run.

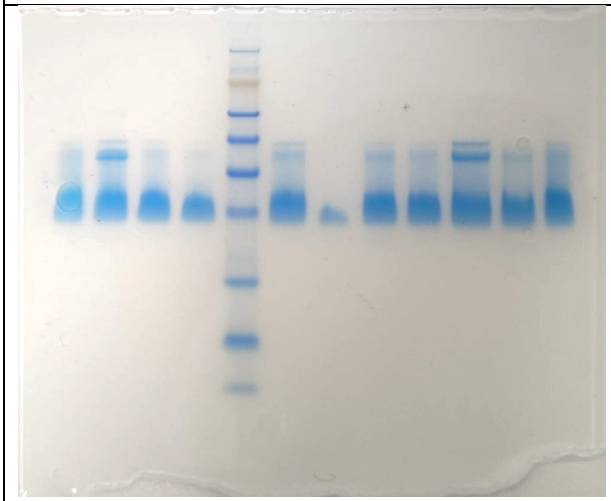

Uncropped gel for supplementary Figure 18g, 2nd ICCG purification run.

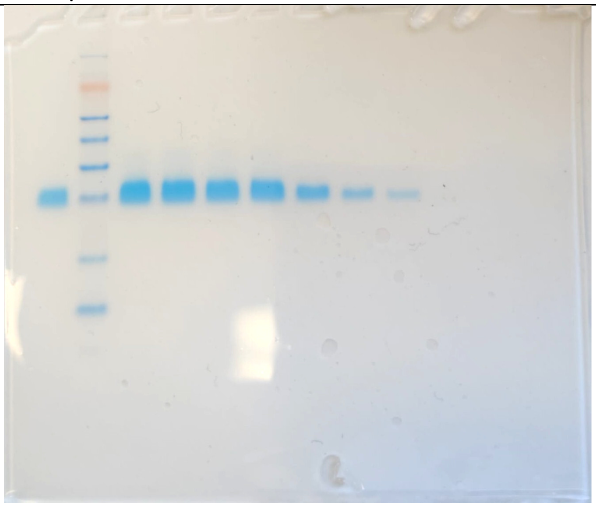

Uncropped gel for supplementary Figure 18g, 3rd ICCG purification run.
