# Supplementary material for: Computational engineering of the polyester hydrolase PHL7 for efficient poly(ethylene terephthalate) degradation in biocatalytic recycling processes
Source: Nat Commun. 2026 May 15;17:4370. doi: 10.1038/s41467-026-70868-4 (PMC13179365; doi:10.1038/s41467-026-70868-4)
Supplement: Supplementary file 2 — Reporting Summary [file 41467_2026_70868_MOESM2_ESM.pdf]

nature portfolio

Corresponding author(s): Georg Künze, Christian Sonnendecker

Last updated by author(s): Feb 15, 2026

Reporting Summary

Nature Portfolio wishes to improve the reproducibility of the work that we publish. This form provides structure for consistency and transparency in reporting. For further information on Nature Portfolio policies, see our Editorial Policies and the Editorial Policy Checklist.

Statistics

For all statistical analyses, confirm that the following items are present in the figure legend, table legend, main text, or Methods section.

n/a	Confirmed	
☐	☒	The exact sample size (*n*) for each experimental group/condition, given as a discrete number and unit of measurement
☐	☒	A statement on whether measurements were taken from distinct samples or whether the same sample was measured repeatedly
☒	☐	The statistical test(s) used AND whether they are one- or two-sided<br>*Only common tests should be described solely by name; describe more complex techniques in the Methods section.*
☒	☐	A description of all covariates tested
☒	☐	A description of any assumptions or corrections, such as tests of normality and adjustment for multiple comparisons
☐	☒	A full description of the statistical parameters including central tendency (e.g. means) or other basic estimates (e.g. regression coefficient) AND variation (e.g. standard deviation) or associated estimates of uncertainty (e.g. confidence intervals)
☒	☐	For null hypothesis testing, the test statistic (e.g. *F*, *t*, *r*) with confidence intervals, effect sizes, degrees of freedom and *P* value noted<br>*Give P values as exact values whenever suitable.*
☒	☐	For Bayesian analysis, information on the choice of priors and Markov chain Monte Carlo settings
☒	☐	For hierarchical and complex designs, identification of the appropriate level for tests and full reporting of outcomes
☒	☐	Estimates of effect sizes (e.g. Cohen's *d*, Pearson's *r*), indicating how they were calculated

Our web collection on statistics for biologists contains articles on many of the points above.

Software and code

Policy information about availability of computer code

Data collection	UNICORN™ 7 (for Cytiva Äkta system), Nanotemper Prometheus™ Panta software, IMATadvanced and Universal EC-Fitter software for ISX-3v2 impedance analyzer (Sciospec Scientific Instruments), Gen5 data analysis software for BioTek Synergy Mx Microplate Reader, Gromacs (version 2023), Plumed (version 2.9), XDS (version 10), STARANISO (version 2.3.74), CCP4i2 (CCP4 version 8.0), REFMAC (version 5.8.0425), Coot (version 0.9.8.93 and 0.9.8.95), Phenix (version 1.20.1_4487 and 1.21.2_5419).
Data analysis	Nanotemper Prometheus™ Panta software for analyzing thermal protein unfolding curves<br>Gromacs software (version 2023) for analyzing MD data<br>XDS (version 10), CCP4i2 (CCP4 version 8.0) and REFMAC (version 5.8.0425) for analyzing X-ray datasets<br>Coot (version 0.9.8.93 and 0.9.8.95) and Phenix (version 1.20.1_4487 and 1.21.2_5419) for X-ray structure model building<br>PlayMolecule (webserver: http://www.playmolecule.org/) for fitting MD force field parameters<br>Matplotlib (version 3.10.0) and Numpy (version 2.1.3) libraries accessible through Anaconda software (version 2024.06) for data plotting<br>Graphpad Prism 7 for data plotting and statistical analysis<br>Microsoft Excel 2019 for analyzing weight loss and PET degradation data and for data plotting<br>PyMOL (Schrodinger, LLC, version 2.6.2 and version 1.3) for making protein structure visualizations

For manuscripts utilizing custom algorithms or software that are central to the research but not yet described in published literature, software must be made available to editors and reviewers. We strongly encourage code deposition in a community repository (e.g. GitHub). See the Nature Portfolio guidelines for submitting code & software for further information.

Data

Policy information about availability of data

All manuscripts must include a data availability statement. This statement should provide the following information, where applicable:

- Accession codes, unique identifiers, or web links for publicly available datasets
- A description of any restrictions on data availability
- For clinical datasets or third party data, please ensure that the statement adheres to our policy

The coordinates and structure factors of PHL7-R2M2, R2M2-P155G, R2M2-A186S, R2M2-P155G/A186S, R4M6, R4M10, and R4M12 have been deposited in the Protein Data Bank under accession codes 9QNM, 9QT8, 9QV8, 9QVA, 9QYA, 9QYB, and 9QYC, respectively. The customized scripts used for running and analyzing the MD simulations of PHL7 variants are available via a Zenodo repository under https://doi.org/10.5281/zenodo.18650810. All other computational and experimental data generated and analyzed in the study are included in the manuscript, in the supporting material or in the supplementary source data files, and are available from the corresponding author(s) upon request. Source Data are provided with this paper.

Research involving human participants, their data, or biological material

Policy information about studies with human participants or human data. See also policy information about sex, gender (identity/presentation), and sexual orientation and race, ethnicity and racism.

Reporting on sex and gender	n/a
Reporting on race, ethnicity, or other socially relevant groupings	n/a
Population characteristics	n/a
Recruitment	n/a
Ethics oversight	n/a

Note that full information on the approval of the study protocol must also be provided in the manuscript.

Field-specific reporting

Please select the one below that is the best fit for your research. If you are not sure, read the appropriate sections before making your selection.

☒ Life sciences ☐ Behavioural & social sciences ☐ Ecological, evolutionary & environmental sciences

For a reference copy of the document with all sections, see nature.com/documents/nr-reporting-summary-flat.pdf

Life sciences study design

All studies must disclose on these points even when the disclosure is negative.

Sample size	For weight-loss assays, melting-temperature measurements, EIS-based PET degradation assays, and inverse Michaelis–Menten kinetic analyses, a minimum of three replicates (n = 3) was conducted to determine mean values and standard deviations. The selected sample sizes are consistent with standard practice in the field and no formal statistical power calculations were performed to predetermine sample sizes. Bioreactor PET degradation experiments were performed in duplicate for R4M6, R4M9, and R4M10 (n = 2), in quintuplicate for ICCG (n = 5), and as a single replicate for R4M12 (n = 1). Sample sizes were selected based on practical considerations, including the time- and resource-intensive nature of bioreactor experiments, and were deemed sufficient to assess relative performance trends.
Data exclusions	No data has been excluded
Replication	All in vitro experiments that report explicit standard deviation (SD) were performed with a minimum of three replicates. Reproducibility was verified by repeating experiments independently using different enzyme preparations and substrate batches. Results obtained for specific enzyme constructs (PHL7 variants) were reproduced across independent measurements and validated using complementary assay formats, including weight-loss assays and EIS-based PET degradation assays. All replication attempts were successful, and no findings were identified that could not be reproduced.
Randomization	Sample allocation was not randomized, as this was not relevant for the present study. In biochemical and enzymatic experiments, investigators must know the identity of the protein variants being expressed, purified, and characterized in order to perform the experiments and interpret the results.
Blinding	Blinding was not applied in this study, as is standard practice in biochemical and enzymatic assays where investigators must know the identity of the samples (e.g., the specific protein variant under investigation) to perform and interpret the experiments. Accordingly, blinding was not relevant for this study and does not affect the objectivity of the quantitative measurements reported.

Reporting for specific materials, systems and methods

We require information from authors about some types of materials, experimental systems and methods used in many studies. Here, indicate whether each material, system or method listed is relevant to your study. If you are not sure if a list item applies to your research, read the appropriate section before selecting a response.

Materials & experimental systems

n/a	Involved in the study
☒	☐ Antibodies
☒	☐ Eukaryotic cell lines
☒	☐ Palaeontology and archaeology
☒	☐ Animals and other organisms
☒	☐ Clinical data
☒	☐ Dual use research of concern
☒	☐ Plants

Methods

n/a	Involved in the study
☒	☐ ChIP-seq
☒	☐ Flow cytometry
☒	☐ MRI-based neuroimaging

Plants

Seed stocks

Report on the source of all seed stocks or other plant material used. If applicable, state the seed stock centre and catalogue number. If plant specimens were collected from the field, describe the collection location, date and sampling procedures.

Novel plant genotypes

Describe the methods by which all novel plant genotypes were produced. This includes those generated by transgenic approaches, gene editing, chemical/radiation-based mutagenesis and hybridization. For transgenic lines, describe the transformation method, the number of independent lines analyzed and the generation upon which experiments were performed. For gene-edited lines, describe the editor used, the endogenous sequence targeted for editing, the targeting guide RNA sequence (if applicable) and how the editor was applied.

Authentication

Describe any authentication procedures for each seed stock used or novel genotype generated. Describe any experiments used to assess the effect of a mutation and, where applicable, how potential secondary effects (e.g. second site T-DNA insertions, mosiacism, off-target gene editing) were examined.
